# Targeting β-catenin degradation with GSK3β inhibitors induces cell death in acute lymphoblastic leukemia

**DOI:** 10.1038/s43018-025-01093-z

**Published:** 2026-01-08

**Authors:** Kadriye Nehir Cosgun, Huda Jumaa, Mark E. Robinson, Zhangliang Cheng, Salim Oulghazi, Kohei Kume, David Fonseca Arce, Nikol Agadzhanian, Klaus M. Kistner, Etienne Leveille, Elsa Drivet, Fang Yu, Zhijian Qian, Joo Y. Song, Wing-Chung Chan, Liang Xu, Gang Xiao, M. Mark Taketo, Shalin Kothari, Matthew S. Davids, Hilde Schjerven, Julia Jellusova, Markus Müschen

**Affiliations:** 1 Center of Molecular and Cellular Oncology, Yale University, New Haven, CT USA; 2 Signalling Research Centres BIOSS and CIBSS, Albert Ludwigs University of Freiburg, Freiburg, Germany; 3 Department of Immunobiology, Yale University, New Haven, CT USA; 4 Institute of Clinical Chemistry and Pathobiochemistry, School of Medicine and Health, Technical University Munich, Munich, Germany; 5 TranslaTUM, Center for Translational Cancer Research, Technical University of Munich, Munich, Germany; 6 Department of Immunology, Oslo University Hospital, Oslo, Norway; 7 KG Jebsen Center for B-Cell Malignancies, Oslo University, Oslo, Norway; 8 Department of Systems Biology, City of Hope Comprehensive Cancer Center Biomedical Research Center, Monrovia, CA USA; 9 Department of Pathology, City of Hope Comprehensive Cancer Center, Duarte, CA USA; 10 Kyoto University Yoshida-Honmachi Sakyo, Kyoto, Japan; 11 Department of Medical Oncology, Dana-Farber Cancer Institute, Harvard Medical School, Boston, MA USA; 12 Department of Laboratory Medicine, University of California, San Francisco, San Francisco, CA USA; 13 Helen Diller Family Comprehensive Cancer Center, University of California, San Francisco, San Francisco, CA USA; 14 Present Address: College of Life Sciences and School of Medicine, Zhejiang University, Hangzhou, China

**Keywords:** Lymphoma, Acute lymphocytic leukaemia, Proteasome, Transcriptional regulatory elements, Cancer

## Abstract

As part of canonical Wnt signaling, T cell factor (TCF)–β-catenin complexes promote MYC-dependent proliferation. Lesions of the β-catenin protein degradation machinery are common oncogenic drivers. Here, we show that B cell acute lymphoblastic leukemia (B-ALL) lacks these mutations and critically depends on unencumbered β-catenin protein degradation. Compared to solid tumors, we found that mouse and human B-ALL express β-catenin protein at much lower levels; β-catenin protein was constitutively phosphorylated by glycogen synthase kinase 3B (GSK3β) and poised for proteasomal degradation. Instead of TCF–β-catenin complexes to activate MYC, β-catenin paired with B lymphoid Ikaros and NuRD complex factors, resulting in MYC repression and acute cell death. To leverage β-catenin protein degradation as a previously unrecognized vulnerability in B-ALL, we validated GSK3β inhibition in patient-derived xenograft models in vivo. CRISPR screens confirmed β-catenin protein degradation as a central mechanistic target of established GSK3β inhibitors. As several GSK3β inhibitors achieved favorable safety profiles in clinical trials, our results provide a rationale for repurposing these compounds for persons with refractory B cell malignancies.

## Abstract (web summary)

Cosgun et al. show that, in B cell leukemia, β-catenin expression is maintained at low levels through glycogen synthase kinase 3B (GSK3β)-mediated phosphorylation. Inhibition of GSK3β results in β-catenin–Ikaros–NuRD complex formation, leading to B-ALL cell death through MYC repression.

## Main

**Extended Data Fig. 1:**
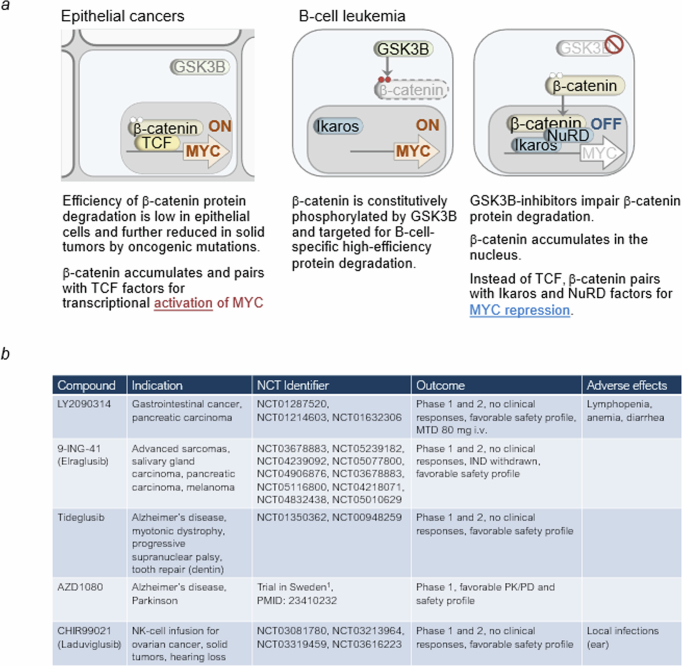
Targeting high-efficiency β-catenin protein degradation in B-cell leukemia. (**a**) In epithelial cancers (left) efficiency of β-catenin protein degradation is low and further reduced by oncogenic mutations. β-catenin accumulates and pairs with TCF factors for transcriptional activation of MYC. In B-cell leukemia (middle) β-catenin is constitutively phosphorylated by GSK3B and targeted for B-cell-specific high-efficiency protein degradation. GSK3B-inhibitors impair β-catenin protein degradation (right) and β-catenin accumulates in the nucleus. Instead of TCF, β-catenin pairs with Ikaros and NuRD factors for MYC repression (right). **b**) Summary of Phase I and Phase II clinical trials with GSK3β inhibitors for a variety of clinical indications. In a total of 20 clinical trials, all tested small molecule inhibitors achieved favorable safety and PK/PD profiles at micromolar plasma concentrations (Cmax). None of the inhibitors achieved clinical responses. Moderate adverse effects included diarrhea, anemia and lymphopenia.

The canonical Wnt–β-catenin pathway regulates fundamental processes including embryonic development, organogenesis and tissue homeostasis^1^. β-catenin protein degradation is initiated by phosphorylation of N-terminal β-catenin residues^2^ (S33, S37, T41 and S45) by glycogen synthase kinase 3B (GSK3β) and casein kinase 1α (CK1α) and the scaffolding proteins Axin1/2 and APC^3,4^, followed by ubiquitination^5^ and proteasomal degradation. Unphosphorylated β-catenin accumulates in nucleus^6^ and pairs with T cell factors (TCFs)^7,8^ to promote transcriptional activation of Wnt targets including MYC^9,10^. Transcriptional activation of MYC by TCF–β-catenin complexes represents a central oncogenic driver in multiple cancer types^9,10^. In contrast to epithelial, neuronal and mesenchymal lineages^1^, β-catenin deletion was dispensable for hematopoietic and B cell development^11–14^. Instead, targeted removal of GSK3β and CK1α phosphorylation sites to stabilize β-catenin^(S33^^;S45)+/fl^ mice^2^ impaired hematopoietic multilineage differentiation and lymphoid development^15,16^. Conversely, in colon cancer, melanoma and other epithelial cancers, defective β-catenin protein degradation accelerated MYC-dependent proliferation and malignant transformation^17–20^. Genetic mouse models of myeloid malignancies demonstrated that β-catenin was required for the initiation of acute myeloid leukemia (AML)^21^ and chronic myeloid leukemia (CML)^22,23^. However, the development of murine B cell acute lymphoblastic leukemia (B-ALL) was unperturbed by deletion of β-catenin^23^. Two studies on the effects of Wnt3A-dependent β-catenin accumulation on proliferation of B-ALL cells reported conflicting results^24,25^. B-ALL cells with TCF3–PBX1 fusion aberrantly overexpress the WNT16 ligand^26^. However, subsequent work showed that WNT16 does not promote canonical β-catenin signaling^27^. Here, we show that B-ALL cells have evolved and critically depend on high-efficiency mechanisms of β-catenin protein degradation (Extended Data Fig. 1a). GSK3β has a central role in mediating high-efficiency β-catenin protein degradation^3,4^. Small-molecule inhibitors of GSK3β have been developed for the treatment of solid tumors and neurological conditions (Extended Data Fig. 1b)^28–33^. While these small-molecule inhibitors achieved favorable safety profiles in early-phase clinical trials, our preclinical experiments provide a rationale to repurpose these existing GSK3β inhibitors to subvert β-catenin protein degradation to improve outcomes for persons with refractory B cell malignancies.

## Results

### Lack of β-catenin protein expression in B cell malignancies

**Fig. 1:**
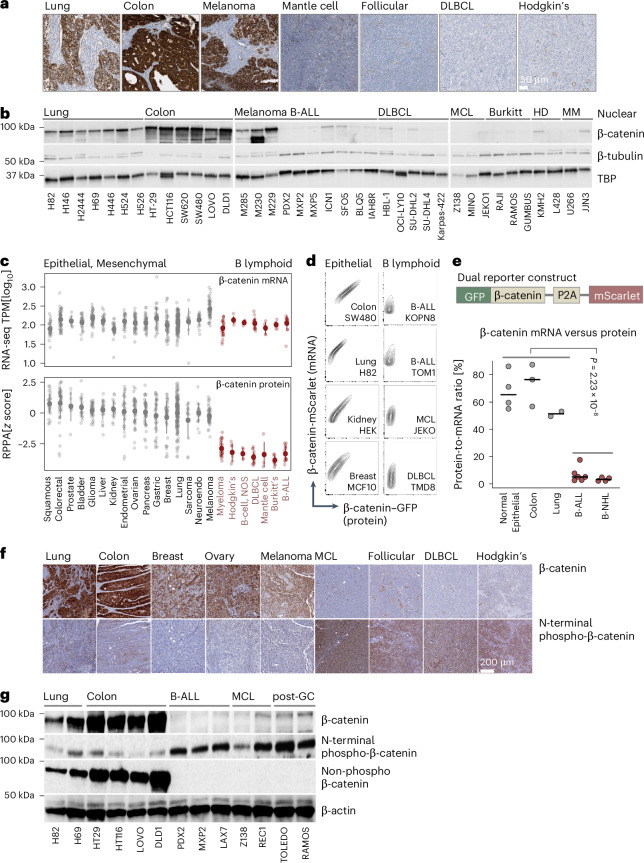
B lymphoid cells express β-catenin mRNA but lack β-catenin protein expression. **a**, Representative immunohistochemical staining for β-catenin (brown) on tissue microarrays from lung cancer (*n* = 15 tumors, biological replicates), colon cancer (*n* = 25 tumors, biological replicates) and malignant melanoma (*n* = 5 tumors, biological replicates) in comparison to MCL (*n* = 26 tumors, biological replicates), follicular lymphoma (*n* = 38 tumors, biological replicates), DLBCL (*n* = 35 tumors, biological replicates) and HD (*n* = 44 tumors, biological replicates). **b**, Western blot analysis for β-catenin, β-tubulin and TBP on nuclear fractions of lung cancer (*n* = 7 cell lines, biological replicates), colon cancer (*n* = 6 cell lines, biological replicates), malignant melanoma (*n* = 3 cell lines, biological replicates), B-ALL (*n* = 7 cell lines, biological replicates), DLBCL (*n* = 5 cell lines, biological replicates), MCL (*n* = 3 cell lines, biological replicates), Burkitt’s lymphoma (*n* = 3 cell lines, biological replicates), HD (*n* = 2 cell lines, biological replicates) and multiple myeloma (MM; *n* = 2 cell lines, biological replicates). **c**, Top, transcriptional analysis of 779 cancer cell lines by RNA-seq (PRJNA523380) for the expression of CTNNB1 in 692 solid tumor cell lines (biological replicates) compared to 87 B cell leukemia and lymphoma cell lines (biological replicates). Bottom, protein levels of β-catenin were measured by RPPA (CCLE2019) in B cell leukemia and lymphoma cell lines (*n* = 86 cell lines, biological replicates), as well as solid tumor cell lines (*n* = 646 cell lines, biological replicates). TPM, transcripts per million. **d**,**e**, Fluorescent protein stability assay to compare rate of β-catenin degradation in epithelial cell lines, including colon (SW480) and lung (H82) cancer cell lines and cells derived from normal epithelial tissues including human embryonic kidney (HEK) and normal breast epithelial cells (MCF10) and in B lymphoid cells including B-ALL (KOPN8 and TOM1), MCL (JEKO) and DLBCL (TMD8). GFP-tagged WT β-catenin (protein) or GFP tag was expressed along with mScarlet (mRNA) reporter and stability of β-catenin was assessed by flow cytometric measurement of GFP and mScarlet signal. **d**, Representative FACS plots are shown for cells expressing β-catenin–GFP fusion protein along with mScarlet mRNA reporter. **e**, Frequencies of cells expressing β-catenin–GFP fusion protein within mScarlet^+^ cells are shown and an unpaired two-tailed *t*-test was performed to compare β-catenin protein and mRNA expression in all B lymphoid cells (B-ALL and B-NHL; *n* = 10 cell lines, biological replicates) versus all epithelial cells (normal epithelial, colon cancer and lung cancer; *n* = 9 cell lines, biological replicates) (*P* = 2.23 × 10^−8^). **f**, Representative immunohistochemical staining for β-catenin (brown; top) and β-catenin phosphorylated by GSK3β (S37, brown; bottom) on tissue microarrays from lung cancer (*n* = 41 tumors, biological replicates), colon cancer (*n* = 13 tumors, biological replicates), breast cancer (*n* = 58 tumors, biological replicates), ovarian cancer (*n* = 9 tumors, biological replicates), malignant melanoma (*n* = 13 tumors, biological replicates), MCL (*n* = 21 tumors, biological replicates), follicular lymphoma (*n* = 54 tumors, biological replicates), DLBCL (*n* = 47 tumors, biological replicates) and HD (*n* = 20 tumors, biological replicates) using hematoxylin (blue) as the counterstain. **g**, Western blot analysis for total β-catenin, N-terminal phosphorylated β-catenin by GSK3β (S33, S37 and T41) and nonphosphorylated (active) β-catenin in a panel of lung (*n* = 2 cell lines, biological replicates) and colon (*n* = 4 cell lines, biological replicates) cancer cell lines in comparison to human B-ALL (*n* = 3 cell lines, biological replicates), MCL (*n* = 2 cell lines, biological replicates) and postgerminal center lymphoma (*n* = 2 cell lines, biological replicates) (three technical repeats). The exposure time for total and nonphosphorylated β-catenin was 185 s; the exposure time for N-terminal phosphorylated β-catenin (S33, S37 and T41) was 1,199 s. Source data

**Extended Data Fig. 2:**
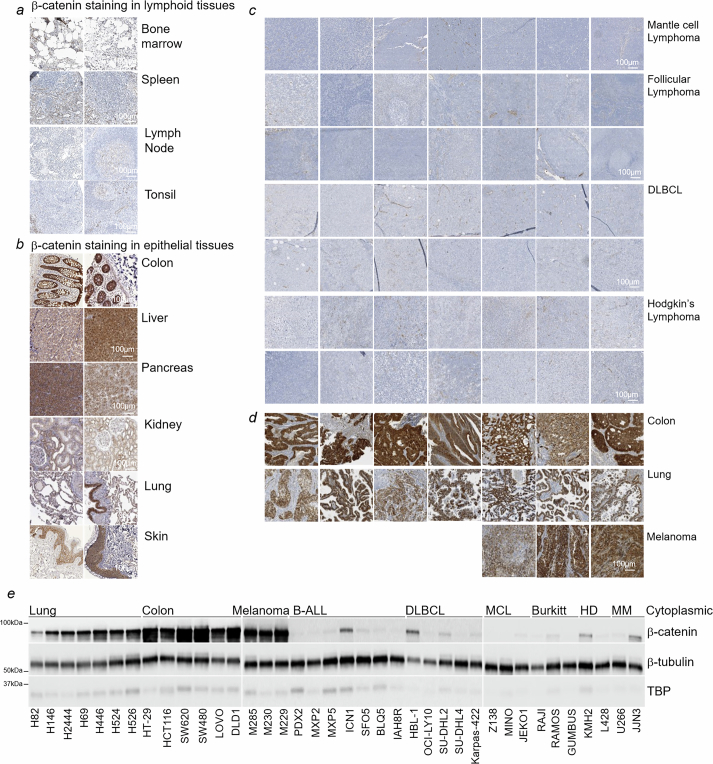
Lack of β-catenin expression and activity in B-lymphoid cells. (**a-b**) Immunohistochemical staining for β-catenin expression (brown, hematoxylin counterstain) in (**a**) human lymphoid tissues (*left*), including bone marrow (n = 7 tissues, biological replicates), spleen (n = 6 tissues, biological replicates), lymph node (n = 8 tissues, biological replicates), tonsil (n = 9 tissues, biological replicates) and (**b**) epithelial tissues (*right*), including colon (n = 7 tissues, biological replicates), liver (n = 9 tissues, biological replicates), pancreas (n = 12 tissues, biological replicates), kidney (n = 8 tissues, biological replicates), lung (n = 8 tissues, biological replicates) and skin (n = 7 tissues, biological replicates). Representative images are shown. Data derived from: https://www.proteinatlas.org/ENSG00000168036-CTNNB1/tissue#expression_summary (**c-d**) β-catenin expression was assessed by immunohistochemistry (brown) and using hematoxylin as counterstain (blue) on tissue microarrays from (**c**) B-lymphoid malignancies, including mantle cell lymphoma (n = 12 tumors, biological replicates), follicular lymphoma (n = 24 tumors, biological replicates), DLBCL (n = 24 tumors, biological replicates) and Hodgkin’s lymphoma (n = 24 tumors, biological replicates) as well as (**d**) epithelial cancers, including colon cancer (n = 12 tumors, biological replicates), lung cancer (n = 12 tumors, biological replicates) and malignant melanoma (n = 3 tumors, biological replicates). (**e**) Western blot analysis of β-catenin expression in cytoplasmic fractions of epithelial cancers, including lung and colon cancer, malignant melanoma, as well as B-lymphoid malignancies, including B-ALL, diffuse large B-cell lymphoma (DLBCL), mantle cell lymphoma (MCL), Burkitt’s, Hodgkin’s disease (HD) and multiple myeloma cell lines. β-tubulin and TBP were used to verify purity of cytoplasmic fractions. Source data

Studying β-catenin protein levels by immunohistochemistry in normal lymphoid tissues (*n* = 30) in comparison to epithelial and mesenchymal tissues (*n* = 51) (Extended Data Fig. 2a,b)^34^ (https://www.proteinatlas.org/) as well as lung cancer (*n* = 15), colon cancer (*n* = 25), malignant melanoma (*n* = 5) in comparison to mantle cell lymphoma (MCL; *n* = 26), follicular lymphoma (*n* = 38), diffuse large B cell lymphoma (DLBCL; *n* = 35) and Hodgkin’s disease (HD; *n* = 44), revealed a previously unrecognized lack of β-catenin protein expression in normal and malignant B cells (Fig. 1a and Extended Data Fig. 2c,d). In stark contrast to solid tumor cell lines, nuclear β-catenin was barely detectable in B cell malignancies, while four of 22 B lymphoid cell lines expressed low levels of cytoplasmic β-catenin (Fig. 1b and Extended Data Fig. 2e). Studying publicly accessible datasets, including RNA sequencing (RNA-seq) and reverse-phase protein arrays (RPPAs)^35^ (https://depmap.org/), revealed an unexpected discordance between β-catenin mRNA and protein levels in B cell malignancies. β-catenin mRNA levels were similar in B cell malignancies and solid tumors. However, β-catenin protein (RPPA) was barely detectable in B-ALL and B cell lymphoma cells (Fig. 1c). To address this discordance, we engineered nine epithelial and ten B lymphoid cell lines with a dual-reporter construct for concurrent detection of β-catenin mRNA (mScarlet) and protein (GFP). As expected, epithelial cells showed a linear relationship between mRNA and protein levels. Compared to epithelial cells, B-ALL and B cell lymphoma cells showed ~32-fold lower protein-to-mRNA ratios (*P* = 2.2 × 10^−8^; Fig. 1d,e).

### β-catenin is poised for proteasomal degradation in B-ALL cells

**Extended Data Fig. 3:**
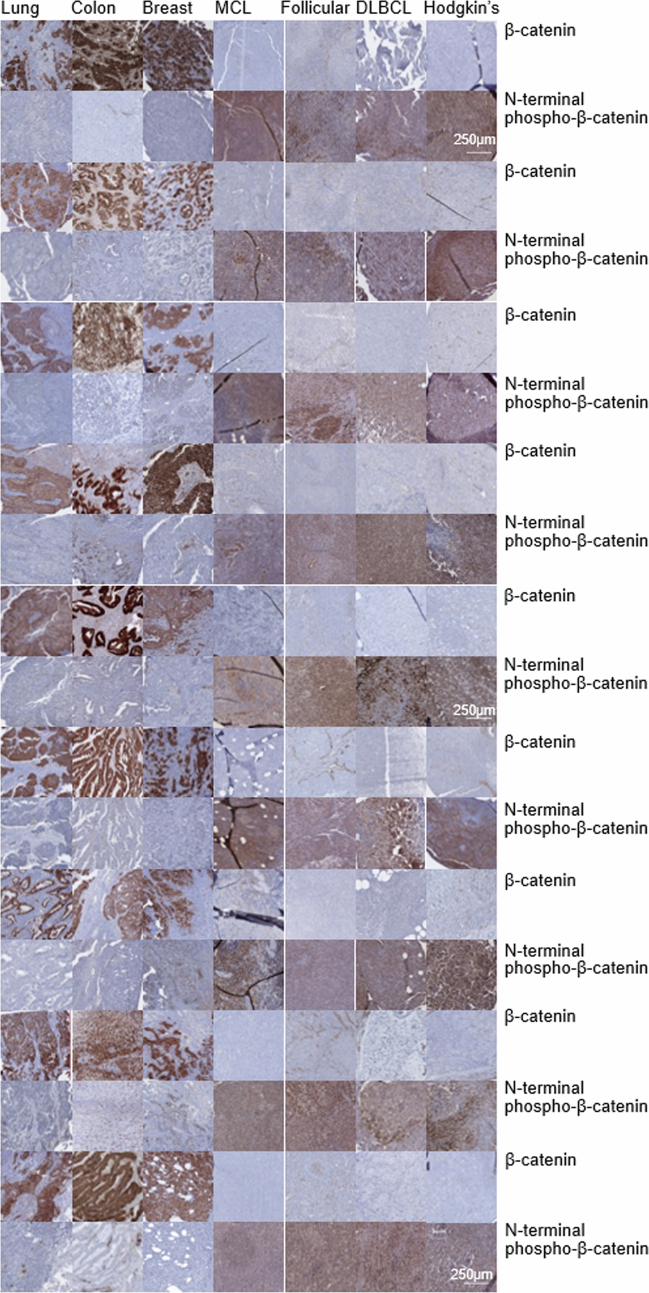
β-catenin is constitutively phosphorylated and poised for degradation in B-lymphoid cells. Non-phosphorylated β-catenin and β-catenin phosphorylated on N-terminal by GSK3B (pS37) were visualized by immunohistochemistry (brown, counterstain hematoxylin) on tissue microarrays from epithelial cancers, including colon cancer (n = 13 tumors, biological replicates), lung cancer (n = 41 tumors, biological replicates), breast cancer (n = 58 tumors, biological replicates), as well as B-lymphoid malignancies, including mantle cell lymphoma (n = 21 tumors, biological replicates), follicular lymphoma (n = 54 tumors, biological replicates), DLBCL (n = 47 tumors, biological replicates) and Hodgkin’s lymphoma (n = 20 tumors, biological replicates). Representative images are shown.

**Extended Data Fig. 4:**
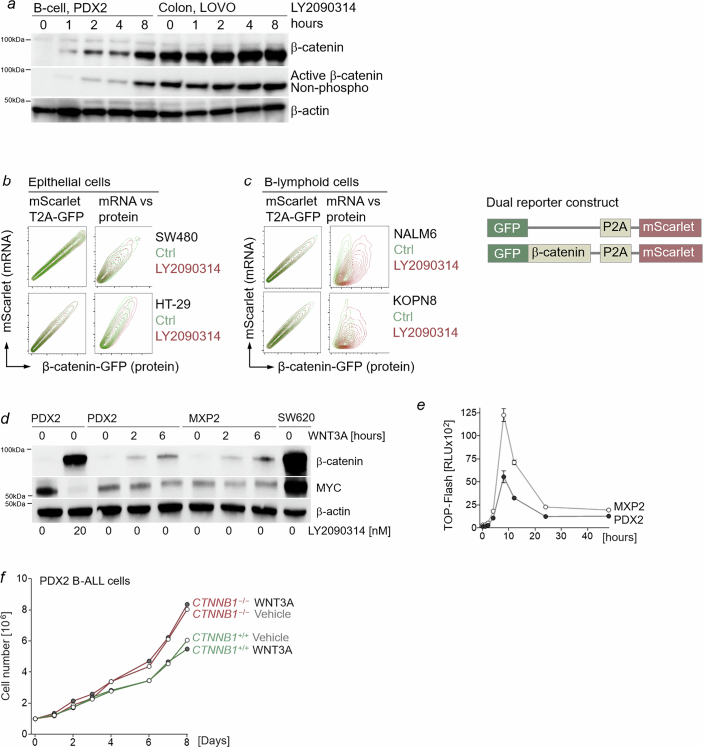
GSK3B-inhibition impairs β-catenin protein degradation. (**a**) Human B-ALL (PDX2) and colon cancer (LOVO) cells were treated with LY2090314 for 0-8 hours and Western blot was performed to measure total β-catenin, as well as active, non-phosphorylated β-catenin using β-actin as loading control (n = 3 independent experiments). (**b**-**c**) FACS plots are shown for dual reporter cells that were treated with the GSK3B-inhibitor LY2090314 (10 nM; red) or vehicle control (green) for 16 hours. Inhibition of β-catenin protein degradation by LY2090314 was compared in (**b**) human colon cancer (SW480, HT-29) cell lines and in (**c**) human B-ALL (NALM6, KOPN8) cell lines by measuring the changes in GFP- compared to mScarlet-signal (2 independent repeats). (**d**) β-catenin and Myc levels in B-ALL cells (PDX2, MXP2) following 0-6 h WNT3A (400 ng/ml) treatment in comparison to levels achieved following GSK3β inhibition (LY2090314, 20 nM) and colon cancer (SW620) (n = 2 independent experiments). (**e**) B-ALL xenografts (MXP2, PDX2) were stimulated with WNT3A conditioned media for indicated time points and activation of Wnt/β-catenin pathway was assessed by measurement of luminescence signal by TOP-Flash reporter. Data is representative of 2 independent experiments and shown as mean±s.d (n = 3 technical replicates). (**f**) Effect of WNT3a (grey circle) stimulation on growth of B-ALL cells (PDX2) that are wild type (CTNNB1 + /+, green line) and knock-out (CTNNB1-/-, red line) for β-catenin compared to vehicle control (white circle). Data shows mean values from 2 independent experiments each with n = 3 technical replicates. Source data

β-catenin protein degradation is initiated by N-terminal phosphorylation by GSK3β and CK1α (refs. ^3,4^), while nonphosphorylated β-catenin is active and stable^6^. Studying tissue sections and protein lysates with antibodies recognizing N-terminally phosphorylated, nonphosphorylated and total β-catenin revealed that β-catenin in solid tumors (*n* = 134) was mostly stable in the nonphosphorylated conformation (Fig. 1f,g and Extended Data Fig. 3). In contrast, B cell lymphomas (*n* = 142) only expressed N-terminally phosphorylated β-catenin, while stable nonphosphorylated β-catenin was not detectable (Fig. 1f,g and Extended Data Fig. 3). Treatment of patient-derived B-ALL xenograft (PDX) cells with the small-molecule GSK3β inhibitor LY2090314 for 8 h (10 nM) suppressed β-catenin degradation and induced accumulation of nonphosphorylated β-catenin (Extended Data Fig. 4a). Studying LY2090314 in B-ALL cells carrying the dual β-catenin mRNA (mScarlet) and protein (GFP) reporter showed that GSK3β inhibition induced a diagonal distribution between β-catenin mRNA and protein as in epithelial cells (Extended Data Fig. 4b,c). Treatment of B-ALL PDX cells with 400 ng ml^−1^ Wnt3A ligand resulted in only weak and transient β-catenin accumulation and failed to affect MYC expression or proliferation of B-ALL cells (Extended Data Fig. 4d–f) highlighting that high-efficiency β-catenin protein degradation in B cells limits Wnt3A-induced β-catenin accumulation.

### Lack of genetic lesions in the β-catenin protein degradation pathway in B cell malignancies

**Fig. 2:**
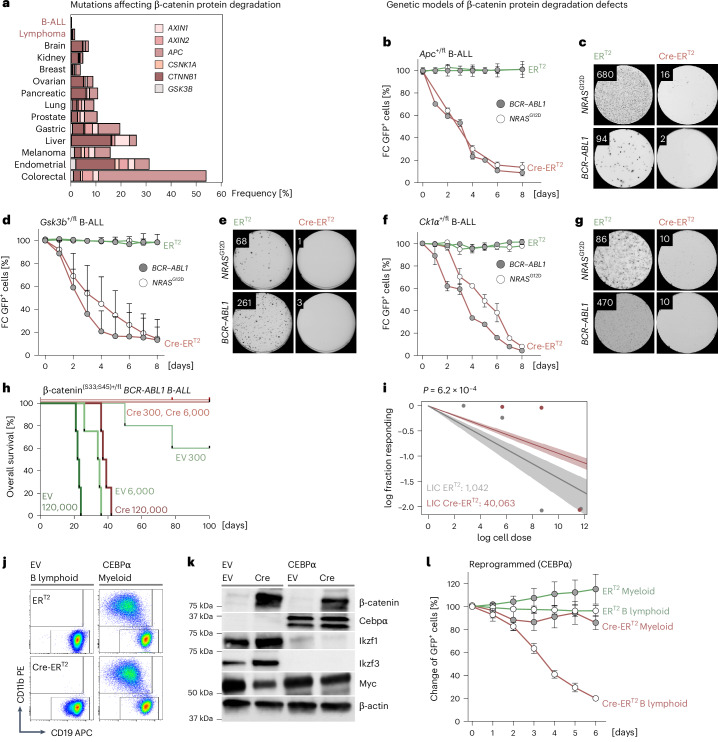
B cell malignancies are uniquely dependent on efficient β-catenin protein degradation. **a**, Frequencies of nonsynonymous coding mutations of β-catenin (*CTNNB1*; filtered for hotspot mutations of GSK3β and CK1α phosphorylation sites), *APC*, *AXIN1*, *AXIN2*, *CSNK1A* and *GSK3B* are shown for 14 types of cancer including B cell leukemia and lymphoma (*n* = 4,387 tumors, biological replicates) and solid tumors (*n* = 86,426 tumors, biological replicates). **b**,**c**, *Apc*^fl/+^ pre-B cells were transformed with *BCR*–*ABL1* or *NRAS*^G12D^ to establish B-ALL cell lines and subsequently transduced with vectors expressing 4-OHT-inducible Cre (Cre-ER^T2^) or EV control (ER^T2^) along with GFP. **b**, Changes in frequencies of GFP^+^ cells were studied by FACS for 0–8 days after 4-OHT addition. Representative data from two independent experiments are shown (*n* = 3 biological replicates). Data are presented as the mean ± s.d. **c**, *Apc*^fl/+^ B-ALL cells expressing Cre-ER^T2^ or ER^T2^ constructs (sorted for GFP^+^) were plated on methylcellulose 1 day after 4-OHT treatment and colonies were counted 14 days after plating. Colony numbers for cells (ER^T2^ versus Cre-ER^T2^) carrying *NRAS*^G12D^: 680 ± 61 versus 16 ± 8. Colony numbers for cells (ER^T2^ versus Cre-ER^T2^) carrying *BCR*–*ABL1*: 94 ± 22 versus 2 ± 2. Data are shown as the mean ± s.d. (*n* = 3 biological replicates, two independent experiments). **d**,**e**, *BCR*–*ABL1* or *NRAS*^G12D^ transformed *Gsk3b*^fl/+^ B-ALL cells were transduced with constructs expressing Cre-ER^T2^ or ER^T2^ and GFP. **d**, Increases or decreases in frequencies in GFP^+^ cells were studied by FACS for 0–8 days after 4-OHT addition. Data show the mean ± s.d. calculated from three independent experiments (*n* = 3 technical replicates). **e**, GFP^+^ B-ALL cells were plated for colony formation assays 1 day after 4-OHT treatment. Representative images from two independent experiments are shown for 14 days after plating (colony numbers for ER^T2^ versus Cre-ER^T2^ cells (mean ± s.d.) with *NRAS*^G12D^: 68 ± 16 versus 1 ± 1; colony numbers for cells (ER^T2^ versus Cre-ER^T2^) carrying *BCR*–*ABL1*: 261 ± 47 versus 3 ± 2) (*n* = 3 technical replicates). **f**,**g**, *BCR*–*ABL1* or *NRAS*^G12D^ transformed cells with monoallelic CK1α deletion (*Ck1a*^fl/+^; Cre-ER^T2^) and CK1α WT cells (*Ck1a*^fl/+^; ER^T2^). Changes in frequencies of GFP^+^ cells were measured by FACS for 8 days. Data are representative of two independent experiments (*n* = 3 biological replicates) and presented as the mean ± s.d. **g**, Representative images and average number of colonies 10 days after plating (two independent experiments). **h**,**i**, β-catenin^(S33^^;S45)+/fl^ B-ALL cells (*BCR*–*ABL1*) were transduced with vectors expressing GFP-tagged Cre-ER^T2^(Cre) or ER^T2^(EV). GFP^*+*^ cells were sorted by FACS and injected into sublethally irradiated NSG mice 2 days after 4-OHT treatment. ELDA was performed to assess effects of β-catenin accumulation on leukemia-initiation capacity (LIC) of B-ALL cells. **h**, Kaplan–Meier analysis performed for calculating survival in each group. Log-rank test for survival of 120,000 Cre versus 120,000 EV transplanted mice (*n* = 4 mice in each group, biological replicates), *P* = 0.0067. Log-rank test for 6,000 Cre versus 6,000 EV transplanted mice (n = 4 mice each group, biological replicates), *P* = 0.0067. Log-rank test for 300 Cre (*n* = 4 mice, biological replicates) versus 300 EV transplanted mice (*n* = 5 mice, biological replicates), *P* = 0.18. **i**, LIC was determined in B-ALL cells with β-catenin accumulation (1 in 40,063 cells) and control cells (1 in 1,042) using Chi-square test and 90% CI is depicted (*P* = 0.0006). (**j**,**k**) β-catenin^(S33-S45)+/fl^ B-ALL (*BCR*–*ABL1*) cells were transduced with doxycycline-inducible vectors expressing myeloid transcription factor CEBPα or EV and were subsequently transduced with GFP-tagged Cre-ER^T2^ or ER^T2^ vectors for excision of β-catenin GSK3β and CK1α phosphorylation sites. CEBPα-driven myeloid reprogramming was induced by addition of doxycycline. (**j**) Flow cytometry analysis to identify myeloid (CD11b^+^) and B lymphoid (CD19^+^) cells two days after doxycycline treatment. **k**, Western blot analysis to measure CEBPα, Ikzf1, Ikzf3 and Myc levels following β-catenin accumulation in B-ALL after CEBPα myeloid reprogramming (CEBPα) or EV conditions (*n* = 3 independent repeats). **l**, Changes in frequencies of GFP^+^ cells were monitored by FACS for 0–6 days after 4-OHT-mediated activation of Cre and accumulation of β-catenin. Data are shown as the mean ± s.d. from three independent experiments (*n* = 3 technical replicates). Source data

Loss-of-function lesions of GSK3β, APC, CK1α and other components of the β-catenin protein degradation machinery are common oncogenic drivers throughout the spectrum of cancer^17–20^. Studying deleterious β-catenin protein degradation pathway lesions (filtered for coding, nonsilent) in 90,812 cancer samples encompassing 13 types of solid tumors and B cell malignancies^36^, we found frequent mutations in solid tumors (*n* = 86,426), namely *APC* (14%), *AXIN1* (1.7%), *AXIN2* (1.5%), *GSK3B* (0.5%) and *CSNK1A1* (CK1α; 0.4%), as well as GSK3β and CK1α phosphorylation sites in β-catenin (4.7%) (Fig. 2a). In contrast, among 4,386 B cell malignancies, lesions in the β-catenin protein degradation pathway were significantly underrepresented (expected: 1,119, observed: 21, *χ*² test *P* = 1.2 × 10^−12^; Fig. 2a). These results suggest that, unlike other cancers, lesions of the β-catenin degradation pathway are not oncogenic drivers in B cell malignancies.

### B-ALL cells critically depend on GSK3β, CK1α and APC for efficient β-catenin protein degradation

**Extended Data Fig. 5:**
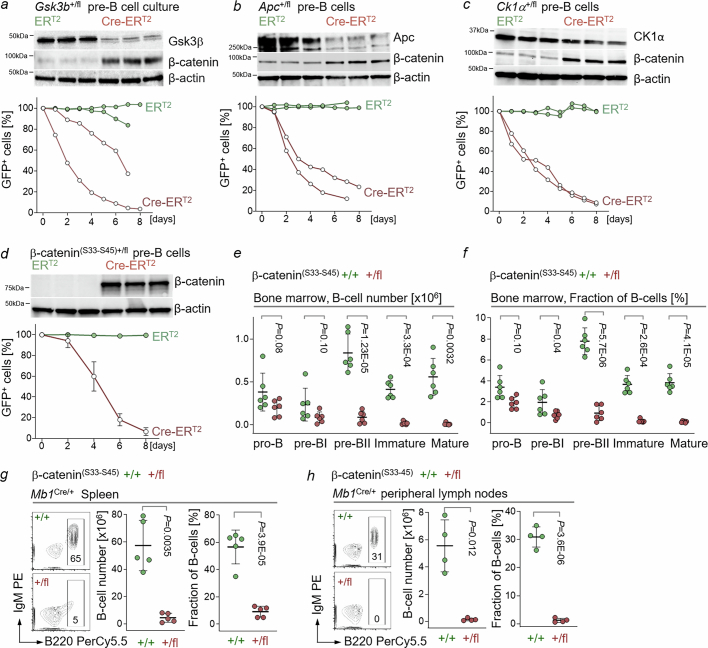
Genetic attenuation of β-catenin protein degradation induces acute cell death in pre-B cells in vitro and in vivo. (**a**) Pre-B cells from *Gsk3b*^fl/+^ mice were transduced with 4-OHT-inducible Cre-ER^T2^ or ER^T2^ along with GFP. Western blot analysis was performed to visualize *Gsk3b* deletion and β-catenin accumulation in GFP^+^ cells 2 days after 4-OHT mediated deletion of one allele of *Gsk3b*. Effects of *Gsk3b* haploinsufficiency and β-catenin accumulation were assessed in competitive growth assays monitoring changes in percentages of GFP^+^ cells for 0-8 days after 4-OHT treatment. Mean values from 2 independent experiments each with n = 2 technical replicates are shown. **(b)** *Apc*^fl/+^ pre-B cells were transduced with GFP-tagged Cre-ER^T2^ or ER^T2^ constructs, and Western blot analysis was performed in GFP^+^ cells to confirm Apc levels and accumulation of β-catenin 2 days after 4-OHT treatment. Following loss of one allele of APC, competitive fitness of pre-B cells was assayed by measuring changes in percentages of GFP^+^ cells. Data presents mean values from 2 independent experiments with each n = 3 technical replicates. **(c)** *Ck1a*^fl/+^ pre-B cells were transduced with Cre-ER^T2^ or ER^T2^ and GFP. Western blot analysis was performed for Ck1α, β-catenin and β-actin 2 days after 4-OHT mediated CK1α deletion. Growth kinetics of cells with monoallelic deletion of Ck1α (Cre-ER^T2^) was compared to cells with wild type Ck1α (ER^T2^) by flow cytometry of changes in GFP^+^ cells 0-8 days post 4-OHT treatment. Data shows mean values from 2 independent experiments each with n = 2 technical replicates. **(d)** Pre-B cells from β-catenin^(S33-S45)+/fl^ mice expressing Cre-ER^T2^ or ER^T2^ along with GFP were treated with 4-OHT to induce Cre-mediated excision of GSK3B-phosphorylation sites. Accumulation of β-catenin was confirmed by Western blot. Removal of exon-3 results in a shorter form of β-catenin. Competitive fitness of pre-B cells was assessed by measuring the frequencies of GFP^+^ cells by FACS. Data are presented as the mean±s.d of 3 independent experiments each with n = 3 technical replicates. **(e-h)** β-catenin^(S33-S45)+/fl^ mice were crossed with *Mb1*^Cre/+^ mice for B-cell-specific excision of GSK3B-phosphorylation sites and B-cell development in the bone marrow and peripheral lymphoid organs was studied by FACS. **(e)** Absolute numbers (**f**) percentages of B-cell precursor subsets in the bone marrow of the mice are shown for *Mb1*^Cre/+^ β-catenin^(S33-S45)+/fl^ (red, n = 6 mice, biological replicates) and *Mb1*^Cre/+^ β-catenin^(S33-S45)+/+^ mice (green, n = 6 mice, biological replicates). Bone marrow B-cell precursors were distinguished as pro-B cells (CD43^+^ B220^low^ IgM^−^ BP1^−^), pre-BI cells (CD43^+^ B220^low^ IgM^−^ BP1^+^), pre-BII cells (CD43^−^ B220^low^, IgM^−^), immature B cells (CD43^−^ B220^low^ IgM^+^) and mature B cells (CD43^−^ B220^high^ IgM^+^). **(g)** Representative FACS plots and absolute numbers and frequencies of B220^+^ B-cells in spleens of *Mb1*^Cre/+^ β-catenin^(S33-S45)+/fl^ (red, n = 5 mice, biological replicates) and *Mb1*^Cre/+^ β-catenin^(S33-S45)+/+^ (green, n = 5 mice, biological replicates) mice are shown. **(h)** Representative FACS plots, absolute numbers, and fractions [%] of B-cells in the peripheral lymph nodes of *Mb1*^Cre/+^ β-catenin^(S33-S45)+/fl^ (red, n = 4 mice, biological replicates) and *Mb1*^Cre/+^ β-catenin^(S33-S45)+/+^ (green, n = 4 mice, biological replicates) mice are shown. Throughout (**e-h**) two tailed unpaired t-tests were used to calculate P values and data are presented as mean±s.d. Source data

To address the role of central components of the β-catenin protein degradation pathway in B-ALL cells, we developed genetic loss-of-function models of APC (*Apc*^+/fl^), GSK3β (*Gsk3b*^+/fl^) and CK1α (*Csnk1a*^+/fl^). To model haploinsufficiency of APC, GSK3β and CK1α in B-ALL cells, we transduced B cell precursors isolated from *Apc*^+/fl^, *Gsk3b*^+/fl^ and *Csnk1a*^+/fl^ mice with *BCR*–*ABL1* or *NRAS*^G12D^ oncogenes and Cre (Cre-ER^T2^) or empty vector (EV) controls (ER^T2^) for tamoxifen-inducible deletion. Induced haploinsufficiency of APC, GSK3β and CK1α resulted in acute cell death and loss of colony formation in *BCR*–*ABL1*-driven and *NRAS*^G12D^-driven B-ALL cells (CD19^+^CD43^+^IgM^−^), corroborating dependency on efficient β-catenin degradation (Fig. 2b–g and Supplementary Fig. 1a). Likewise, in normal pre-B cells, deletion of one allele of either APC, GSK3β or CK1α resulted in cell death and elimination of pre-B cells from cell culture (Extended Data Fig. 5a–c), highlighting the importance of intact β-catenin degradation for B cell survival. N-terminal β-catenin S33, S37, T41 and S45 residues are phosphorylated by the concerted activity of GSK3β, CK1α and APC^3,4^. To determine whether the dependency of B-ALL cells on GSK3β, CK1α and APC (Fig. 2b–g) is mechanistically linked to phosphorylation of S33, S37, T41 and S45 residues, we studied a genetic model based on excision of S33, S37, T41 and S45 residues^2^, leaving other functions of GSK3β, CK1α and APC intact. Excision of N-terminal phosphorylation sites in β-catenin^(S33;S45)+/fl^ pre-B cells induced β-catenin accumulation and acute cell death (Extended Data Fig. 5d). In β-catenin^(S33;S45)+/fl^ mice that were crossed with a B cell-specific *Mb1*-Cre deleter strain, B lymphopoiesis was arrested at pre-BCR^+^ stages in the bone marrow, spleen and lymph nodes (Extended Data Fig. 5e–h and Supplementary Fig. 1b). Together, these results show that B cell development depends on the activity of GSK3β, CK1α and APC, N-terminal phosphorylation and degradation of β-catenin.

### B-ALL leukemia initiation depends on high efficiency of β-catenin protein degradation

To determine whether B-ALL cells depend on effective β-catenin protein degradation in vivo, we transduced β-catenin^(S33;S45)+/fl^ B-ALL cells with tamoxifen-inducible Cre (Cre-ER^T2^) or EV controls (ER^T2^). Compared to EV controls, activation of Cre substantially delayed leukemogenesis and prolonged survival of transplant recipient mice (Fig. 2h). Defective β-catenin protein degradation also impaired leukemia initiation in vivo as shown in extreme limiting dilution assays (ELDAs) based on 300, 6,000 and 120,000 injected β-catenin^(S33;S45)+/fl^ B-ALL cells carrying tamoxifen-inducible Cre-ER^T2^ or ER^T2^ EVs. ELDA analysis revealed a 38-fold reduction of leukemia-initiating cells compared to controls (*P* = 6.2 × 10^−4^; Fig. 2h,i).

### Dependency on β-catenin protein degradation is predicated on B cell identity

To assess whether this vulnerability reflects B cell-intrinsic transcriptional programs that determine B cell identity, we reprogrammed β-catenin^(S33;S45)+/fl^ B-ALL cells into the myeloid lineage through inducible expression of the myeloid transcription factor CEBPα. Then, 2 days after doxycycline-induced expression of CEBPα, B-ALL cells expressed the myeloid cell antigen CD11b (Mac1) and downregulated CD19 and the B cell transcription factors Ikzf1 (Ikaros) and Ikzf3 (Aiolos; Fig. 2j,k). Activation of Cre in β-catenin^(S33;S45)+/fl^ B-ALL cells subverted β-catenin protein degradation, competitive fitness and MYC expression. However, CEBPα-mediated B cell-to-myeloid reprogramming rendered leukemia cells resistant to β-catenin accumulation and restored clonal fitness and MYC expression (Fig. 2k,l). These findings suggest the diverging roles of β-catenin in myeloid and B cells and raise the possibility that lineage-specific transcription factors predicate the unique dependency of B lymphoid cells on β-catenin protein degradation.

### β-catenin protein degradation is required to maintain MYC expression in B lymphoid cells

**Fig. 3:**
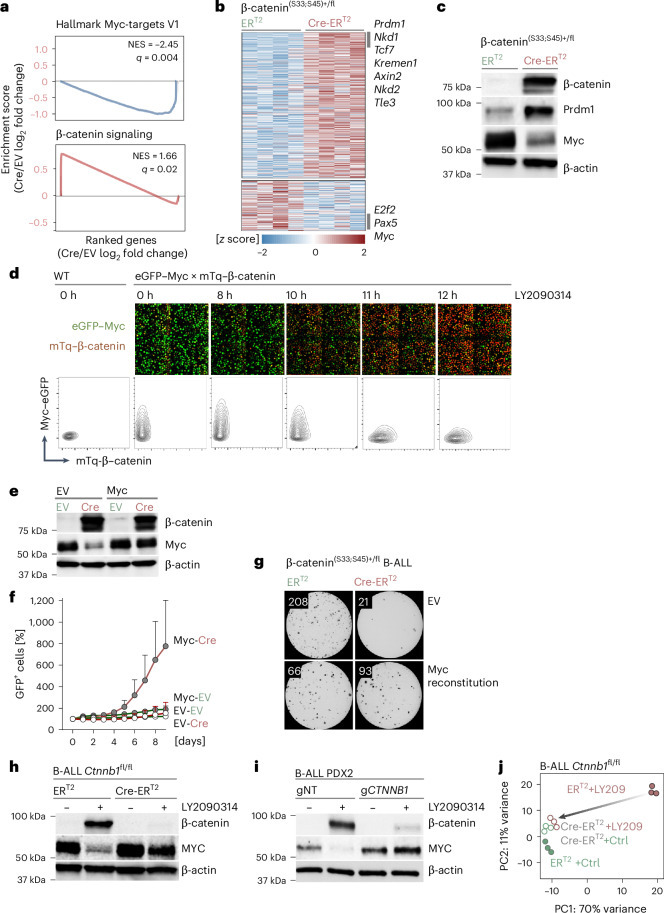
Impaired β-catenin protein degradation in B cells results in transcriptional repression of MYC. **a**,**b**, *BCR*–*ABL1* transformed β-catenin^(S33;S45)+/fl^ B-ALL cells expressing Cre-ER^T2^ or ER^T2^ vectors were treated with 4-OHT to excise GSK3β and CK1α phosphorylation sites on β-catenin. Gene expression changes were studied by RNA-seq (GSE305472) 1 day after 4-OHT-mediated β-catenin stabilization (*n* = 4 cell lines, biological replicates). **a**, GSEA identified depletion of Myc target genes and enrichment of Wnt–β-catenin signaling as top-ranking gene sets following β-catenin accumulation. **b**, Genes that were upregulated (*n* = 354) or downregulated (*n* = 119) upon β-catenin stabilization are shown as a heat map. Genes with the most prominent upregulation included *Prdm1* and negative regulators of Wnt–β-catenin signaling such as *Axin2*, *Kremen1*, *Nkd1*, *Nkd2* and *Tle3*, while *Myc* and *Pax5* were amongst downregulated genes. **c**, Western blot analysis to detect changes in Prdm1 and Myc levels following β-catenin stabilization in β-catenin^(S33;S45)+/fl^ B-ALL cells using β-actin as loading control (*n* = 2 independent experiments). **d**, Pre-B cells from transgenic mice expressing a dual Myc-β-catenin reporter were transformed with *BCR*–*ABL1* to develop a B-ALL cell line. Dual-reporter cells with expression of an N-terminal eGFP–Myc fusion and N-terminal mTurquoise–β-catenin fusion protein (Myc–eGFP × mTq–β-catenin) were treated with 10 nM LY2090314 for 12 h. Changes in Myc–eGFP and mTq–β-catenin protein expression were assessed by measuring eGFP and mTurquoise signal, respectively. Representative confocal microscopy images (top) for eGFP–Myc (green) and mTq–β-catenin (red) are shown for 0–12 h of LY2090314 treatment. FACS plots (bottom) for measuring mTq–β-catenin (*x* axis) and eGFP–Myc (*y* axis) levels are presented for WT and Myc–β-catenin reporter cells for the indicated time points of LY2090314 treatment. FACS and time-lapse imaging experiments were performed two times each. **e**–**g**, *BCR*–*ABL1* transformed β-catenin^(S33;S45)+/fl^ B-ALL cells expressing Cre-ER^T2^ or ER^T2^ (puromycin selected) were transduced with GFP-tagged Myc or EV. **e**, Confirmation of β-catenin and Myc expression in FACS-sorted GFP^+^ cells by western blot 3 days after 4-OHT treatment. **f**, FACS analysis to monitor enrichment or depletion of GFP^+^ cells (Myc versus EV) upon β-catenin activation. Data are presented as the mean ± s.d. calculated from three independent experiments (*n* = 3 technical replicates). **g**, Colony formation abilities of cells expressing Myc or EV were assessed 2 days after 4-OHT-induced β-catenin accumulation. Data shown are representative of two independent experiments (*n* = 3 technical replicates). Colony numbers for each condition: ER^T2^ EV, 208 ± 32; Cre-ER^T2^ EV, 21 ± 7; ER^T2^ Myc, 66 ± 36, Cre-ER^T2^ Myc, 93 ± 36 (mean ± s.d). **h**, *BCR*–*ABL1* transformed B-ALL cells from *Ctnnb1*^fl/fl^ mice were transduced with vectors expressing Cre-ER^T2^ or ER^T2^ and treated with 4-OHT for 2 days to induce β-catenin deletion. Western blot was performed to analyze β-catenin and Myc levels 16 h after LY2090314 treatment (40 nM; middle) in β-catenin knockout (*Ctnnb1*^−/−^, Cre-ER^T2^) or WT (*Ctnnb1*^+/+^, ER^T2^) B-ALL cells (*n* = 3 independent experiments). **i**, Human B-ALL cells (PDX2) were edited with HDR to generate cells with homozygous or heterozygous deletion of *CTNNB1*. β-catenin-deficient (gCTNNB1) or WT (gNT) PDX2 cells were treated with LY2090314 (20 nM) for 16 h. Western blot was performed to analyze β-catenin and MYC levels following GSK3β inhibition (*n* = 2 independent experiments). **j**, β-catenin knockout (Cre-ER^T2^) or WT (ER^T2^) B-ALL cells were treated with LY2090314 for 16 h and RNA-seq was performed to characterize transcriptomic changes (GSE245287). Principal component (PC) analysis of gene expression changes in *Ctnnb1*^fl/fl^ B-ALL cells upon β-catenin deletion (Cre-ER^T2^) compared to WT (ER^T2^) cells (*n* = 3 mice, biological replicates) in the presence or absence of GSK3β inhibition. Although GSK3β inhibition in β-catenin competent cells induced major changes in gene expression (ER^T2^ + LY2090314 versus ER^T2^), this effect was reversed by β-catenin deletion (Cre-ER^T2^ + LY2090314). Source data

To determine the underlying mechanism of B lymphoid-specific dependency on β-catenin protein degradation, we examined gene expression changes upon activation of Cre in β-catenin^(S33;S45)+/fl^ B-ALL cells. RNA-seq analysis revealed enrichment for two principal gene sets, namely activation of Wnt–β-catenin signaling and suppression of Myc targets (Fig. 3a). The most prominent gene expression changes, repression of Myc and upregulation of Prdm1, a central negative regulator of Myc, were confirmed by Western blot (Fig. 3b,c). In addition, a set of β-catenin targets and feedback regulators (Nkd1, Nkd2, Tcf7, Kremen1, Axin2 and Tle3) were strongly increased upon β-catenin stabilization. β-catenin-mediated repression of Myc in B-ALL cells contradicted previous studies in epithelial cells that identified *MYC* as a classical Wnt target and strongly activated by TCF7L2–β-catenin complexes^9,10^. To directly visualize the unexpected dependency of MYC expression on efficient β-catenin protein degradation in B-ALL cells, we generated a dual-reporter knock-in model expressing mTq–β-catenin and eGFP–Myc fusions. B-ALL cells generated from dual-reporter knock-in mice expressed high levels of Myc at baseline and lacked β-catenin protein expression (Fig. 3d). Treatment with the GSK3β small-molecule inhibitor LY2090314 resulted in a dramatic shift after 10 h of treatment with de novo expression of mTq:β-catenin and subsequent loss of eGFP:Myc signal (Fig. 3d and Supplementary Video 1). Reconstitution of MYC expression rescued the deleterious effects of β-catenin accumulation and restored the colony formation and competitive fitness of B-ALL cells (Fig. 3e–g). Analogous to genetic deletion of GSK3β (*Gsk3b*^+/fl^; Fig. 2d,e and Extended Data Fig. 5a), treatment with the GSK3β inhibitor LY2090314 reduced β-catenin protein degradation, resulting in repression of MYC in murine and human B-ALL cells (Fig. 3h,i). Importantly, genetic deletion of β-catenin was sufficient to relieve MYC suppression in response to GSK3β inhibition in mouse and human B-ALL cells (Fig. 3h,i). These results corroborate that the expression of MYC in B-ALL cells critically relies on GSK3β-mediated β-catenin degradation. Beyond suppression of MYC, GSK3β-inhibitor-induced gene expression changes broadly depend on β-catenin accumulation. Principal component analyses visualized that genetic deletion of β-catenin alone reversed the effects of LY2090314, indicating that gene expression changes, most notably repression of MYC, largely reflect subversion of GSK3β-mediated β-catenin protein degradation (Fig. 3j).

### β-catenin forms repressive complexes with Ikaros factors and NuRD components in B-ALL cells

**Fig. 4:**
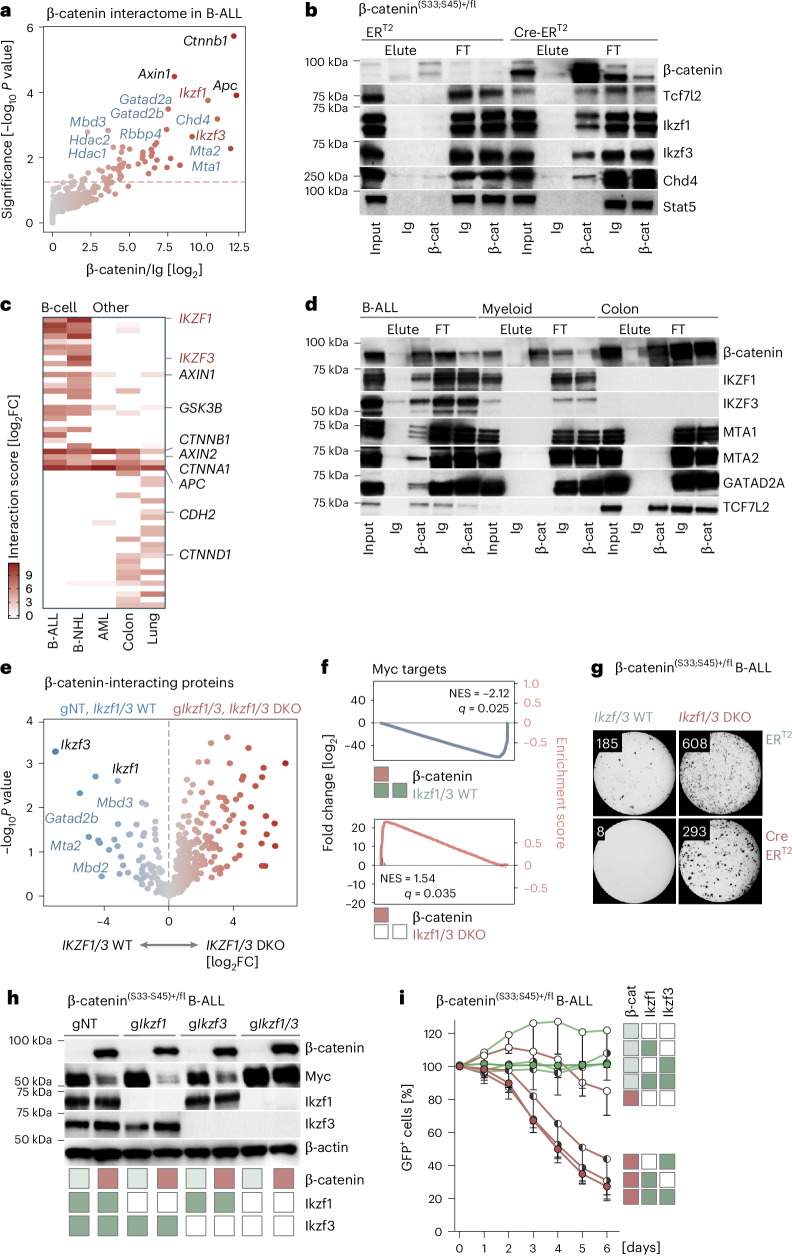
β-catenin forms repressive complexes with B lymphoid transcription factors Ikzf1 and Ikzf3. **a**, Proteins bound to β-catenin in *BCR*–*ABL1* transformed B-ALL cells from β-catenin^(S33;S45)+/fl^ mice were enriched by co-IP, identified by mass spectrometry and plotted on the basis of statistical significance and log_2_ fold enrichment over IgG background control (*n* = 4 technical replicates). Proteins with the most prominent binding to β-catenin included Ikaros factors Ikzf1 and Ikzf3 (red) and members of the repressive NuRD complex Chd4, Gatad2a, Gatad2b, Mta1, Mta2, Mdb3, Rbbp4, Hdac1 and Hdac2 (blue). Linear modeling and empirical Bayes testing used for differentially enriched proteins. **b**, β-catenin-interacting proteins were validated by co-IP and western blot in whole-cell lysates (input), proteins bound (elute) and flowthrough (FT) to isotype control or antibodies against β-catenin, using Stat5 as a negative control (*n* = 2 independent experiments). **c**, Human B-ALL (MXP2), B cell lymphoma (JEKO1), AML (MOLM13), colon cancer (SW480) and lung cancer (H446) cell lines were engineered to express doxycycline-inducible degradation resistant form of β-catenin. Co-IP experiments with antibodies to β-catenin or control Ig were performed 1 day after doxycycline treatment. Eluted proteins were analyzed by mass spectrometry. The heat map shows the interaction score (*y* axis, log_2_ fold change) of β-catenin-binding proteins normalized to Ig control in B-ALL, MCL, AML, colon cancer and lung cancer cell lines. FC, fold change. **d**, Whole-cell lysates (input), proteins bound (elute) and FT with β-catenin antibodies or control Ig were analyzed by western blotting to study interactions between β-catenin and Ikaros factors (IKZF1 and IKZF3), NuRD complex components (MTA1, MTA2 and GATAD2A) and TCF7L2 in B-ALL (PDX2), myeloid leukemia (JURL-MK1) and colon cancer (SW620) cells 16 h after pharmacological β-catenin stabilization (LY2090314, 20 nM) (*n* = 2 independent experiments). **e**–**i**, *BCR*–*ABL1* transformed β-catenin^(S33;S45)+/fl^ B-ALL cells were gene-edited with gRNAs targeting Ikaros factors (*Ikzf1* and *Ikzf3*; individually or both) or gNT. Deletion of Ikaros factors was confirmed by western blot in clonal cell lines established from single cells. Multiple clones were studied for each genotype. β-catenin^(S33;S45)+/fl^ B-ALL cells were transduced with 4-OHT-inducible GFP-tagged Cre-ER^T2^ or ER^T2^ constructs. **e**, Changes in β-catenin interactomes in B-ALL cells upon Ikaros factor deletion (g*Ikzf1/3*) were analyzed by co-IP and mass spectrometry. Proteins bound to β-catenin were plotted on the basis of significance (−log_10_ *P* value; *y* axis) and abundance change (log_2_ fold enrichment; *x* axis) compared to B-ALL cells without deletion of Ikaros factors (gNT; *n* = 3 technical replicates). DKO, double knockout. **f**, GSEA plots show the depletion of MYC target genes upon β-catenin accumulation in the presence of Ikaros factors (top), which were reversed by the deletion of Ikaros factors (bottom) (GSE196767). **g**, Colony-forming assays for B-ALL cells with (*Ikzf1/3* double knockout) and without (*Ikzf1/3* WT) Ikaros factor deletion were performed upon β-catenin stabilization (Cre-ER^T2^) compared to baseline β-catenin expression (ER^T2^). Representative images and mean colony numbers from three independent experiments each with three technical replicates are shown 10 days after plating. **h**, Western blot analysis to study changes in Myc levels in single-cell-derived B-ALL clones with deletion of *Ikzf1* and/or *Ikzf3* (white box) in comparison to clones WT for both Ikaros factors (dark-green box) following β-catenin stabilization (red box) compared to baseline conditions (light-green box) (*n* = 3 independent experiments). **i**, The competitive fitness of B-ALL clones was assessed for baseline β-catenin levels (ER^T2^, light-green box) or following β-catenin accumulation (Cre-ER^T2^, red box) in Ikzf1 and Ikzf3 WT cells (gNT, dark-green box) or upon deletion of *Ikzf1* and/or *Ikzf3* (g*Ikzf1*, g*Ikzf3*; white box). Data are presented as the mean ± s.d. of three independent experiments (*n* = 3 technical replicates). Source data

**Extended Data Fig. 6:**
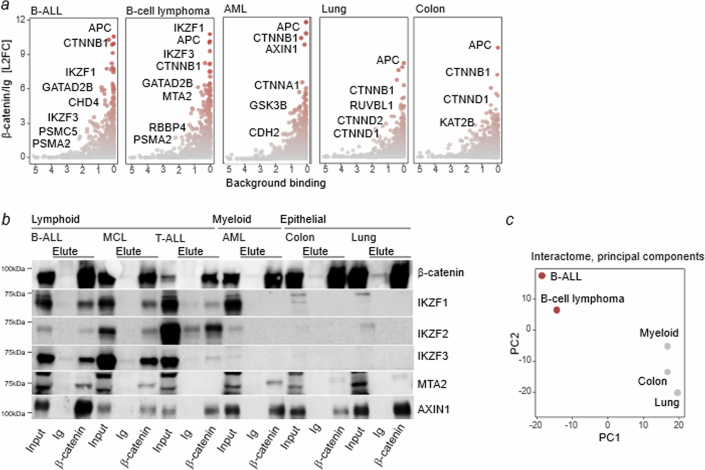
β-catenin forms repressive complexes with Ikaros and NuRD factors in B-cells. (**a-c)** Human B-ALL (MXP2), B-cell lymphoma (JEKO), AML (MOLM13), colon cancer (SW480) and lung cancer (H446) cell lines were engineered with β-catenin mutations impairing GSK3B and CK1a-mediated phosphorylation. **(a)** Proteins bound to β-catenin were identified by mass-spectrometry following Co-IP with antibodies against β-catenin or control Ig. β-catenin binding proteins in each cell type were plotted as a function of background binding (x-axis, non-specific binding defined by CRAPOME database) and log_2_-fold enrichment over control Ig (y-axis). **(b)** Western blot was performed to validate β-catenin-binding of Ikaros factors IKZF1, IKZF2, IKZF3 and NuRD complex component MTA2, as well as AXIN1 (positive control) using input lysates and elutes from the Co-IP experiments with antibodies against β-catenin or control Ig (n = 2 biological replicates). (**c**) Principal component analysis corroborated B-lymphoid-specific (B-ALL, B-cell lymphoma) β-catenin-interactomes that were clustered together along PC1 axis and separated from myeloid, colon and lung epithelial cells. Source data

To elucidate how β-catenin accumulation repressed MYC in B cells, we identified β-catenin-interacting proteins by coimmunoprecipitation (Co-IP) and mass spectrometry. In addition to known β-catenin interaction partners (Apc, Axin1 and Gsk3β), the proteins with the highest enrichment included B lymphoid Ikaros transcription factors (Ikzf1 and Ikzf3) (Fig. 4a). Ikaros factors function as B cell-specific repressors to maintain B cell identity and recruit repressive NuRD components for transcriptional repression^37–41^. In addition to Ikzf1 and Ikzf3, we identified multiple NuRD complex components (Chd4, Mta1, Mta2, Gatad2a, Gatad2b, Rbbp4, Mbd3, Hdac1 and Hdac2) (Fig. 4a) among β-catenin-interacting proteins. Prominent interactions between β-catenin and Ikzf1, Ikzf3 and the NuRD component Chd4 were validated by co-IP and western blot (Fig. 4b). To examine lineage-specific composition of β-catenin-interacting protein complexes, we identified β-catenin-bound proteins by co-IP and mass spectrometry in human B lymphoid (B-ALL and B cell lymphoma), myeloid (AML) and epithelial (colon and lung cancer) cells. Confirming results in murine B-ALL, Ikaros factors IKZF1 and IKZF3 were among the most prominent β-catenin interaction partners in human B-ALL and B cell lymphoma (Fig. 4c and Extended Data Fig. 6a). Co-IP and western blot experiments confirmed B lymphoid-selective interactions between β-catenin and IKZF1, IKZF3, MTA1, MTA2 and GATAD2A as opposed to colon cancer cells with preferential TCF7L2–β-catenin interactions (Fig. 4d and Extended Data Fig. 6b). Principal component analysis corroborated B lymphoid-specific β-catenin interactomes that were clustered together along the first principal component axis and separated from myeloid, colon and lung epithelial cells (Extended Data Fig. 6c).

### Repressive β-catenin–NuRD complexes depend on B lymphoid Ikaros factors

To assess whether Ikaros factors are required for the recruitment of repressive NuRD components and their interactions with β-catenin, we deleted both Ikzf1 and Ikzf3 in murine B-ALL cells and repeated the co-IP and mass spectrometry identification of β-catenin-interacting proteins. Deletion of *Ikzf1/3* in B-ALL cells (*Ikzf1/3* double knockout) was achieved by electroporation-based delivery of Cas9 ribonucleoproteins (RNPs) containing Cas9 and guide RNAs (gRNAs) (g*Ikzf1/Ikzf3*) or nontargeting control (gNT). Deletion of Ikaros factors resulted in the loss of interactions between β-catenin and the NuRD complex components Mta2, Mbd2, Mbd3 and Gatad2b (Fig. 4e), suggesting a central role of Ikaros factors in assembling repressive β-catenin–NuRD complexes. To elucidate the contribution of Ikaros factors to β-catenin-mediated transcriptional repression, we compared gene expression (RNA-seq) in *Ikzf1/3* wild-type (WT) and *Ikzf1/3* double-knockout B-ALL cells. Deletion of both Ikaros factors largely reversed β-catenin-mediated repression of Myc and Myc targets (Fig. 4f), restored Myc expression and Myc-dependent transcriptional programs and rescued colony formation and cell survival in competitive cell culture assays (Fig. 4g–i). Deletion of *IKZF1* is a frequent lesion in B-ALL^42^, whereas *IKZF3* mutations are detected in mature B cell malignancies^43^. However, these lesions are monoallelic and cases with combined defects of *IKZF1* and *IKZF3* have not been reported to date. For this reason, we assessed whether deletion of one single Ikaros factor could reverse β-catenin-induced deleterious effects in B-ALL cells. Unlike *Ikzf1/3* double knockout, deletion of one Ikaros factor had no notable effect on β-catenin-induced repression of MYC and cell death in B-ALL cells (Fig. 4h,i). This result suggests that one single Ikaros factor is required and sufficient for β-catenin-dependent repression of MYC and induction of cell death.

### Ikaros–β-catenin complexes mediate transcriptional repression at MYC superenhancer regions

**Fig. 5:**
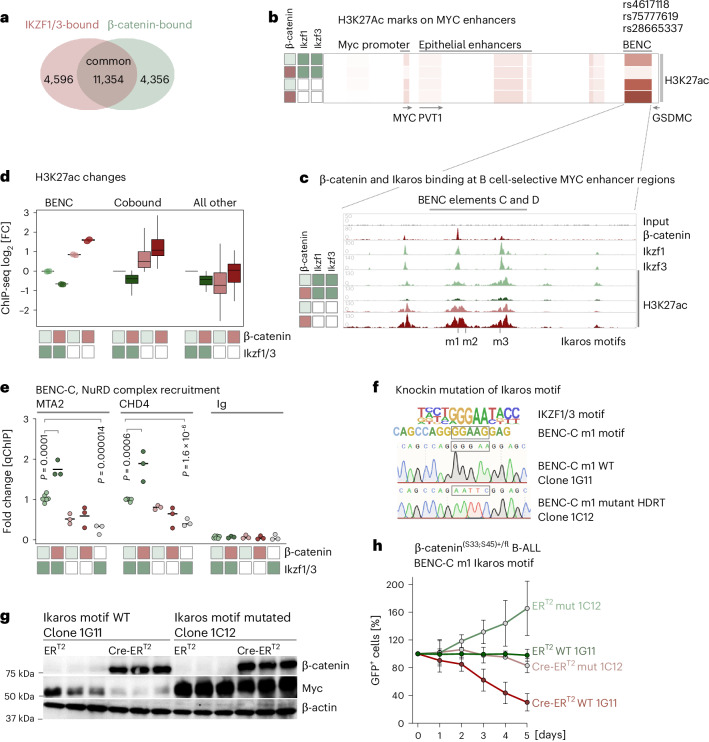
Identification of an Ikaros motif in the MYC BENC enhancer region required for β-catenin-mediated repression of MYC. **a**–**e**, *BCR*–*ABL1* transformed β-catenin^(S33;S45)+/fl^ B-ALL cells were edited with g*Ikzf1/3* or gNT. Clonal cell lines that are WT or knockout for *Ikzf1/3* were generated and transduced with 4-OHT-inducible Cre-ER^T2^ or ER^T2^ vectors. Transcriptional targets of Ikzf1, Ikzf3 and β-catenin and changes in histone mark H3K27ac were studied by ChIP-seq in *Ikzf1/3* WT (dark-green box) or *Ikzf1/3* knockout (white box) cells in the presence (red box) or absence (light-green box) of β-catenin stabilization (GSE196745). **a**, Venn diagram showing the number of regions bound by β-catenin only (4,356), Ikaros factors only (4,596) or both (11,354). Of 15,710 β-catenin peaks, 11,354 (72.2%) were also bound by Ikaros factors. **b**, ChIP-seq analysis of histone mark H3K27ac is shown as a heat map for the *Myc* locus, including upstream *Myc* promoter regions and long-range transcriptional enhancers of *Myc* in B-ALL cells from β-catenin^(S33;S45)+/fl^ mice. H3K27ac distribution marking active enhancer regions shows that most of the H3K27ac enhancer activity is concentrated in BENC regions in B-ALL cells. In humans, single-nucleotide polymorphisms in this region are associated with increased risk for B-ALL (rs4617118, rs75777619 and rs28665337). **c**, Enrichment of β-catenin, Ikzf1 and Ikzf3 binding to BENC elements C and D and changes in H3K27ac upon induction of β-catenin (red box) in B-ALL cells with (white box) or without (dark-green box) deletion of Ikaros factors compared to baseline conditions (light-green box). Identification of Ikaros-binding motifs in the BENC-C (m1 and m2) and BENC-D (m3) elements. **d**, Quantification of H3K27ac signals (mean value of *n* = 2 cell lines, technical replicates) by ChIP-seq at BENC enhancer regions, other regions with binding of both Ikaros factors and β-catenin (cobound) and all other regions. H3K27ac ChIP was performed with (red box) and without (light-green box) β-catenin accumulation and in the presence (white box) or absence (dark-green box) of Ikaros factor deletion. Box plots display boxes at the first and third quartiles with a line at the median and whiskers from the minimum to maximum data points within 1.5× the interquartile range (BENC, *n* = 2; cobound, *n* = 1,043; other, *n* = 42 peaks). **e**, ChIP–qPCR analysis for recruitment of NuRD complex components (MTA2 and CHD4) to the BENC-C enhancer region in β-catenin^(S33;S45)+/fl^ B-ALL cells that are WT (dark-green box) or knockout (white box) for *Ikzf1/3* under baseline β-catenin (light-green box), following β-catenin stabilization (red box) or deletion of β-catenin (white box). Data represent a pool of six independent experiments. Mean values from independent experiments (*n* = 3 technical replicates) were used to compare changes in MTA and CDH4 binding upon β-catenin stabilization compared to baseline and β-catenin knockout condition compared to baseline (two-sided unpaired *t*-test). **f**, HDR-mediated editing of the BENC-C m1 motif to abrogate binding of Ikzf1 and Ikzf3 by mutating Ikaros core motif GGGAA and generation of an EcoR1 recognition sequence (GAATTC). Following EcoR1 digestion to confirm mutation of the BENC-C m1 motif, single-cell-derived clones were generated and clones carrying the BENC-C m1 motif mutation were identified by Sanger sequencing. **g**,**h**, Single-cell-derived clones from β-catenin^(S33;S45)+/fl^ B-ALL cells with WT or mutated BENC-C Ikaros m1 motifs were transduced with vectors expressing Cre-ER^T2^ or ER^T2^ along with GFP. **g**, GFP^+^ cells were sorted by FACS and western blot analysis was performed to characterize β-catenin and Myc protein levels 1 day after 4-OHT treatment in B-ALL cells carrying intact or mutated BENC-C Ikaros m1 motifs in the presence (Cre-ER^T2^) or absence (ER^T2^) of β-catenin stabilization. **h**, GFP^+^ cells with intact (WT 1G11) or mutated BENC-C Ikaros m1 motifs (mut 1C12) were mixed with cells with an intact BENC-C Ikaros m1 motif and the effects of β-catenin stabilization were measured in competitive cell culture experiments by flow cytometry. Data depict the means ± s.d. calculated from three independent experiments (*n* = 3 technical replicates). Source data

**Extended Data Fig. 7:**
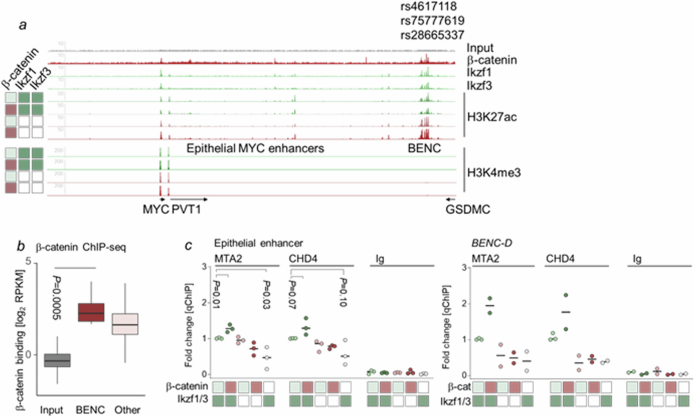
β-catenin and Ikaros factors mediate recruitment of NuRD complex at BENC enhancer. (**a**) ChIP-seq analysis for β-catenin, Ikaros factors Ikzf1 and Ikzf3, histone marks H3K27ac and H3K4me3 shown for the *Myc* locus, including upstream *Myc* promoter regions and long-range transcriptional enhancers of *Myc* in B-ALL cells from β-catenin^(S33-S45)+/fl^ mice. Peak density plots show colocalization of β-catenin, Ikzf1 and Ikzf3 peaks and their concentration at BENC enhancer regions. Comparison of changes in H3K27ac active enhancer and H3K4me3 active promoter marks following accumulation of β-catenin (red boxes) or β-catenin baseline (light green boxes), upon Ikaros factor deletion (empty boxes) or Ikaros baseline (dark green boxes) conditions. Ikaros factors and β-catenin show marked enrichment at BENC regions. While accumulation of β-catenin depleted H3K27ac marks at BENC enhancer regions, β-catenin had the opposite effect and increased BENC enhancer activity and H3K27ac signals when Ikaros factors (*Ikzf1* and *Ikzf3*) were deleted. (**b**) β-catenin binding (mean value of two technical replicates) to BENC enhancer region and other β-catenin targets was measured by ChIP-seq in β-catenin^(S33-S45)+/fl^ B-ALL cells (Ikzf1/3 WT) 1 day after β-catenin stabilization. β-catenin is strongly enriched at BENC enhancer (input n = 11863, Other n = 11857, BENC n = 6 peaks; two-sided unpaired t-test *P* = 0.0005). Boxplots display boxes at first and third quartile with line at median and whiskers from minimum to maximum datapoints within 1.5x interquartile range. (**c**) ChIP-qPCR was performed for NuRD complex components (MTA2 and CHD4) at the epithelial Myc enhancer regions as well as lymphoid BENC *Myc* enhancer region (BENC-D). For each region, MTA2 and CHD4 ChIP were performed for B-ALL cells with induced β-catenin accumulation (red boxes), β-catenin deletion (empty boxes) or intact β-catenin (light green boxes), as well as deletion of Ikaros factors (empty boxes) or intact Ikaros factors (dark green boxes). Data shown represents mean values from 3 independent experiments (n = 3 technical replicates). Two-sided unpaired t-test was used to calculate P values. Source data

To determine how Ikaros factors and β-catenin interact at the chromatin level, we performed β-catenin and Ikzf1/3 chromatin IP and sequencing (ChIP-seq) analyses to study changes of β-catenin-binding and Ikaros-binding peaks upon deletion of Ikaros and accumulation of β-catenin. Highlighting the dominant role of Ikaros factors in controlling β-catenin functions in B cells, ChIP-seq analyses showed that nearly 75% of β-catenin peaks were shared with Ikaros (Fig. 5a). In most cell types, Myc superenhancer regions are found in a proximal cluster of peaks (50 kb to 450 kb downstream of *Myc*) that overlaps with the *Pvt1* gene^44,45^. Studying H3K27ac signals across multiple *MYC* superenhancer clusters in B-ALL cells revealed that most of the enhancer activity was concentrated in a different region, located 1.7 Mb downstream of Myc (Fig. 5b and Extended Data Fig. 7a). This region was previously identified as the hematopoietic-specific blood enhancer cluster (BENC) region^46^. Ikaros and β-catenin strongly bound to element C of the BENC region (Fig. 5c and Extended Data Fig. 7a,b). In the presence of Ikaros factors, β-catenin accumulation suppressed H3K27ac signals at BENC-C regions. However, upon Ikaros deletion, β-catenin accumulation had the opposite effect and greatly increased H3K27ac signals at BENC-C enhancer regions and other enhancers that were cobound by both β-catenin and Ikaros factors (Fig. 5b–d and Extended Data Fig. 7a). Mirroring β-catenin-mediated repression at MYC BENC enhancer regions, accumulation of β-catenin strongly increased recruitment of repressive NuRD complex components MTA2 and CHD4 (Fig. 5e). Consistent with Ikaros-dependent formation of repressive β-catenin–NuRD complexes (Fig. 4e), deletion of Ikaros factors abrogated recruitment of NuRD factors to BENC-C (Fig. 5e and Extended Data Fig. 7c) and restored H3K27ac marks for enhancer activity (Fig. 5b–d and Extended Data Fig. 7a). While Ikaros–β-catenin complexes suppressed MYC expression in B-ALL cells, these observations suggest that deletion of *Ikzf1/3* releases β-catenin from transcriptional repression and restores transcriptional activation of MYC.

### Ikaros and TCF7 factors compete for binding to β-catenin

**Extended Data Fig. 8:**
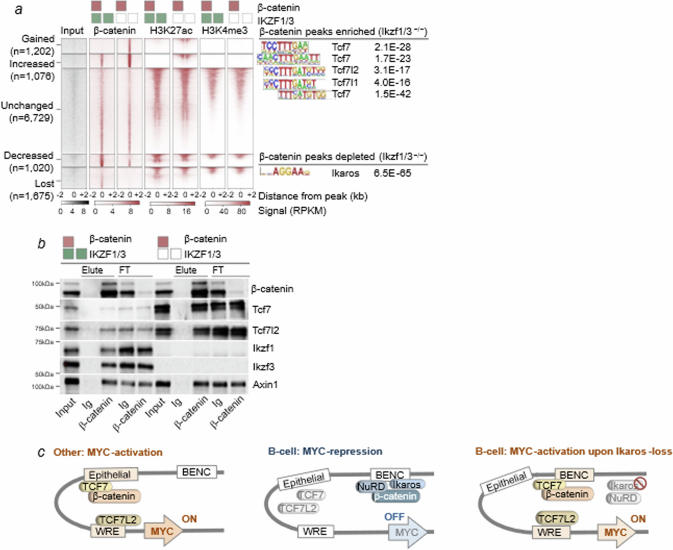
Ikaros factors compete with TCF family transcription factors for binding to β-catenin. **a**) Changes of β-catenin peaks as well as H3K4me3 and H3K27ac histone marks were assessed in the presence (green boxes) and absence of Ikaros factors (empty boxes) and inducible accumulation of β-catenin (red boxes). Heatmaps to illustrate genome-wide distribution of β-catenin peaks, H3K27ac and H3K4me3 histone marks. Ikzf1 and Ikzf3 deletion enabled binding of β-catenin to inactive enhancers and their subsequent activation (gained H3K27ac). Upon loss of Ikzf1 and Ikzf3 regions that have decreased (n = 1,020) or lost (n = 1,675) β-catenin binding are strongly enriched for Ikaros motifs (P = 6.5E-65). Deletion of Ikaros factors resulted in redistribution of β-catenin to canonical Tcf7 (P = 1.0E-42), Tcf7l2 (P = 1.0E-17) and Tcf7l1 (P = 1.0E-16) motifs and depletion from Ikaros motif (P = 6.5E-65), Fisher’s Exact test. **b**) Changes in β-catenin interactomes in mouse B-ALL cells upon Ikaros factor deletion (gIkzf1/3) were analyzed by co-IP and Western blot analysis in whole cell lysates (input), proteins bound (elute) and flow through (FT) after Co-IP with antibodies against β-catenin or control antibodies. Co-IP was performed under conditions of β-catenin accumulation (red box) and in the presence of Ikaros factor deletion (empty box) or Ikaros baseline levels (dark green box). Deletion of Ikaros factors enabled binding of β-catenin to Tcf7 and Tcf7l2 (n = 2 biological replicates). (**c**) Scenario of transcription factor complexes with β-catenin in B-lymphoid vs other cell types: TCF factors pair with β-catenin for transcriptional activation of Myc at Wnt responsive elements (WRE) and epithelial enhancer regions (left). In B-cells, Ikaros factors outcompete TCF to bind to β-catenin. Ikaros factors and β-catenin recruit of repressive NuRD complexes to B-lymphoid BENC enhancer regions of Myc, resulting in transcriptional repression of Myc (middle). Loss of Ikaros factors enables interactions between β-catenin and TCF factors to restore transcriptional activation of Myc (right). Source data

Accumulation of β-catenin did not affect Ikaros binding and ~90% of Ikaros peaks remained unchanged. In contrast, loss of Ikaros factors affected about half of all β-catenin targets and generated 1,202 new β-catenin-binding peaks, while 1,675 β-catenin peaks were lost (Extended Data Fig. 8a). When β-catenin protein degradation was subverted in the presence of Ikaros factors, new β-catenin-binding peaks were mostly devoid of H3K27ac marks for active enhancers. However, upon Ikaros deletion, β-catenin peaks were enriched for H3K27ac binding, suggesting increased enhancer activity at these sites after Ikaros deletion (Extended Data Fig. 8a). This shift suggests that Ikaros deletion released β-catenin from repressive complexes to form activating complexes with other transcription factors. For instance, Ikaros factors would not only promote repressive β-catenin–NuRD complexes but also oppose canonical interactions between β-catenin and TCF7 factors. Motif enrichment analyses revealed that Ikaros factor deletion restored targeting of β-catenin to canonical TCF7, TCL7L1 and TCF7L2 motifs at the expense of Ikaros motifs (Extended Data Fig. 8a). These results suggest that Ikaros factors sequester β-catenin away from transcriptional activation at canonical TCF7 sites and redirect β-catenin to Ikaros-binding sites for transcriptional repression in concert with NuRD factors. Consistent with this scenario, co-IP and western blot analyses (Extended Data Fig. 8b) showed that Ikaros factor deletion restored canonical interactions between β-catenin and TCF7-family transcription factors (Tcf7, Tcf7l1 and Tcf7l2) (Extended Data Fig. 8a–c).

### Identification and functional validation of an Ikaros motif required for MYC repression

The MYC superenhancer BENC-C region at 8q24.21 harbors three prominent risk alleles that predispose to B-ALL (rs4617118, rs75777619 and rs28665337) (Fig. 5b, Extended Data Fig. 7a)^47–49^. Combining Ikzf1 and Ikzf3 ChIP-seq and sequence analysis of the MYC superenhancer BENC-C region identified three Ikaros-binding motifs (m1–m3) within a window of 100 bp of notable Ikzf1/3 ChIP-seq peaks, including m1 with an exact match to the Ikaros core motif GGGAA (Fig. 5c,f). To assess the functional importance of the Ikaros m1 motif within the BENC-C region, we engineered knock-in alleles to mutate Ikaros m1 to an EcoRI site. After Homology-directed repair (HDR)-based knock-in of mutant BENC-C Ikaros motifs in β-catenin^(S33;S45)+/fl^ B-ALL cells, leukemia cells were plated for colony formation. Colonies carrying biallelic knock-in mutations of the Ikaros motif were selected on the basis of EcoRI digestion and confirmed by Sanger sequencing (Fig. 5f). Upon induction of Cre in β-catenin^(S33;S45)+/fl^ B-ALL cells, BENC-C WT knock-in B-ALL clones rapidly lost Myc expression and underwent cell death (Fig. 5g,h). In contrast, B-ALL clones carrying BENC-C knock-in alleles with mutant Ikaros motif expressed Myc at higher baseline levels and were resistant to β-catenin accumulation. Despite defective β-catenin degradation, the Myc levels, cell viability and competitive fitness of B-ALL clones carrying the mutant Ikaros m1 motif remained intact (Fig. 5g,h). These findings underscore that β-catenin-induced repression of Myc depends on its interactions with Ikaros factors, particularly one Ikaros-binding motif within the *Myc* BENC-C enhancer region.

### B cell-selective activity of GSK3β inhibitors to subvert β-catenin protein degradation

**Fig. 6:**
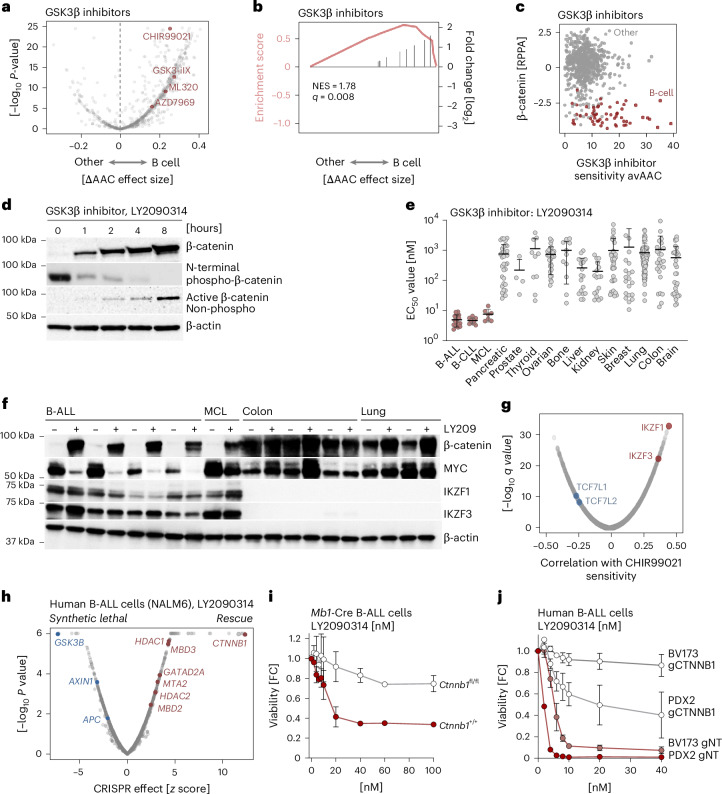
Small-molecule inhibition of β-catenin protein degradation induces B cell-selective cell death*.* **a**, Meta-analysis of cell-type-specific toxicities across three compound screens (CTD2, GDSC1 and GDSC2). The ΔAAC compares drug sensitivity in B cell lines (B-ALL, MCL and DLBCL) to solid tumor cell lines using the Wilcoxon effect size test. Inhibitors of GSK3β (AZD7969, GSK3iIX, CHIR99021 and ML320) show increased sensitivity in B lymphoid compared to solid tumors. **b**, Compound set enrichment analysis for GSK3β inhibitors was ranked by differential ΔAAC effect sizes as shown in **a** and demonstrated enrichment for B cell-selective effects (*q* = 0.008, NES = 1.78). **c**, Sensitivity to GSK3β inhibitors (ΔAAC; *x* axis) and β-catenin protein levels as measured by RPPA (CCLE2019; *y* axis) were plotted for human B lymphoid (*n* = 76 cell lines, biological replicates; red circles) and solid (*n* = 646 cell lines, biological replicates; gray circles) tumor cell lines. **d**, Human B-ALL (PDX2) cells were treated for 0–8 h with 10 nM LY2090314. Changes in total β-catenin, N-terminal phosphorylated β-catenin (S33, S37 and T41) and active β-catenin (nonphosphorylated) levels were analyzed by western blot. The exposure time for total and nonphosphorylated β-catenin was 185 s, whereas the exposure time for phosphorylated β-catenin (S33, S37, T41) was 1,199 s (*n* = 2 independent repeats). **e**, Drug responses to GSK3β inhibitor LY2090314 (EC_50_ values, nM) were calculated in B cell tumors (B-ALL, CLL and MCL; *n* = 34 cell lines, biological replicates; red circles), by measuring luminescence on day 3 and fitting of three-parameter log-logistic dose–response curves and comparing to epithelial cancer cell lines (*n* = 343 cell lines, biological replicates; Prism drug-repurposing screen). Data are presented as the mean ± s.d. **f**, B-ALL (MXP2, LAX2, BLQ5 and IAH8R), MCL (Z138), colon cancer (SW480, SW620 and LOVO) and lung cancer (H446 and H82) cell lines were treated with the GSK3β small-molecule inhibitor LY2090314 (20 nM) for 1 day. β-catenin, MYC, IKZF1 and IKZF3 protein levels were assessed by western blot, using β-actin as a loading control (*n* = 3 independent experiments). **g**, Computational analyses of correlations between gene expression (biomarker) and sensitivity to the GSK3β inhibitor CHIR99021 in B lymphoid and epithelial tumor cell lines are shown as a volcano plot with positive and negative correlation coefficients (*x* axis) and statistical significance (−log_10_ *q* value; *y* axis). B cell-specific expression of IKZF1 and IKZF3 was strongly correlated with high sensitivity to CHIR99021, while epithelial-specific TCF7L1 and TCF7L2 expression correlated with CHIR99021 resistance. **h**, To comprehensively identify mechanistic targets of GSK3β inhibition in human B-ALL cells, we performed a chemogenomic CRISPR screen. NALM6 B-ALL cells bearing an integrated inducible Cas9 expression cassette were transduced with the genome-wide knockout EKO sgRNA library (278,754 sgRNAs, targeting 22,956 genes, with 12 sgRNAs per gene and negative controls)^50^ and treated with LY2090314 at 3.5 nM to induce partial GSK3β inhibition for 8 days. Context-dependent chemogenomic interaction scores and *P* values were calculated using a modified version of the RANKS algorithm^50^, which uses sgRNAs targeting similarly essential genes as controls to distinguish condition-specific chemogenomic interactions from nonspecific fitness and essentiality phenotypes. **i**, Pre-B cells from β-catenin WT (Mb1-Cre × *Ctnnb1*^+/+^) and knockout (Mb1-Cre × *Ctnnb1*^fl/fl^) were transformed with *BCR*–*ABL1* to establish B-ALL cell lines. LY2090314 sensitivity was evaluated in β-catenin WT (Mb1-Cre × *Ctnnb1*^+/+^) and knockout (Mb1-Cre × *Ctnnb1*^fl/fl^) B-ALL cells by measuring luminescence 3 days after LY2090314 treatment (0–100 nM). Changes in viability were calculated by normalizing luminescence signals from treated cells to baseline values of untreated cells. Data are presented as the mean ± s.d. of three independent experiments (*n* = 3 technical replicates). **j**, β-catenin deletion was introduced in human B-ALL cell lines (BV173) and PDX (PDX2) cells by CRISPR–Cas9 using g*CTNNB1* or gNT. Human B-ALL cells that are WT (gNT) or knockout for β-catenin (g*CTNNB1*) were treated with LY2090314 and viability changes were assessed by measuring luminescence 3 days after treatment. Data are presented as the mean ± s.d. of three independent experiments (*n* = 3 technical replicates). Source data

**Extended Data Fig. 9:**
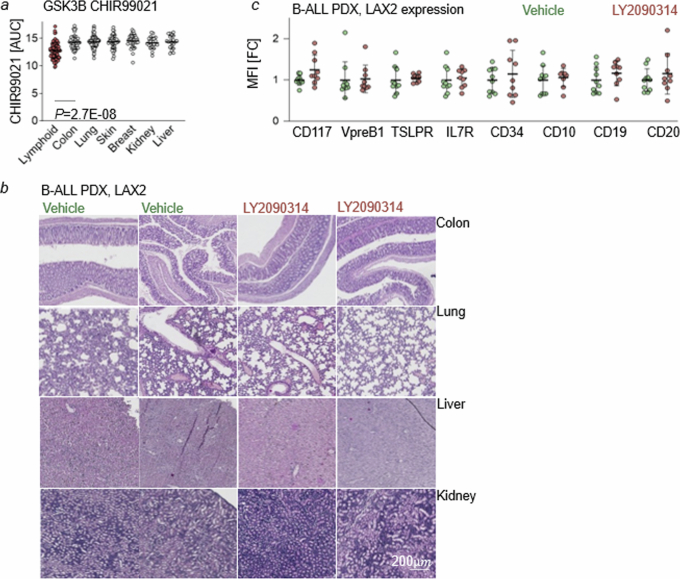
Repurposing clinically approved inhibitors targeting β-catenin degradation. (**a**) Drug responses (AUC) to the GSK3β-inhibitor (CHIR99021) were plotted for lymphoid (n = 98 cell lines, biological replicates) and solid (n = 298 cell lines, biological replicates) tumors (CTD2 screen). Data are represented as mean±s.d and two-sided unpaired t-test was used to calculate significance. (**b**) To assess whether LY2090314 induced any changes in epithelial tissues, histopathological evaluation was performed in colon, lung, liver and kidneys of the mice that received vehicle control or LY2090314 (n = 4 biological replicates/group) and representative images of H&E staining are shown. (**c**) To characterize phenotypic changes induced by LY2090314 treatment, B-ALL cells harvested from LY2090314 (red, n = 9 mice, biological replicates), or vehicle (green, n = 10 mice, biological replicates) treated mice were analyzed by FACS for surface expression of CD20, CD19, CD10, CD34, IL7R, TSLPR, VPREB1 and CD117. Fold-changes in mean fluorescence intensities (MFI [FC], y-axis) following LY2090314 treatment compared vehicle control are shown. No major differences were observed (two-sided unpaired t-test). Data are presented as mean±s.d. Source data

As B lymphoid cells were uniquely dependent on high-efficiency β-catenin protein degradation, we tested the hypothesis that small-molecule inhibitors of this pathway would induce cell death in a B cell-selective manner. We analyzed public drug sensitivity data (DepMap; https://depmap.org/)^35^ based on 4,518 compounds and 172 B lymphoid (32 B-ALL, 140 B cell lymphoma) and 1,787 nonlymphoid (colon, lung, skin, breast, kidney, liver and glioma) cell lines. Comparing B lymphoid to other cell lines, we plotted the differential area above the dose–response curve (ΔAAC) for all compounds on the basis of Wilcoxon effect size and significance (Fig. 6a). Along with known B cell-selective drugs (for example, ibrutinib and dexamethasone), the compounds with the most B cell-selective profiles included GSK3β inhibitors (Fig. 6a and Extended Data Fig. 9a). Moreover, compound set enrichment analyses confirmed strong B cell-selective activity for GSK3β inhibitors (normalized enrichment score (NES) = 1.78, *q* = 0.008; Fig. 6b). Sensitivity to GSK3β inhibitors was inversely correlated with β-catenin protein levels (RPPA, CCLE2019, *n* = 646; Fig. 6c). In agreement with this correlation, the GSK3β inhibitor LY2090314 not only exerted B cell-selective activity in DepMap datasets (https://depmap.org/)^35^ but also rapidly suppressed N-terminal β-catenin phosphorylation and protein degradation after 1 h (Fig. 6d). A combined analysis of responses to LY2090314 based on 343 epithelial and 42 B cell tumor cell lines (Prism drug-repurposing secondary screen; https://depmap.org/)^35^ revealed that B cell malignancies, including B-ALL (176-fold lower half-maximal effective concentration (EC_50_) values than solid tumors), MCL (147-fold) and CLL (218-fold) were substantially more sensitive than solid tumors (Fig. 6e). Comparing the effect of LY2090314 on β-catenin and MYC protein levels by western blot in B-ALL, MCL, colon cancer and lung cancer cell lines revealed that responses to LY2090314 were predicated on the expression of B lymphoid Ikaros factors (Fig. 6f). This was consistent with computational analyses of gene expression biomarkers for responses to the GSK3β inhibitor CHIR99021 (https://depmap.org/)^35^ CTD2 screen). This analysis identified expression of IKZF1 and IKZF3 as the top-ranking positive correlation with sensitivity to CHIR99021, whereas TCF7L1 and TCF7L2 levels were negatively correlated (Fig. 6g).

### Chemogenomic screens identified β-catenin degradation as a mechanistic target of GSK3β inhibitors

To comprehensively identify mechanistic targets of GSK3β inhibition in human B-ALL cells, we performed chemogenomic CRISPR screens in human B-ALL cells (NALM6) bearing an integrated inducible Cas9 expression cassette. NALM6 cells were transduced with the genome-wide knockout EKO single gRNA (sgRNA) library (278,754 sgRNAs, targeting 22,956 genes, with 12 sgRNAs per gene and negative controls)^50^ and treated with LY2090314 at 3.5 nM to induce partial GSK3β inhibition for 8 days. Context-dependent chemogenomic interaction scores were calculated using a modified version of the RANKS algorithm^50^, which uses sgRNAs targeting similarly essential genes as controls to distinguish condition-specific chemogenomic interactions from nonspecific fitness and essentiality phenotypes. sgRNAs targeting *CTNNB1* (β-catenin) had the single most prominent rescue effect in protecting B-ALL cells against LY2090314-mediated GSK3β-inhibition (Fig. 6h). Likewise, deletion of repressive NuRD complex components, including HDAC1, HDAC2, MBD3, GATAD2A and MTA2, provided a strong selective advantage. CRISPR sgRNAs targeting individual Ikaros factors did not have notable effects, presumably because concurrent deletion of *IKZF1/3* was required to rescue cell death and MYC repression (Fig. 4h,i). The chemogenomic CRISPR screen also revealed synthetic lethal interactions, including deletion of *AXIN1*, *APC* and most prominently *GSK3B* (Fig. 6h). To independently validate β-catenin protein degradation as central mechanistic target of GSK3β inhibitors, we compared the effect of LY2090314 in the presence and absence of β-catenin deletion. Cre-mediated deletion of β-catenin in murine β-catenin^fl/fl^ B-ALL cells prevented repression of MYC (Fig. 3h) and conferred near-complete resistance to LY2090314 (Fig. 6i). Likewise, CRISPR-mediated deletion of β-catenin in human B-ALL cell lines (BV173) and B-ALL PDX (PDX2) prevented suppression of MYC (Fig. 3i) and resulted in near-complete resistance to LY2090314 (Fig. 6j).

### Preclinical testing of GSK3β inhibitors for refractory B lymphoid malignancies

**Fig. 7:**
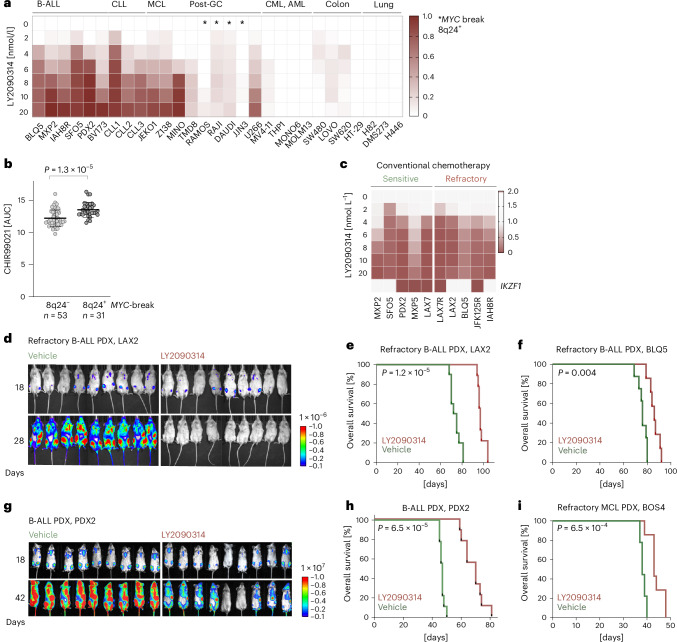
Rationale for repurposing of GSK3β inhibitors for refractory B lymphoid malignancies. **a**, Sensitivity to the GSK3β inhibitor LY2090314 was assessed in a panel of lymphoid (B-ALL, CLL, MCL and postgerminal center (post-GC) lymphoma; *n* = 18 cell lines, biological replicates), myeloid (CML and AML; *n* = 4 cell lines, biological replicates) and epithelial (colon and lung cancer; *n* = 7 cell lines, biological replicates) cancer cell lines by measuring luminescence signal 3 days after treatment. Growth-inhibitory effects are shown as a heat map. Asterisks denote B cell lines with *MYC* translocation. **b**, Drug responses (area under the curve) to the GSK3β inhibitor CHIR99021 (CTD2 screen) were plotted for 84 lymphoma and leukemia cell lines, comparing cell lines with (8q24^+^; *n* = 31 cell lines, biological replicates) and without (8q24^−^; *n* = 53 cell lines, biological replicates) *MYC* rearrangement as determined by fluorescence in situ hybridization or conventional cytogenetics. A two-sided unpaired *t*-test was used to compare groups. Data are presented as the mean ± s.d. **c**, Cell viability measurements following LY2090314 treatment were compared for human B-ALL samples from participants who responded to conventional chemotherapy (sensitive) and from participants with acquired chemoresistance (refractory). Boxes below the heat map indicate B-ALL samples with *IKZF1* lesions (deletion or mutation; red box). **d**,**e**, Luciferase-labeled LAX2 B-ALL cells (relapse sample) were injected into sublethally irradiated NSG mice. Mice were either treated with 12.5 mg kg^−1^ LY2090314 or vehicle control twice daily for four consecutive days per week (eight times a week) for 4 weeks. **d**, Leukemia burden was assessed by bioluminescence imaging on day 18 (top) and day 28 (bottom) following transplantation. **e**, Kaplan–Meier analysis of overall survival in each group (*P* = 1.2 × 10^−5^, calculated by log-rank test comparing vehicle (*n* = 10 mice, biological replicates) to LY2090314 (*n* = 9 mice, biological replicates)). **f**, Refractory B-ALL (BLQ5) cells were transplanted into sublethally irradiated NSG mice. Mice were either treated with 12.5 mg kg^−1^ LY2090314 or vehicle control twice daily for four consecutive days a week, for 4 weeks. Kaplan–Meier analysis was performed to calculate overall survival between groups treated with vehicle (*n* = 8 mice, biological replicates) and LY2090314 (*n* = 7 mice, biological replicates) (*P* = 0.004). **g**,**h**, Luciferase-labeled PDX2 cells (diagnosis sample) were injected into sublethally irradiated NSG mice, either treated with 12.5 mg kg^−1^ LY2090314 or vehicle control twice daily for 4 weeks. **g**, Leukemia burden was assessed by bioluminescence imaging on day 18 (top) and day 42 (bottom) after transplantation. **h**, Kaplan–Meier analysis of overall survival comparing vehicle-treated (*n* = 9 mice, biological replicates) to LY2090314-treated (*n* = 9 mice, biological replicates) group (*P* = 6.5 × 10^−5^, calculated by log-rank test). **i**, Refractory MCL cells (BOS4) were injected into sublethally irradiated NSG mice. Mice were either treated with 12.5 mg kg^−1^ LY2090314 or vehicle control twice daily, for four days a week, over a period of 3 weeks. Kaplan–Meier analysis was performed to calculate overall survival in vehicle-treated (*n* = 9 mice, biological replicates) versus LY2090314-treated (*n* = 7 mice, biological replicates) group (*P* = 6.5 × 10^−4^). Source data

To determine B cell selectivity of GSK3β inhibition, we compared LY2090314 sensitivity in B cell malignancies (*n* = 18) to myeloid and epithelial tumors (*n* = 12) (Fig. 7a). In contrast to myeloid, colon and lung cancer cells, LY2090314 elicited strong responses in B-ALL, CLL and MCL cells at low-nanomolar concentrations (EC_50_ = 4 nM) (Fig. 7a). DLBCL and other B cell lymphomas showed a bimodal distribution with three LY2090314-sensitive and four resistant cell lines carrying *MYC* translocations (RAMOS, RAJI, Daudi and JJN3) (Fig. 7a). To further examine uneven GSK3β inhibitor responses in this group, we studied CHIR99021 across 84 cell lines among lymphoid tumors, which confirmed two clusters of cell lines that substantially differed in sensitivity to GSK3β inhibition. The difference between the two groups tracked with *MYC* translocations. Lymphoid cell lines with *MYC* rearrangement (*n* = 31; 8q24^+^) were significantly less sensitive to GSK3β inhibition than cell lines without *MYC* translocation (n = 53, *P* = 1.3 × 10^−5^; Fig. 7b). Consistent with our finding that the MYC superenhancer region (BENC-C) is a critical target of repressive β-catenin–Ikaros complexes (Fig. 5), this observation suggests that chromosomal translocations in B cell lymphomas that remove MYC from its intrinsic control elements will confer resistance to GSK3β inhibition. As *IKZF1* deletions are common in human B-ALL^42^, we assessed the impact of *IKZF1* deletion on sensitivity to the GSK3β inhibitor LY2090314. Studying ten PDXs, including five with *IKZF1* deletion, we found no significant differences in responses to LY2090314 (Fig. 7c). In addition, whether B-ALL PDXs were derived from participants who previously responded to standard chemotherapy or were refractory or relapsed did not affect responses to LY2090314 (Fig. 7c). This result suggests that GSK3β inhibition is orthogonal to conventional mechanisms of drug resistance and may represent a vulnerability that is retained in relapsed B cell malignancies.

Five GSK3β inhibitors have achieved favorable safety and pharmacokinetic and pharmacodynamic (PK/PD) profiles in 20 clinical trials (Extended Data Fig. 1b)^28–33^. To assess the efficacy of GSK3β inhibitors in human B-ALL and MCL PDX models in vivo, we treated immunodeficient NSG mice bearing PDX from refractory B-ALL cells or MCL with LY2090314 as a single agent (Fig. 7d–i). Sublethally irradiated (2 Gy) NSG mice bearing refractory B-ALL and MCL PDX were injected intraperitoneally with LY2090314 or vehicle control. In our treatment experiment, LY2090314-treated mice did not show notable weight loss or any indication of organ toxicity in the colon, lung, liver and kidneys (Extended Data Fig. 9b). In addition, B-ALL cells in treated mice had no notable phenotypic changes (Extended Data Fig. 9c). Compared to vehicle-treated mice, LY2090314 reduced tumor burden and significantly extended overall survival in primary and refractory B-ALL PDX, as well as refractory MCL PDX (Fig. 7d–i).

## Discussion

A central finding of our study that B-ALL cells have evolved and critically depend on high-efficiency β-catenin protein degradation is seemingly in conflict with previous work suggesting a role of β-catenin as an oncogenic driver in B cell malignancies. In a common B-ALL subset, the *E2A*–*PBX1* fusion oncogene induced aberrant overexpression of WNT16 (ref. ^26^); however, subsequent work showed that WNT16 did not induce canonical β-catenin signaling^27^. Two studies examined the effects of Wnt3A and β-catenin accumulation on proliferation of B-ALL cells with conflicting results^24,25^. To reconcile the differences, we treated B-ALL PDX cells with 400 ng ml^−1^ Wnt3A ligand. Treatment with Wnt3A resulted in weak and transient β-catenin accumulation and failed to suppress MYC expression or proliferation of B-ALL cells (Extended Data Fig. 4d–f). While B-ALL cells are responsive to Wnt3A, these results suggest that high-efficiency β-catenin protein degradation in B cells limits the amount and duration of β-catenin accumulation.

Previous studies demonstrated that deletion of *FZD9* results in a defect in early B cell development^51^, whereas deletion of *FZD6* delays disease onset in a mouse model of CLL^52^. Given that Frizzled receptors are widely known as mediators of canonical Wnt signaling to stabilize β-catenin, these findings seem to contradict previous studies demonstrating that β-catenin is dispensable for normal B cell development^11–14^ and our work highlighting the dependency of B cells on β-catenin protein degradation. In addition to stabilizing β-catenin, Frizzled receptors can initiate β-catenin-independent, noncanonical Wnt signaling, which could explain the different outcomes of *FZD6* and *FZD9* deletion^51,52^ and β-catenin deletion^11–14^. Deletion of *FZD6* in CLL cells resulted in a 1.8-fold reduction in β-catenin protein levels (measured by flow cytometry)^52^. However, β-catenin protein levels in B cell malignancies were 32-fold lower compared to solid tumors as reported by our study (Fig. 1 and Extended Data Figs. 1 and 2), indicating that the small effect of *FZD6* deletion on β-catenin protein levels may not be functionally relevant.

The biological importance of high-efficiency β-catenin protein degradation is illustrated by genetic defects of this pathway. For instance, 5q− syndrome with heterozygous deletion and haploinsufficiency of *APC* (5q21) and CK1α (*CSNK1A*; 5q32)^53^ represents the most frequent myelodysplastic syndrome (MDS) and results in reduced efficiency of β-catenin protein degradation^54,55^. While 5q− MDS exhibits hyperplasia of myeloid and megakaryocytic lineages, persons suffer from anemia and defective B lymphopoiesis^53–55^. Our genetic experiments showed that haploinsufficiency of APC or CK1α caused accumulation of β-catenin and B cell depletion (Extended Data Fig. 5b,c).

Five GSK3β inhibitors previously achieved favorable safety and PK/PD profiles at micromolar plasma concentrations (*C*_max_). Despite potential concerns related to accumulation of β-catenin, even after 18 months of treatment at micromolar concentrations, only mild adverse effects, including diarrhea and lymphopenia, were observed^28–33^ (Extended Data Fig. 1b). Lymphopenia as a side effect is consistent with a rationale to repurpose GSK3β inhibition for B lymphoid malignancies and suggests that GSK3β inhibitors affect both normal and malignant B cells. Systemic B cell depletion as a side effect of treatment of B cell malignancies is generally well tolerated. For instance, antibodies to CD20 (for example, rituximab) are highly effective in the treatment of B cell lymphomas and induce profound B cell depletion^56^. Likewise, inhibitors of B cell signaling, including Bruton’s tyrosine kinase (for example, ibrutinib)^57^ are effective components of a therapeutic regimen for B cell lymphoma, induce B cell depletion and are well tolerated. In addition, cell therapeutics based on chimeric antigen receptor T cells against CD19 have revolutionized the treatment of persons with B-ALL and long-lasting B lymphopenia in these persons is considered an acceptable side effect^58^. GSK3β inhibitors have demonstrated safety in more than 20 clinical trials with low-micromolar plasma concentrations (Extended Data Fig. 1b). Importantly, our preclinical studies show that GSK3β small-molecule inhibitors are effective at low-nanomolar (~100-fold lower) concentrations in B cell malignancies. Together, these results support a rationale for repurposing GSK3β inhibitors to inhibit β-catenin protein degradation as a previously unrecognized strategy that is amenable to near-term evaluation in persons with refractory B cell malignancies.

## Methods

### Ethical approvals

Our research complies with all relevant ethical guidelines. Details of the committees and institutions that approved the study protocol can be found in each specific section.

### Participant samples and cell lines

Participant samples were obtained in compliance with the Institutional Review Boards of University of California, San Francisco, Yale University and the Dana-Farber Cancer Institute. Written informed consent was obtained from all participants. Leukemia and lymphoma cells (Supplementary Table 1) were cultured in RPMI-1640 (Gibco) with GlutaMAX containing 20% FBS (Gibco) and 100 IU per ml penicillin–streptomycin (P/S; Gibco). Solid tumor cell lines were cultured in RPMI-1640 with GlutaMAX containing 10% FBS and 100 IU per ml P/S. Breast epithelial cell lines were cultured using the mammary epithelial cell growth medium bullet kit (Lonza). Human B-ALL (Supplementary Table 2) and MCL (Supplementary Table 3) PDXs were cultured in MEMα (Life Technologies) with GlutaMAX containing 20% FBS and 100 IU per ml P/S. CLL samples (Supplementary Table 4) were cultured in RPMI-1640 with GlutaMAX containing 20% FBS, 100 IU per ml P/S, 50 ng ml^−1^ interleukin 2 (IL-2; Peprotech) and 1 μg ml^−1^ CpG. All cells were cultured at 37 °C in a humidified incubator with 5% CO_2_; they were authenticated and tested negative for *Mycoplasma* (MycoAlert PLUS, Lonza).

### Animal experiments

All experiments were approved by the regional council in Freiburg or Yale University and carried out in accordance with the German Animal Welfare Act (TVA: 35-9185.81/G-15/157) and Institutional Animal Care and Use Committee (protocol no. 20345). Mice were maintained in a pathogen-free environment at 18–23 °C with 40–60% humidity and a 14-h light and 10-h dark cycle. *Ctnnb1*^ex3fl^ (referred to as the β-catenin^(S33;S45)+/fl^)^2^ strain was provided by M. Taketo. *Apc*^fl/+^ mice^54^ were provided by Z. Qian. *Vav*^Cre^ *Csnk1a* ^fl/+^ mice^53^ were provided by B. Ebert and crossed with B6 mice to generate *Csnk1a1*^fl/+^ mice. To activate or delete β-catenin in B cell precursors, *Mb1*^Cre^ mice were crossed with β-catenin^(S33-S45)+/fl^ and β-catenin^fl/fl^ (ref. ^11^) mice, respectively. For dual-reporter mice, eGFP–Myc and mTq–β-catenin mice were crossed. All other mice were purchased from Jackson Laboratory. Genotyping of the mice was performed using the primers described in Supplementary Table 5. *Csnk1a1* mice were genotyped by Transnetyx. Both female and male mice were used and control and experimental groups were age and sex matched.

For transplantation experiments, 8–12-week-old sublethally irradiated NSG mice (200 cGy) were used. For limiting dilution of β-catenin^(S33;S45)+/fl^ cells, pre-B cells were transduced with MSCV-BCR-ABL1-IRES-LUC and Il-7 was removed to transform the cells. Cells were subsequently transduced with tamoxifen-inducible Cre (Cre-ER^T2^) and GFP or GFP (ER^T2^). GFP^+^ cells were sorted by fluorescence-activated cell sorting (FACS) and treated with 4-hydroxy-tamoxifen (4-OHT; 1 μM) for 2 days; 120,000, 6,000 or 300 cells were injected through the tail vein.

For GSK3β inhibitor treatment, cells were labeled with luciferase and selected with 25 μg ml^−1^ blasticidin; 1 million cells were injected. Then, 3 days after transplantation, LY2090314 or vehicle control (dissolved in 5% DMSO, 45% PEG300 and PBS) was administered intraperitoneally at a dose of 12.5 mg kg^−1^ twice per day for 4 days per week (every 12 h). Mice that displayed the signs of leukemia such as hunched back, weight loss and inability to move were killed. To measure leukemia burden, 2.5 mg of D-luciferin (Promega) was injected intraperitoneally and mice were anesthetized with 5% isoflurane for 15 min. Luminescent signal was measured by Lago X (Accela) and mice continued receiving 2% isoflurane through a nose cone during imaging. For histology analysis, tissues were fixed in Safe Fix II (Fischer Scientific) and embedded in paraffin. Before fixing, the mouse intestine was flushed with PBS to remove feces. Paraffin blocks were sectioned at 4 μm and stained with hematoxylin and eosin by the Yale Pathology Tissue Services. Images were acquired using Cytation5 (Biotek).

### Murine pre-B and B-ALL cells

Bone marrow cells were obtained by flushing the femur and tibia with PBS and filtering through a 70-μm mesh. Cells from spleen and lymph nodes were extracted using a 40-μm strainer. Erythrocytes were lysed for bone marrow and spleen cells using red blood cell lysis buffer (Biolegend). Bone marrow cells were cultured in IMDM (Gibco) with GlutaMAX containing 20% FBS, 50 μmol ml^−1^ 2-mercaptoethanol, 100 IU per ml P/S and 10 ng ml^−1^ IL-7 (Peprotech) to generate pre-B cell cultures. For the *BCR*–*ABL1*-driven leukemia model, pre-B cells were transduced by *BCR*–*ABL1* and IL-7 was removed to enrich transformed cells. For the *NRAS*^G12D^ model, pre-B cells were transduced by *NRAS*^G12D^ and cultured in the same conditions as pre-B cells.

### Retroviral and lentiviral transduction

Lenti-X HEK293T cells were transfected with Lipofectamine 2000 (Invitrogen) according to the manufacturer’s instructions and cultured in high-glucose DMEM (Gibco) with GlutaMAX containing 10% FBS, 100 IU per ml P/S (Gibco), 1 mmol l^−1^ sodium pyruvate (Gibco) and 0.1 mmol l^−1^ nonessential amino acids (Gibco). The next day, cells were treated with 10 mmol l^−1^ Sodium butyrate (Sigma-Aldrich) for 6–8 h and the medium was replaced. Then, 24 h later, supernatants were collected and filtered through a 0.45-μm filter. For all transductions, non-tissue-culture plates were coated with Retronectin (50 μg ml^−1^; Takara). For retroviral transductions, viral supernatants were loaded onto plates by centrifugation at 2,000*g* for 90 min at 32 °C and 2–3 million cells were centrifuged at 600*g* for 30 min. For lentiviral transduction, 2–4 million cells per well were centrifuged at 600*g* for 30 min in the presence of lentiviral supernatant and the medium was changed 16 h later.

### Fluorescent protein stability assay for β-catenin

Cells were transduced with vectors expressing β-catenin fused with GFP tag and mScarlet or EV with GFP and mScarlet expression. FACS analysis was performed for detecting frequencies of cells expressing WT β-catenin–GFP fusion within mScarlet^+^ cells. GFP to mScarlet signal was confirmed to be 1:1 in cells expressing EV.

### Reprogramming of B-ALL into myeloid lineage

B-ALL cells were transduced with Tet3G-neomycin vector (Takara) and subjected to neomycin (1 mg ml^−1^) selection and subsequently transduced with Tre-3G-driven C/EBPα or EV (puromycin resistance). Reprograming was induced by doxycycline (1 μg ml^−1^) treatment and myeloid conversion was studied by FACS by analyzing CD19 and CD11b expression.

### TOP-Flash (7TFC) assay

B-ALL cells were transduced with 7TFC lentiviral vector (Addgene, 24307) and mCherry^+^ cells were sorted by FACSAria III. A total of 100,000 cells were seeded in a 96-well plate in 80% of Wnt3A-conditioned medium from the L-Wnt3A cell line (American Type Culture Collection, CRL-2647) and 20% MEMα containing 20% fetal calf serum. Luciferase activity was measured by adding 100 µl of ONE-Glo buffer from Promega (E6120) using a GloMax plate reader (Promega).

### Cell viability assay

In total, 10,000–40,000 cells were seeded per well in a 96-well plate (Eppendorf) in 80 μl of medium. Inhibitors were added at the indicated concentrations to achieve a total volume of 100 μl. After 3 days, Cell Titer Glo 2.0 assays (Promega) were performed according to the manufacturer’s instructions. Relative viabilities were calculated by measuring the luminescence value and normalizing to the values of untreated cells.

### Western blotting

Cells were washed twice with ice-cold PBS and lysed in CelLytic buffer (Sigma-Aldrich) supplemented with 1% protease inhibitor (Roche Diagnostics) and 1% phosphatase inhibitor (EMD Millipore) and 1 mM PMSF (Cell Signaling Technologies) on ice for 15 min. Then, 10–20 µg of cell lysates were separated on precast gels (Bio-Rad) and transferred to nitrocellulose membranes (Bio-Rad). Membranes were blocked with 2% BSA in Tris-buffered saline with Tween-20 for 1 h at room temperature and then probed with appropriate primary antibodies at 4 °C overnight. Membranes were incubated with AP-conjugated secondary antibodies (Invitrogen) for 1 h at room temperature and analyzed with Chemi Doc MP imaging system (Bio-Rad). Fractionation experiments were performed using nuclear and cytoplasmic extraction reagent (Thermo Scientific) according to the manufacturer’s instructions.

### Flow cytometry

Cells were washed twice with PBS containing 2% FBS and blocked with Fc blocker (BD Biosciences) for 20 min followed by antibody or isotype control staining for 30 min at 4 °C. Cells were then washed and resuspended with DAPI (0.75 μg ml^−1^) to exclude dead cells and analyzed on LSRFortessa X-20 or FACSSympony A3 (BD Biosciences). Cells were sorted using FACSAria III or FACSAria Fusion (BD Biosciences). FACS data were analyzed using FlowJo software (FlowJo).

### Live-cell time-lapse imaging

For live-cell imaging, cells were cultured in phenol-red-free MEMα (Gibco), 1% P/S and 10% FBS at 37 °C with 5% CO_2_. Imaging plates (µ-Slide eight-well high-glass-bottom plates) were coated with poly(L-lysine) for 2 h before loading cells to minimize cell movement. Images were acquired with an Andor Dragonfly spinning disk confocal microscope using a ×40 oil objective. Each individual frame was performed every 20 min over the course of a 24-h time course. Images were processed using ImageJ.

### CRISPR-mediated gene deletion

CRISPR RNAs (crRNAs; Supplementary Table 5; 100 µmol l^−1^) and *trans*-activating crRNAs (tracrRNAs; 100 µmol l^−1^) were annealed by incubation at 95 °C for 5 min. Recombinant Cas9 (40 µmol l^−1^) was incubated with gRNA mixture for 10–20 min at room temperature and Cas9–gRNA complexes were electroporated using the Neon transfection system (Invitrogen). For *Ikzf1* and/or *Ikzf3* deletion in mouse B-ALL cells and *CTNNB1* deletion in BV173 cells, single-cell-derived knockout cell lines were generated.

### HDR-mediated gene editing

For editing the BENC region, gRNA complex was formed by annealing crRNAs (Supplementary Table 5) and tracrRNAs at 95 °C for 5 min. Recombinant Cas9 (Integrated DNA Technologies (IDT); 125 pmol) was incubated with gRNA (150 pmol) to form RNP complexes. Mouse B-ALL cells were resuspended in Lonza P4 buffer and electroporated with the RNP complex, single-stranded DNA HDR donor oligos (Supplementary Table 5; IDT) and Alt-R Cas9 electroporation enhancer (IDT) using Nucleofector (Lonza, pulse code CV104). Following electroporation, cells were treated with Alt-R HDR enhancer V2 (1 µmol^−1^; IDT) for 24 h. To assess HDR editing, gDNA was extracted using Nucleospin tissue (Macherey-Nagel) following the manufacturer’s protocol and PCR amplification using Q5 high-fidelity master mix (New England Biolabs). PCR amplicons were digested with EcoRI-HF (New England Biolabs) in 1× CutSmart buffer (New England Biolabs) at 37 °C for 4 h and analyzed on 2% agarose gel. Single-cell-derived cell lines were generated and Sanger sequencing was performed.

For β-catenin deletion in PDX2 cells, double-stranded DNA HDR template was amplified using TrueTag knockout enrichment donor template (puromycin–RFP; Invitrogen) and primers listed in Supplementary Table 5. crRNAs and tracrRNAs were annealed at 95 °C for 5 min, followed by incubating with Cas9 (125 pmol) for 15 min at room temperature. PDX2 cells were resuspended in Lonza SF buffer and electroporated with the RNP complex, double-stranded HDR template and Alt-R Cas9 electroporation enhancer (IDT) using Nucleofector (Lonza, pulse code ED-120). β-catenin knockout cells were enriched by sorting for RFP^+^ cells and selecting for puromycin resistance (0.6 μg ml^−1^).

### Colony formation assay

A total of 10,000 B-ALL cells were plated on MethoCult medium (Stem Cell Technologies) in 3-cm-diameter dishes and colonies were counted after 10–14 days. For *BCR*–*ABL1* transformed cells, medium without cytokines (M3231) was used; for *NRAS*^G12D^ transformed cells, medium with IL-7 (M3630) was used.

### mRNA-seq and analysis

RNA was isolated using Macherey-Nagel RNA extraction kit according to the manufacturer’s instructions. Sequencing was performed on an Illumina Hiseq 2500 instrument using the TruSeq paired-end cluster kit V4-cBot-HS (Illumina) to generate 101 bp paired-end reads for sequencing with v4 chemistry. Quality control of RNA-seq reads was performed using FastQC (version 0.11.9), SAMtools (version 1.7) and Picard (version 2.23.8). Transcripts were quantified with Salmon (version 1.4.0)^59^ and reads were aligned using STAR (version 2.7.6)^60^ to the mouse genome (mm10/GRCm38, gencode vM24). Downstream analysis was performed in R^61^; differential expression was analyzed with DESeq2 (version 1.30.1)^62^ with standard models and normal shrinkage estimators. Gene set enrichment analyses (GSEAs) were performed with fgsea (version 1.16.0) using log_2_ fold changes from DESeq2 and gene sets from MSigDB or internal data as indicated.

### Co-IP and proteomic analysis

Co-IP experiments were performed using the Pierce crosslink magnetic IP/co-IP kit according to the manufacturer’s instructions (Thermo Scientific). Anti-β-catenin (14; BD Biosciences) or isotype (107.3; BD Biosciences) antibody were coupled to protein A/G beads and covalently crosslinked with 20 µmol l^−1^ DSS; beads were incubated with cell lysates at 4 °C overnight. Beads were washed twice, eluted in a low-pH elution buffer and subjected to mass spectrometry (Northwestern Proteomics Core Facility). Peptides were analyzed by liquid chromatography–tandem mass spectrometry using a Dionex UltiMate 3000 rapid separation liquid chromatography system and an Orbitrap mass spectrometer (Thermo Fisher Scientific).

Downstream analysis of proteomic datasets was performed in R^61^; protein values were quantile-normalized and mixed imputations were used to estimate missing values. Missing values were classified as missing not at random (MNAR) if proteins were detected in fewer than two replicates from a condition and missing at random (MAR) otherwise. MNAR values were imputed by minimum probability, whereas MAR values were estimated by maximum-likelihood imputation using the MSnbase and DEP packages^63^. Normalized, imputed values were used in linear modeling and empirical Bayes testing for differentially enriched proteins between conditions using limma and DEP packages^63,64^; differential enrichment results are given in pulldown experiments. For visualization, fold change over Ig background binding was further divided by average background binding detected in CRAPome to downweight common contaminants^65^.

### ChIP experiments

The ChIP-Rx method was applied to mapping histone marks among various cell conditions^66^. In each experiment, B-ALL cells were fixed with 1% formaldehyde for 10 min at room temperature and quenched by 125 mmol l^−1^ glycine. For ChIP-seq of IKZF1 and IKZF3, B-ALL cells were crosslinked by 2 mmol l^−1^ disuccinimidyl glutarate for 45 min and 1% formaldehyde for 10 min at room temperature before chromatin enrichment and library construction. Nuclei were lysed with SDS lysis buffer and sonicated using Bioruptor (Diagenode). After sonication, cell debris was removed by centrifugation (13,000*g*, 10 min) and supernatants were diluted and precleared by incubation with protein A/G Dynabeads (Invitrogen) conjugated to an isotype control. ChIP reactions were performed using antibodies specifically recognizing H3K27ac (39133, Active Motif), H3K4me3 (MC315, Millipore), IKZF1 (GTX129438, GeneTex) and IKZF3 (D1C1E, CST). ChIPed DNA was reverse-crosslinked overnight at 65 °C and purified by a QIAquick PCR purification kit (Qiagen). ChIP-seq libraries were constructed using a SMARTer ThruPLEX DNA-seq kit (Takara) and subjected to Illumina deep sequencing. β-catenin ChIP was performed by Active Motif using the clone CAT-15 (Thermo). Quality control was performed using FastQC (version 0.11.9) and ChIPQC. Reads were aligned with BWA (version 0.7.17)^67^ against the mouse genome (mm10/GRCm38, gencode vM24). Peak calling was performed with MACS2 (version 2.2.7.1). Downstream analysis was performed in R^61^; differential binding was analyzed with DiffBind (version 3.0.15)^68^. Peaks with −log_10_ *q* value > 10 in two or more conditions were retained after blacklisting and graylisting. For transcription factors (IKZF1, IKZF3 and CTNNB1), within-peak normalization was applied, whereas whole-genome normalization was applied to histone modification data. Annotation was performed with ChIPpeakAnno^69^ to the closest transcription start site, except for the BENC enhancer region, which was manually annotated as described previously^46^.

For ChIP–qPCR, protein A/G beads were incubated with antibodies recognizing MTA2 (8106, Abcam), CHD4 (72418, Abcam) or isotype control (910801, Biolegend) for 4 h at 4 °C. Precleared DNA fragments were incubated with the antibody-conjugated magnetic beads for 16 h at 4 °C. Crosslinking was reversed for 6 h at 65 °C and DNA was purified with the NucleoSpin gel and PCR cleanup kit (Macherey-Nagel). qPCR was performed with PowerUp SYBR green master mix (Applied Biosystems) using a ViiA7 real-time PCR system (Applied Biosystems). Data were analyzed using QuantStudio real-time PCR software (Thermo Fisher Scientific) and amplification values from ChIPed DNA were normalized to input DNA.

### Tissue microarray (TMA) analysis

Participant biopsies were obtained in compliance with the internal review board of the Beckman Research Institute and TMAs were constructed. Formalin-fixed paraffin-embedded TMAs were cut at 4 μm. TMAs were processed on a Ventana Discovery Ultra IHC automated stainer (Ventana Medical Systems). This included deparaffinization, rehydration, endogenous peroxidase activity inhibition and antigen retrieval. The TMAs were stained with anti-human β-catenin antibody (clone 14, Roche) or β-catenin pS37 antibody (ab47335, Abcam), followed by anti-mouse HQ secondary antibody (Discovery) and an anti-HQ horseradish peroxidase detection system (Discovery). The stains were visualized with a ChromoMap DAB kit (DISCOVERY), counterstained with hematoxylin (Ventana).

### Compound screen sensitivity analysis

Data from GDSC1, GDSC2 and CTD2 screens were downloaded from DepMap (https://depmap.org/)^35^ and analyzed in R using the gdscic50 package for normalization^70^. Four-parameter log-logistic dose–response curves were fit for all datasets using the drc package^71^. The ΔAAC and statistics were calculated between B cell and solid tumor cell lines for each drug screen separately and combined into a single differential score by weighted meta-analysis using the fraction of response curves within an EC window (defined as minima and maxima fitting of *P* < 0.05 and response > 50%) as a weighting parameter to downweight screens using ineffective concentration ranges. Final combined effect scores were used for volcano plots and as input to feature set enrichment analysis; feature sets used were any compound groups with common targets and *n* > 5 compounds.

### Chemogenomic CRISPR–Cas9 knockout screen

CRISPR screen was performed by ChemoGenix (Institute for Research in Immunology and Cancer, Université de Montréal) as previously described^72^. Briefly, the NALM6 clone with inducible Cas9 expression (Addgene, 50661) was transduced with the genome-wide knockout EKO sgRNA library (278,754 different sgRNAs)^50^ and knockouts were induced by doxycycline (2 μg ml^−1^) for 7 days. Cells were treated with LY2090314 (3.5 nM) or DMSO for 8 days and gDNA was extracted using the Gentra Puregene kit (Qiagen) according to the manufacturer’s instructions. sgRNA sequences were PCR-amplified as described previously^50^ and analyzed by next-generation sequencing (Illumina NextSeq 500). Reads were aligned using Bowtie 2.2.5 in the forward direction only (‘--norc’ option) and total read counts per sgRNA were tabulated. Context-dependent chemogenomic interaction scores were calculated using a modified version of the RANKS algorithm^50^ to distinguish chemogenomic interactions from essentiality phenotypes. Raw read counts are available upon request from ChemoGenix.

### Statistics and reproducibility

Data are shown as the mean ± s.d. unless stated otherwise. Data distribution was assumed to be normal but this was not formally tested. Statistical analysis was performed by Prism 9 (GraphPad) using an unpaired two-tailed *t*-test or log-rank test as indicated in the figure legends. *P* < 0.05 was considered significant. Kaplan–Meier survival analysis was used to estimate overall survival. No statistical methods were used to predetermine sample sizes but our sample sizes are comparable to those reported in previous publications. No data were excluded from the analysis. The experiments were not randomized. The investigators were not blinded to allocation during experiments and outcome assessment. Experiments were repeated to ensure reproducibility. Half-maximal inhibitory concentration values were estimated using default dose–response models in drc (version 3.0.1)^73^ with an upper limit of 1.

### Reporting summary

Further information on research design is available in the Nature Portfolio Reporting Summary linked to this article.

## Supplementary information

Supplementary Information Gating strategy for FACS analysis.

Reporting Summary

Supplementary Video 1 Time-lapse imaging of β-catenin Myc dual reporter B-ALL cells following LY2090314 treatment

Supplementary Table 1–5 Supplementary Tables 1–5.

## Source data

Source Data Fig. 1-6 and Source Data Extended Data Fig. 2, 4, 5, 6, 8 Unprocessed western blots and/or gels.

Source Data Fig. 1 Statistical source data.

Source Data Fig. 2 Statistical source data.

Source Data Fig. 3 Statistical source data.

Source Data Fig. 4 Statistical source data.

Source Data Fig. 5 Statistical source data.

Source Data Fig. 6 Statistical source data.

Source Data Fig. 7 Statistical source data.

Source Data Extended Data Fig. 4 Statistical source data.

Source Data Extended Data Fig. 5 Statistical source data.

Source Data Extended Data Fig. 6 Statistical source data.

Source Data Extended Data Fig. 7 Statistical source data.

Source Data Extended Data Fig. 9 Statistical source data.

## Data Availability

The mutation data for *CTNNB1*, *APC*, *AXIN1*, *AXIN2*, *CSNK1a* and *GSK3B* genes (Fig. 2a) were acquired from COSMIC^36^. The data for IHC staining of β-catenin in normal tissues (Extended Data Fig. 2a,b) were obtained from the Human Protein Atlas^34^ (https://www.proteinatlas.org/). RNA-seq and RPPA data (Figs. 1c and 6c) and compound screen data (Fig. 6a–c,e and Extended Data Fig. 9a) were obtained from DepMap (https://depmap.org/). Hallmark Myc-Targets V1 and Hallmark Wnt–β-catenin signaling gene sets were acquired from MSigDB (https://www.gsea-msigdb.org/gsea/msigdb). ChIP-seq and RNA-seq data that support the findings of this study were deposited to the Gene Expression Omnibus under accession codes GSE305472, GSE196767, GSE245287 and GSE196745. Mass spectrometry data were deposited to the PRIDE repository under accession codes PXD067271, PXD067314, PXD067306 and PXD067318. All other data are available from the corresponding author upon reasonable request. Source data are provided with this paper.
