# Supplementary material for: Targeting β-catenin degradation with GSK3β inhibitors induces cell death in acute lymphoblastic leukemia
Source: Nat Cancer. 2026 Jan 8;7(1):150–68. doi: 10.1038/s43018-025-01093-z (PMC12858398; doi:10.1038/s43018-025-01093-z)
Supplement: Supplementary file 1 — Gating strategy for FACS analysis. [file 43018_2025_1093_MOESM1_ESM.pdf]

nature cancer

Supplementary information https://doi.org/10.1038/s43018-025-01093-z

Targeting β-catenin degradation with GSK3β inhibitors induces cell death in acute lymphoblastic leukemia

In the format provided by the authors and unedited

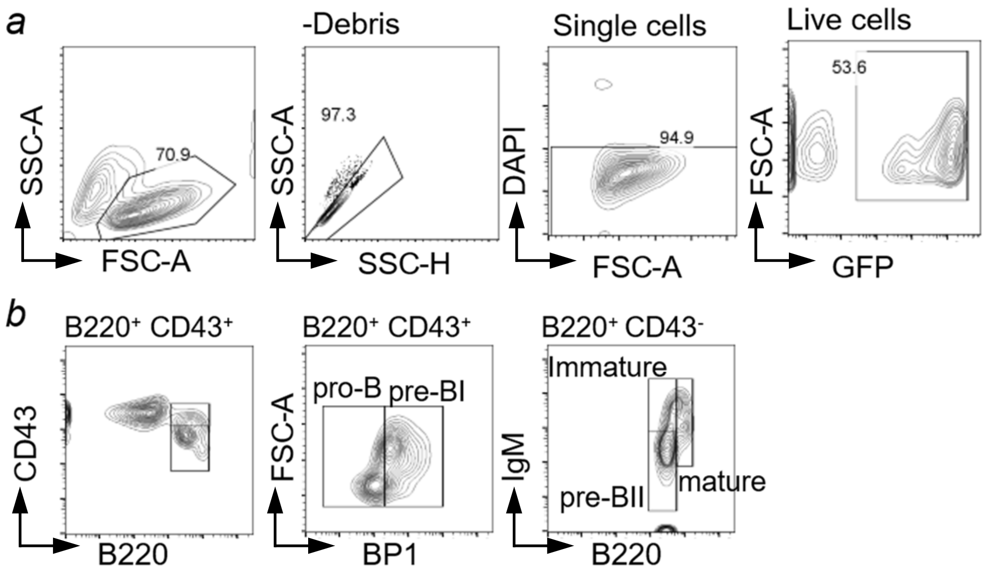

Supplementary Figure 1. a) Representative gating strategy for growth competition assays presented in Figures 2b,d,f,l, 4i, 5h, Extended Data Fig. 5a-d. **b**) Representative gating strategy for analysis of bone marrow progenitors (Extended Data Fig. 5e-f).
