# Supplementary material for: Targeting β-catenin degradation with GSK3β inhibitors induces cell death in acute lymphoblastic leukemia
Source: Nat Cancer. 2026 Jan 8;7(1):150–68. doi: 10.1038/s43018-025-01093-z (PMC12858398; doi:10.1038/s43018-025-01093-z)
Supplement: Supplementary file 2 — Reporting Summary [file 43018_2025_1093_MOESM2_ESM.pdf]

nature portfolio

Corresponding author(s): Markus Müschen

Last updated by author(s): 2025/10/03

Reporting Summary

Nature Portfolio wishes to improve the reproducibility of the work that we publish. This form provides structure for consistency and transparency in reporting. For further information on Nature Portfolio policies, see our Editorial Policies and the Editorial Policy Checklist.

Statistics

For all statistical analyses, confirm that the following items are present in the figure legend, table legend, main text, or Methods section.

n/a	Confirmed	
☐	☒	The exact sample size (*n*) for each experimental group/condition, given as a discrete number and unit of measurement
☐	☒	A statement on whether measurements were taken from distinct samples or whether the same sample was measured repeatedly
☐	☒	The statistical test(s) used AND whether they are one- or two-sided<br>*Only common tests should be described solely by name; describe more complex techniques in the Methods section.*
☒	☐	A description of all covariates tested
☒	☐	A description of any assumptions or corrections, such as tests of normality and adjustment for multiple comparisons
☐	☒	A full description of the statistical parameters including central tendency (e.g. means) or other basic estimates (e.g. regression coefficient) AND variation (e.g. standard deviation) or associated estimates of uncertainty (e.g. confidence intervals)
☐	☒	For null hypothesis testing, the test statistic (e.g. *F*, *t*, *r*) with confidence intervals, effect sizes, degrees of freedom and *P* value noted<br>*Give P values as exact values whenever suitable.*
☒	☐	For Bayesian analysis, information on the choice of priors and Markov chain Monte Carlo settings
☒	☐	For hierarchical and complex designs, identification of the appropriate level for tests and full reporting of outcomes
☐	☒	Estimates of effect sizes (e.g. Cohen's *d*, Pearson's *r*), indicating how they were calculated

Our web collection on statistics for biologists contains articles on many of the points above.

Software and code

Policy information about availability of computer code

Data collection

FACS data was acquired using FACS Diva software.
For protein estimations and Cell Titer Glo assaysSoftmax pro7 (Molecular devices) software was used.
Western blot images were collected by Biorad Image Lab Touch Software.
For colony forming assays, images were acquired GelCount TM 1.2.1.0 (Oxford Optronix).
To determine the number of viable cells, the trypan blue exclusion method was applied, using the Countess II FL Automated Cell Counter.
The luminescence signal for in vivo expansion leukemic cells were acquired by Amiview (Alternative Micrographics).
Microscopy images were taken by Gen5.3.11 (Agilent).
qPCR data was acquired by Quantstudio (Thermo Fischer).
Live cell imaging with b-catenin reporter cells was performed using Fusion.

Data analysis

FACS data were analyzed using FlowJo 10.10.0 software (FlowJo, LLC) and GraphPad Prism 9.
For colony forming assay, number of colonies were counted by GelCount TM 1.2.1.0 (Oxford Optronix) and analyzed by GraphPad Prism 9 and Excel.
For in vivo leukemia burden analysis, Amiview (Alternative Micrographics) was used.
For comparing the survival of mice, Kaplan-meier analysis and log-rank test were performed using GraphPad Prism 9.
Images from live cell imaging was processed by ImageJ.
For proteomics analysis MS/MS spectra were searched using Thermo Proteome Discoverer 2.3/Byonic 2.15 - Protein Metrics.
For Co-IP experiment downstream analysis of proteomic data sets was performed in R using MSnbase v2.10.1, limma v3.40.6 and DEP v1.6.1 packages.
CRAPome database was used to to down-weight common contaminants of mass-spec data.
TMAs were analyzed using Ventana Image Viewer and QuPath 0.3.2.

For analysis of RNA seq data, and reads aligned using STAR v2.7.62.
Differential expression was analyzed with DESeq2 v1.30.14 with standard models and normal shrinkage estimators.
Gene set enrichment analyses were performed with fgsea v1.16.0 using log2 fold change estimates from DESeq2.qPCR.

For manuscripts utilizing custom algorithms or software that are central to the research but not yet described in published literature, software must be made available to editors and reviewers. We strongly encourage code deposition in a community repository (e.g. GitHub). See the Nature Portfolio guidelines for submitting code & software for further information.

Data

Policy information about availability of data

All manuscripts must include a data availability statement. This statement should provide the following information, where applicable:

- Accession codes, unique identifiers, or web links for publicly available datasets
- A description of any restrictions on data availability
- For clinical datasets or third party data, please ensure that the statement adheres to our policy

The mutation data for CTNNB1, APC, AXIN1, AXIN2, CSNK1a and GSK3β genes (Figure 2a) was acquired from https://cancer.sanger.ac.uk/cosmic.
The data for IHC staining of β-catenin in normal tissues (Extended Data Figure 2a, b) was obtained from https://www.proteinatlas.org/.
RNA-seq and RPPA data for quantifying expression levels of β-catenin in human cancer cell lines (Figure 1c, 2e, 6c) were obtained from https://depmap.org/portal/download.
Compound screen data used to compare drug responses in solid tumor and B-cell leukemia/lymphoma cell lines to was acquired from https://depmap.org/portal/download (Figure 6a-b, c , Extended Data Figure 9a).
Hallmark Myc-Targets V1 and Hallmark Wnt/β-catenin signaling gene sets were acquired from MSigDB.
RNA-seq data for β-catenin activation in Ikzf1/3 knock-out or wild-type cells have been deposited to GEO (GSE305472, GSE196767).
RNA-seq data for LY2090314 mediated GSK3B inhibition in β-catenin wild type or knock-out cells has been deposited to GEO (GSE245287).
CHIP-seq data for Ikzf1, Ikzf3, Ctnnb1, H3K27Ac and H3K4Me3 have been deposited to GEO (GSE196745).
Proteomic data has been uploaded to Pride database (PXD067271, PXD067314, PXD067306, PXD067318).
For chemogenomics data, raw read counts are available upon request from ChemoGenix.
All other data are available from the corresponding author upon reasonable request.

Research involving human participants, their data, or biological material

Policy information about studies with human participants or human data. See also policy information about sex, gender (identity/presentation), and sexual orientation and race, ethnicity and racism.

Reporting on sex and gender	Both male and female patient samples were included in the study and this information was provided in Supplementary tables 2-4.
Reporting on race, ethnicity, or other socially relevant groupings	Patient samples were not selected based on race, ethnicity or other socially relevant groupings.
Population characteristics	Age, genotype information on the patients samples was provided in Supplementary tables 2-4.
Recruitment	N/A
Ethics oversight	Patient samples were obtained in compliance with the Institutional Review Boards of University of California San Francisco, City of Hope, Yale University and Dana-Farber Cancer Institute.Written informed consent was obtained from all participants.

Note that full information on the approval of the study protocol must also be provided in the manuscript.

Field-specific reporting

Please select the one below that is the best fit for your research. If you are not sure, read the appropriate sections before making your selection.

☒ Life sciences ☐ Behavioural & social sciences ☐ Ecological, evolutionary & environmental sciences

For a reference copy of the document with all sections, see nature.com/documents/nr-reporting-summary-flat.pdf

Life sciences study design

All studies must disclose on these points even when the disclosure is negative.

Sample size	For mouse experiments at least three mice per genotype were used for each analysis to determine a biological meaningful difference between compared genotypes. The precise number of sample used in indicated in the manuscript. For experiments involving human cell lines, experiments were repeated in different cell lines when possible. No statistical methods were used to pre-determine sample sizes but our sample sizes are like those reported in previous publications (Chan et. al., Nature, 2020).
Data exclusions	For all FACS analysis, only live single cells were included in further analysis. Duplets determined by SSC-H/SSC-A and dead cells defined by DAPI positivity were excluded. Debris were excluded by SSC-A/FSC-A gate.

Replication	The results were reliably reproduced using biological replicates and different cell lines.
Randomization	Randomization was not used.
Blinding	Blinding was not used as our study does not involve subjective assessments or interventions.

Reporting for specific materials, systems and methods

We require information from authors about some types of materials, experimental systems and methods used in many studies. Here, indicate whether each material, system or method listed is relevant to your study. If you are not sure if a list item applies to your research, read the appropriate section before selecting a response.

Materials & experimental systems

n/a	Involved in the study
☐	☒ Antibodies
☐	☒ Eukaryotic cell lines
☐	☐ Palaeontology and archaeology
☐	☒ Animals and other organisms
☐	☐ Clinical data
☐	☐ Dual use research of concern
☐	☐ Plants

Methods

n/a	Involved in the study
☐	☒ ChIP-seq
☐	☒ Flow cytometry
☐	☐ MRI-based neuroimaging

Antibodies

Antibodies used

Apc KT-45 Invitrogen 1:1000 WB
Axin1 C76H11 Cell Signaling Technology 1:1000 WB
β-actin SC-47778 Santa Cruz Biotech 1:2000 WB
β-catenin 14/Beta-Catenin BD 1:1000 WB, Co-IP 10ug/reaction
Non-phospho β-Catenin D13A1 CST 1:1000 WB
β-tubulin D2N5G Cell Signaling Technology 1:1000 WB
phospho S33/S37/T41 β-Catenin Cell Signaling Technology 1:1000 WB
Casein kinase Cell Signaling Technology 1:1000 WB
C/ebp alpha1 D56F10 Cell Signaling Technology 1:1000 WB
Chd (Mi-2β) 72418 Abcam 1:1000 WB, CHIP-qPCR 5ug/reaction
Gatad2a ab87663 Abcam 1:1000 WB
Gsk3b D5C5Z Cell Signaling Technology 1:1000 WB
Ikzf1 D6N9Y Cell Signaling Technology 1:1000 WB
Ikzf2 D8W4X Cell Signaling Technology 1:1000 WB
Ikzf3 D1C1E Cell Signaling Technology 1:1000 WB, CHIP 5ug/per reaction
Mta2 8106 Abcam 1:1000 WB, CHIP-qPCR 5ug/reaction
Myc D84C12 Cell Signaling Technology 1:1000 WB
Prdm1 C14A4 Cell Signaling Technology 1:1000 WB
Tbp D5C9H Cell Signaling Technology 1:1000 WB
Tcf1/Tcf7 C63D9 Cell Signaling Technology 1:1000 WB
Tcf4/Tcf7l2 C48H11 Cell Signaling Technology 1:1000 WB
Stat5 D2O6Y Cell Signaling Technology 1:1000 WB
Rabbit Polyclonal (Poly29108) IgG Biolegend CHIP-qPCR control Ig 5ug/reaction
Polyclonal anti-H3K27ac Active Motif CHIP 5ug/per reaction
anti-H3K4me3 MC315 Millipore CHIP 5ug/per reaction
Polyclonal anti-beta catenin (CAT-15) Thermo CHIP
Polyclonal anti-Ikaros Genetex CHIP CHIP 5ug/per reaction
Polyclonal anti-phospho S37 β-Catenin Abcam ab47335 TMA analysis
Mouse IgG1 κ Isotype Control 107.3 BD Co-IP 10ug/reaction
B220 (CD45R) PE RA3-6B2 Biolegend FACS 1:100
BP-1 (Ly-51) 6C3 Invitrogen FACS 1:50
CD11b PE M1/70 Biolegend FACS 1:100
Cd19 APC 6D5 Biolegend FACS 1:100
IgM PerCy5.5 R6-60.2 BD FACS 1:100
CD10 PE CF594 HI10a BD Biosciences 1:50
CD117 PE YB5.B8 BD Biosciences FACS 1:50
CD127 PE-Cy7 A019D5 Biolegend FACS 1:50
CD179a APC HSL96 Miltenyi FACS 1:50
CD19 APC Cy7 HIB19 Biolegend FACS 1:50
CD20 BB515 2H7 BD Biosciences FACS 1:50
CD34 APC 581 BD Biosciences FACS 1:50
CD45 BV711 HI30 BD Biosciences FACS 1:50
IgD BB515 IA6-2 BD Biosciences FACS 1:50
IgM PerCy5.5 G20-127 BD Biosciences FACS 1:50

TSLPR PE 1F11/TSLPR BD Biosciences FACS 1:50

Validation

All antibodies used have been tested and validated by the supplier and also by other researchers in the field.
Apc KT-45 https://www.thermofisher.com/antibody/product/Protein-APC-Antibody-clone-KT45-Monoclonal/MA1-72530
Axin1 C76H11 https://www.cellsignal.com/products/primary-antibodies/axin1-c76h11-rabbit-mab/2087?srsltid=AfmBOops3Xibbu1wPuie7iM5GYdOvusliA_aEbSscg50UFSzIkhNRx9K
β-actin https://datasheets.scbt.com/sc-47778.pdf
β-catenin 14/Beta-Catenin https://www.bdbiosciences.com/en-us/products/reagents/microscopy-imaging-reagents/immunofluorescence-reagents/purified-mouse-anti-catenin.610153
Non-phospho β-Catenin D13A1 https://www.cellsignal.com/products/8814/datasheet?images=1&protocol=0
β-tubulin D2N5G https://www.cellsignal.com/products/15115/datasheet?images=1&protocol=0
phospho S33/S37/T41 β-Catenin https://www.cellsignal.com/products/9561/datasheet?images=1&protocol=0
Casein kinase https://www.cellsignal.com/products/2655/datasheet?images=1&protocol=0
C/ebp alpha1 D56F10 1:1000 https://www.cellsignal.com/products/8178/datasheet?images=1&protocol=0
Chd (Mi-2β) 72418 https://doc.abcam.com/datasheets/active/ab72418/en-us/chd4-antibody-ab72418.pdf
Gatad2a ab87663 https://doc.abcam.com/datasheets/active/ab87663/en-us/gatad2a-antibody-ab87663.pdf
Gsk3b D5C5Z https://www.cellsignal.com/products/12456/datasheet?images=1&protocol=0
Ikzf1 D6N9Y https://www.cellsignal.com/products/14859/datasheet?images=1&protocol=0
Ikzf2 D8W4X https://www.cellsignal.com/products/42427/datasheet?images=1&protocol=0
Ikzf3 D1C1E https://www.cellsignal.com/products/15103/datasheet?images=0&protocol=0
Mta2 8106 https://www.abcam.com/en-us/products/primary-antibodies/mta2-pid-antibody-ab8106?srsltid=AfmBOoqVLLu_JdtUpVvs8ALKD7h1EC3s5on4Z0n-N1qwdfuwszBj3s9j
Myc D84C12 https://www.cellsignal.com/products/5605/datasheet?images=0&protocol=0
Prdm1 C14A4 https://www.cellsignal.com/products/9115/datasheet?images=0&protocol=0
Tbp D5C9H https://www.cellsignal.com/products/44059/datasheet?images=0&protocol=0
Tcf1/Tcf7 https://www.cellsignal.com/products/2203/datasheet?images=1&protocol=0
Tcf4/Tcf7l2 1:1000 https://www.cellsignal.com/products/2569/datasheet?images=1&protocol=0
Stat5 D2O6Y https://www.cellsignal.com/products/94205/datasheet?images=1&protocol=0
Rabbit Polyclonal (Poly29108) IgG https://www.biolegend.com/de-at/products/purified-rabbit-polyclonal-isotype-ctrl-antibody-11604
Polyclonal anti-H3K27ac https://www.activemotif.com/catalog/details/39133/histone-h3-acetyl-lys27-antibody-pab
anti-H3K4me3 MC315 https://www.sigmaaldrich.com/US/en/product/sigma/zrb04791
Polyclonal anti-beta catenin (CAT-15)https://www.thermofisher.com/antibody/product/beta-Catenin-Antibody-clone-CAT-15-Polyclonal/71-2700
Polyclonal anti-Ikaros Genetex https://www.genetex.com/Product/Detail/Ikaros-antibody/GTX129438?srsltid=AfmBOoqeZ0RUM0hixfnbWmxGKD8pvMDD8pEWtaD5rGzk2KqhNJ4Rvy-E
Polyclonal anti-phospho S37 β-Catenin https://www.abcam.com/en-us/products/primary-antibodies/beta-catenin-phospho-s37-antibody-ab47335
Mouse IgG1 κ Isotype Control 107.3 https://www.bdbiosciences.com/en-us/products/reagents/immunoassay-reagents/purified-na-le-mouse-igg1-isotype-control.553447?tab=product_details
B220 (CD45R) PE RA3-6B2 https://www.biolegend.com/nl-be/products/pe-anti-mouse-human-cd45r-b220-antibody-447
BP-1 (Ly-51) 6C3 https://www.thermofisher.com/antibody/product/CD249-BP-1-Antibody-clone-6C3-Monoclonal/14-5891-82
CD11b PE M1/70 https://www.biolegend.com/nl-be/products/pe-anti-mouse-human-cd11b-antibody-349?GroupID=BLG10552
Cd19 APC 6D5 https://www.biolegend.com/en-ie/products/apc-anti-mouse-cd19-antibody-1526
IgM R6-60.2 PerCy5.5 BD https://www.bdbiosciences.com/en-us/products/reagents/flow-cytometry-reagents/research-reagents/single-color-antibodies-ruo/percp-cy-5-5-rat-anti-mouse-igm.562034?tab=product_details
CD10 PE CF594 bdbiosciences.com/en-us/products/reagents/flow-cytometry-reagents/research-reagents/single-color-antibodies-ruo/pe-cf594-mouse-anti-human-cd10.562396
CD117 PE YB5.B8 https://www.bdbiosciences.com/en-us/products/reagents/flow-cytometry-reagents/research-reagents/single-color-antibodies-ruo/pe-mouse-anti-human-cd117.555714?tab=antibody_details
CD127 PE-Cy7 A019D5 https://www.biolegend.com/en-ie/products/pe-cyanine7-anti-human-cd127-22101
CD179a APC HSL96 https://www.miltenyibiotec.com/US-en/products/cd179a-vpreb-antibody-anti-human-hsl96.html
CD19 APC Cy7 HIB19 https://www.biolegend.com/en-ie/products/apc-cyanine7-anti-human-cd19-antibody-1910?GroupID=BLG10095
CD20 BB515 2H7 https://www.bdbiosciences.com/en-us/products/reagents/flow-cytometry-reagents/research-reagents/single-color-antibodies-ruo/bb515-mouse-anti-human-cd20.564568
CD34 APC 581 bdbiosciences.com/en-us/products/reagents/flow-cytometry-reagents/research-reagents/single-color-antibodies-ruo/apc-mouse-anti-human-cd34.555824
CD45 BV711 HI30 https://www.bdbiosciences.com/en-us/products/reagents/flow-cytometry-reagents/research-reagents/single-color-antibodies-ruo/bv711-mouse-anti-human-cd45.564358?tab=product_details
IgD BB515 IA6-2 https://www.bdbiosciences.com/en-at/products/reagents/flow-cytometry-reagents/research-reagents/single-color-antibodies-ruo/bb515-mouse-anti-human-igd.565243?tab=product_details
IgM PerCy5.5 G20-127 bdbiosciences.com/en-us/products/reagents/flow-cytometry-reagents/research-reagents/single-color-antibodies-ruo/percp-cy-5-5-mouse-anti-human-igm.561285
TSLPR PE 1F11/TSLPR https://www.bdbiosciences.com/en-us/products/reagents/flow-cytometry-reagents/research-reagents/single-color-antibodies-ruo/pe-mouse-anti-human-tslp-receptor.563149?tab=product_details

Eukaryotic cell lines

Policy information about cell lines and Sex and Gender in Research

Cell line source(s)	Detailed information of cell lines that are listed in Supplementary Table 1.<br>BV-173 DMSZ ACC 20<br>DAUDI DSMZ ACC-78<br>DLD1 ATCC CCL-221<br>DMS273 Sigma 95062830<br>GUMBUS DSMZ ACC 630<br>HBL1 Dr. Daniel Hodson, Cambridge Univ. CVCL_4213<br>HCT116 ATCC CCL-247<br>HEK293T Takara 632180<br>HMEC ATCC PCS-600-010<br>HT-29 ATCC HTB-38<br>H2444 ATCC CRL-5945<br>H69 ATCC HTB-119<br>H82 ATCC HTB-175<br>H146 Cytion 300182<br>H524 Cytion 305120<br>H446 Cytion 305049<br>H526 ATCC CRL-5811<br>JEKO1 DSMZ ACC 533<br>JJN3 DSMZ ACC 541<br>JURL-MK1 DSMZ ACC 532<br>Karpas-422 Sigma-Aldrich 6101702<br>KASUMI-2 DMSZ ACC 526<br>KCL22 DSMZ ACC 519<br>KE37 DSMZ ACC 46<br>KM-H2 DSMZ ACC 8<br>KOPN8 DMSZ ACC 552<br>L428 DSMZ ACC 197<br>LOVO ATCC CCL-229<br>L-Wnt3a cell line ATCC CRL-2647<br>MCF10A ATCC CRL10317<br>MCF12A ATCC CRL3598<br>MINO ATCC ACC 687<br>MV-4-11 ATCC CRL-9591<br>MOLM13 DSMZ ACC 554<br>MONO-MAC-6 DSMZ ACC 124<br>MOLT3 ATCC CRL-1552<br>NALM6 ATCC CRL-3273<br>OCI-LY10 Dr. Daniel Hodson, Cambridge Univ. CVCL_8795<br>RAJI DSMZ ACC 319<br>RAMOS DSMZ ACC 603<br>RCH-ACV DSMZ ACC 548<br>REC1 DSMZ ACC-584<br>SEM DSMZ ACC 546<br>SU-DHL4 DSMZ ACC 495<br>SU-DHL-2 DSMZ CRL-2956<br>SU-DHL-8 DSMZ CRL-2961<br>SW620 ATCC CCL-227<br>SW480 ATCC CCL-228<br>THP1 ATCC TIB-202<br>TOLEDO ATCC CRL-2631<br>TOM1 DMSZ ACC 578<br>U266 DSMZ ACC 9<br>U2932 DSMZ ACC 633<br>Z-138 DSMZ CRL-3001
Authentication	Cell lines and patient-derived xenografts were validated by STR DNA profiling analysis.
Mycoplasma contamination	Cells were tested negative for mycoplasma contamination with mycoplasma by detection kit (MycoAlert PLUS, LONZA).
Commonly misidentified lines<br>(See ICLAC register)	No misidentified cells were used.

Palaeontology and Archaeology

Specimen provenance	*Provide provenance information for specimens and describe permits that were obtained for the work (including the name of the issuing authority, the date of issue, and any identifying information). Permits should encompass collection and, where applicable, export.*

Specimen deposition	*Indicate where the specimens have been deposited to permit free access by other researchers.*
Dating methods	*If new dates are provided, describe how they were obtained (e.g. collection, storage, sample pretreatment and measurement), where they were obtained (i.e. lab name), the calibration program and the protocol for quality assurance OR state that no new dates are provided.*

☐ Tick this box to confirm that the raw and calibrated dates are available in the paper or in Supplementary Information.

Ethics oversight	*Identify the organization(s) that approved or provided guidance on the study protocol, OR state that no ethical approval or guidance was required and explain why not.*

Note that full information on the approval of the study protocol must also be provided in the manuscript.

Animals and other research organisms

Policy information about studies involving animals; ARRIVE guidelines recommended for reporting animal research, and Sex and Gender in Research

Laboratory animals	Ctnnb1fl/fl (004152), eGFP-Myc(021935), Gsk3bfl/fl(029592), NSG (005557), TQ-b-catenin(036014) , Mb1-Cre (020505) mice were purchased from Jackson Laboratory. Apcfl/+ mice were provided by Zhijian Qian. Vav-Cre Csnk1a fl/+ mice were provided by Benjamin Ebert. Vav-Cre Csnk1a1 mice were crossed with B6 mice for 2 generations to generate Csnk1a1fl/+ mice without Vav-Cre. Ctnnb1ex3fl strain was provided by Mark Taketo. To activate or delete beta-catenin in B cell precursors Mb1Cre mice were crossed to Ctnnb1ex3fl and Ctnnb1fl/fl mice respectively. For in-vivo activation of beta-catenin in normal B-cells, mice homozygous or heterozygous for the Ctnnb1ex3fl locus were used and since no significant differences were observed between homozygous and heterozygous mice, the exact genotype is not indicated. Both Cre-positive and negative animals were used as controls and no significant differences were observed between these two types of control animals. For modelling B-ALL transformation, mice heterozygous for the Ctnnb1ex3fl locus were used. For generating eGFP-Myc x TQ-beta-catenin mice eGFP-Myc and TQ-beta-catenin mice were crossed. Genotyping of the mice was done using primers described in Table S5. Vav-Cre Csnk1a1 mice were genotyped by Transnetyx. All animals were maintained in a specific pathogen free environment. Experiments were approved by the regional council in Freiburg , Germany (TVA: 35-9185.81/G-15/157) and Yale University, USA (Protocol: 20345) and carried out in accordance with the German Animal Welfare Act and Institutional Animal Care & Use Committee.
Wild animals	No wild animal was used.
Reporting on sex	Both male and female mice were used and we did not see significant difference between the groups.
Field-collected samples	Temperatures of 18-23°C with 40-60% humidity were maintained with 14-hour light/10-hour dark cycle. End-point for animal experiments are as follows: 1. Failure to eat food / drink water for 24 hours. 2. Failure to make normal postural adjustments / display normal behavior. 3. Tumor Burden (1.5 cm X 1.5c,X1.5 cm, tumor ulceration is NOT expected). If an animal either loses 25% of the initial body weight (or reaches 16g of body weight, regardless of the initial weight) or if we observe a weight loss of 15% on two sequential weight measurements, we euthanized the mouse immediately.
Ethics oversight	All mouse experiments were approved by the regional council in Freiburg (TVA: 35-9185.81/G-15/157), Germany or Yale University, USA (Protocol: 20345) and carried out in accordance with the German Animal Welfare Act and Institutional Animal Care & Use Committee.

Note that full information on the approval of the study protocol must also be provided in the manuscript.

Clinical data

Policy information about clinical studies

All manuscripts should comply with the ICMJE guidelines for publication of clinical research and a completed CONSORT checklist must be included with all submissions.

Clinical trial registration	*Provide the trial registration number from ClinicalTrials.gov or an equivalent agency.*
Study protocol	*Note where the full trial protocol can be accessed OR if not available, explain why.*
Data collection	*Describe the settings and locales of data collection, noting the time periods of recruitment and data collection.*
Outcomes	*Describe how you pre-defined primary and secondary outcome measures and how you assessed these measures.*

Dual use research of concern

Policy information about dual use research of concern

Hazards

Could the accidental, deliberate or reckless misuse of agents or technologies generated in the work, or the application of information presented in the manuscript, pose a threat to:

No	Yes	
☒	☐	Public health
☒	☐	National security
☒	☐	Crops and/or livestock
☒	☐	Ecosystems
☒	☐	Any other significant area

Experiments of concern

Does the work involve any of these experiments of concern:

No	Yes	
☒	☐	Demonstrate how to render a vaccine ineffective
☒	☐	Confer resistance to therapeutically useful antibiotics or antiviral agents
☒	☐	Enhance the virulence of a pathogen or render a nonpathogen virulent
☒	☐	Increase transmissibility of a pathogen
☒	☐	Alter the host range of a pathogen
☒	☐	Enable evasion of diagnostic/detection modalities
☒	☐	Enable the weaponization of a biological agent or toxin
☒	☐	Any other potentially harmful combination of experiments and agents

Plants

Seed stocks

Report on the source of all seed stocks or other plant material used. If applicable, state the seed stock centre and catalogue number. If plant specimens were collected from the field, describe the collection location, date and sampling procedures.

Novel plant genotypes

Describe the methods by which all novel plant genotypes were produced. This includes those generated by transgenic approaches, gene editing, chemical/radiation-based mutagenesis and hybridization. For transgenic lines, describe the transformation method, the number of independent lines analyzed and the generation upon which experiments were performed. For gene-edited lines, describe the editor used, the endogenous sequence targeted for editing, the targeting guide RNA sequence (if applicable) and how the editor was applied.

Authentication

Describe any authentication procedures for each seed stock used or novel genotype generated. Describe any experiments used to assess the effect of a mutation and, where applicable, how potential secondary effects (e.g. second site T-DNA insertions, mosiacism, off-target gene editing) were examined.

ChIP-seq

Data deposition

☒ Confirm that both raw and final processed data have been deposited in a public database such as GEO.

☒ Confirm that you have deposited or provided access to graph files (e.g. BED files) for the called peaks.

Data access links
May remain private before publication.

https://www.ncbi.nlm.nih.gov/geo/query/acc.cgi?acc=GSE196768

Files in database submission

GSM5900072	pooled_input_rep1
GSM5900073	ERT2_IKZF-WT_H3K27ac_rep1
GSM5900074	ERT2_IKZF-WT_H3K4me3_rep1
GSM5900075	ERT2_IKZF-WT_IKZF1_rep1
GSM5900076	ERT2_IKZF-WT_IKZF3_rep1
GSM5900077	CRE_IKZF-WT_H3K27ac_rep1
GSM5900078	CRE_IKZF-WT_H3K4me3_rep1
GSM5900079	CRE_IKZF-WT_IKZF1_rep1
GSM5900080	CRE_IKZF-WT_IKZF3_rep1
GSM5900081	ERT2_IKZF-KO_H3K27ac_rep1
GSM5900082	ERT2_IKZF-KO_H3K4me3_rep1
GSM5900083	CRE_IKZF-KO_H3K27ac_rep1
GSM5900084	CRE_IKZF-KO_H3K4me3_rep1
GSM5900085	ERT2_IKZF-WT_H3K27ac_rep2
GSM5900086	ERT2_IKZF-WT_H3K4me3_rep2
GSM5900087	ERT2_IKZF-WT_IKZF1_rep2
GSM5900088	ERT2_IKZF-WT_IKZF3_rep2
GSM5900089	CRE_IKZF-WT_H3K27ac_rep2
GSM5900090	CRE_IKZF-WT_H3K4me3_rep2

GSM5900091	CRE_IKZF-WT_IKZF1_rep2
GSM5900092	CRE_IKZF-WT_IKZF3_rep2
GSM5900093	ERT2_IKZF-KO_H3K27ac_rep2
GSM5900094	ERT2_IKZF-KO_H3K4me3_rep2
GSM5900095	CRE_IKZF-KO_H3K27ac_rep2
GSM5900096	CRE_IKZF-KO_H3K4me3_rep2
GSM5900097	CRE_IKZF-WT_CTNNB1_rep1
GSM5900098	CRE_IKZF-WT_CTNNB1_rep2
GSM5900099	CRE_IKZF-KO_CTNNB1_rep1
GSM5900100	CRE_IKZF-KO_CTNNB1_rep2
GSM5900101	pooled_input_rep2

Genome browser session
(e.g. UCSC)

No longer applicable

Methodology

Replicates

1- BCR-ABL1 transformed B-ALL cells from Ctnnb1 ex3 fl mice wild type for Ikzf1 and Ikzf3 (ERT2_IKZF-WT, n=2)
2-BCR-ABL1 transformed B-ALL cells from Ctnnb1 ex3 fl mice with activate b-catenin, wild type for Ikzf1 and Ikzf3 (CRE_IKZF-WT, n=2)
3- BCR-ABL1 transformed B-ALL cells from Ctnnb1 ex3 fl mice knock-out for Ikzf1 and Ikzf3 (ERT2 IKZF-KO, n=2)
4-BCR-ABL1 transformed B-ALL cells from Ctnnb1 ex3 fl mice with activate b-catenin, knock-out for Ikzf1 and Ikzf3 (CRE IKZF-KO, n=2)

Sequencing depth

Single ended 50bp long reads for all the reactions. Number of total reads and unique reads as given below.

ID	total_reads	uniquely_mapped
GSM5900072	23533677	22413756
GSM5900073	18519745	15411884
GSM5900074	27300198	19277347
GSM5900075	25756987	23791351
GSM5900076	28804077	28118134
GSM5900077	36408464	32492743
GSM5900078	26350969	20713100
GSM5900079	33687614	32726198
GSM5900080	29821838	29178337
GSM5900081	24798395	22702084
GSM5900082	22692580	18178350
GSM5900083	29703084	26033079
GSM5900084	23535452	18287961
GSM5900085	29406029	24254286
GSM5900086	25652148	17987511
GSM5900087	26514787	23683984
GSM5900088	26537057	25678393
GSM5900089	30779494	27184037
GSM5900090	28029768	21777798
GSM5900091	27273371	26187682
GSM5900092	36338524	35374744
GSM5900093	30909950	28306035
GSM5900094	21022047	16821512
GSM5900095	27382628	23960283
GSM5900096	17767517	13699623
GSM5900097	47123110	39485903
GSM5900098	51541064	35260651
GSM5900099	51500833	50020905
GSM5900100	45468349	37038801
GSM5900101	40757693	40298185

Antibodies

IKZF1 (GeneTex, GTX129438), IKZF3 (CST, D1C1E), H3K27ac (Active motif, #39133), H3K4me3 (Millipore, MC315), beta-catenin (Thermo, CAT-15)

Peak calling parameters

Peak calling was performed with MACS2 v2.2.7.1. Downstream analysis was performed in R – differential binding was analyzed with DiffBind v3.0.15 peaks with -log10 q-value > 10 in 2 or more conditions were retained after black and grey-listing. For transcription factors (IKZF1, IKZF3 and CTNNB1) within-peak normalization was applied, while whole-genome normalization was applied to histone modification data.

Data quality

Quality control was performed using FastQC v0.11.9, and ChIPQC.

Software

Reads were aligned with BWA v0.7.17 against the mouse genome (mm10/GRCm38, gencode vM24). Downstream analysis was performed in R – differential binding was analysed with DiffBind v3.0.15 with black and grey-listing within-peak normalization, annotation was performed with ChIPpeakAnno to the closest TSS excepting the BENC enhancer region which was manually annotated as described in Bahr et al. 2018.

Flow Cytometry

Plots

Confirm that:

- [x] The axis labels state the marker and fluorochrome used (e.g. CD4-FITC).
- [x] The axis scales are clearly visible. Include numbers along axes only for bottom left plot of group (a 'group' is an analysis of identical markers).
- [x] All plots are contour plots with outliers or pseudocolor plots.
- [x] A numerical value for number of cells or percentage (with statistics) is provided.

Methodology

Sample preparation	Cells were washed twice with PBS containing 2% FBS and blocked with Fc blocker (BD Biosciences) for 20 min on ice. Cells were stained with the appropriate antibodies or isotype controls for 30 min on ice. Cells were then washed and resuspended in PBS containing 0.75 µg ml-1 of DAPI to exclude dead cells.
Instrument	LSRFortessa X-20 or FACSSympony A3 flow cytometer (BD Biosciences) were used for FACS analysis. FACSAria III or FACSAria Fusion (BD Biosciences) were used for fluorescence based cell sorting experiments.
Software	FACS data were acquired with FACS Diva and analyzed with FlowJo software (FlowJo 10.10.0, LLC).
Cell population abundance	At least 10,000 cells per sample were recorded for growth competitions. For analyzing rare populations at least 200,000 cells were recorded. For sorting experiments purity was determined by comparison with negative control and sorting purity was around 99%.
Gating strategy	FCS-A/SSC-A gating was performed to exclude debris. Then, Single cells were selected by SSA-A/SSC-H gate and DAPI negative lymphocytes were gated for further analysis. The Boundaries between “positive” and “negative” were defined by comparison with isotype/negative control.

- [x] Tick this box to confirm that a figure exemplifying the gating strategy is provided in the Supplementary Information.

Magnetic resonance imaging

Experimental design

Design type	*Indicate task or resting state; event-related or block design.*
Design specifications	*Specify the number of blocks, trials or experimental units per session and/or subject, and specify the length of each trial or block (if trials are blocked) and interval between trials.*
Behavioral performance measures	*State number and/or type of variables recorded (e.g. correct button press, response time) and what statistics were used to establish that the subjects were performing the task as expected (e.g. mean, range, and/or standard deviation across subjects).*

Acquisition

Imaging type(s)	*Specify: functional, structural, diffusion, perfusion.*
Field strength	*Specify in Tesla*
Sequence & imaging parameters	*Specify the pulse sequence type (gradient echo, spin echo, etc.), imaging type (EPI, spiral, etc.), field of view, matrix size, slice thickness, orientation and TE/TR/flip angle.*
Area of acquisition	*State whether a whole brain scan was used OR define the area of acquisition, describing how the region was determined.*

Diffusion MRI ☐ Used ☐ Not used

Preprocessing

Preprocessing software	*Provide detail on software version and revision number and on specific parameters (model/functions, brain extraction, segmentation, smoothing kernel size, etc.).*
Normalization	*If data were normalized/standardized, describe the approach(es): specify linear or non-linear and define image types used for transformation OR indicate that data were not normalized and explain rationale for lack of normalization.*
Normalization template	*Describe the template used for normalization/transformation, specifying subject space or group standardized space (e.g. original Talairach, MNI305, ICBM152) OR indicate that the data were not normalized.*

Noise and artifact removal | *Describe your procedure(s) for artifact and structured noise removal, specifying motion parameters, tissue signals and physiological signals (heart rate, respiration).*

Volume censoring | *Define your software and/or method and criteria for volume censoring, and state the extent of such censoring.*

Statistical modeling & inference

Model type and settings | *Specify type (mass univariate, multivariate, RSA, predictive, etc.) and describe essential details of the model at the first and second levels (e.g. fixed, random or mixed effects; drift or auto-correlation).*

Effect(s) tested | *Define precise effect in terms of the task or stimulus conditions instead of psychological concepts and indicate whether ANOVA or factorial designs were used.*

Specify type of analysis: ☐ Whole brain ☐ ROI-based ☐ Both

Statistic type for inference (See Eklund et al. 2016) | *Specify voxel-wise or cluster-wise and report all relevant parameters for cluster-wise methods.*

Correction | *Describe the type of correction and how it is obtained for multiple comparisons (e.g. FWE, FDR, permutation or Monte Carlo).*

Models & analysis

n/a	Involved in the study
☒	☐ Functional and/or effective connectivity
☒	☐ Graph analysis
☒	☐ Multivariate modeling or predictive analysis
