# Supplementary material for: Targeting β-catenin degradation with GSK3β inhibitors induces cell death in acute lymphoblastic leukemia
Source: Nat Cancer. 2026 Jan 8;7(1):150–68. doi: 10.1038/s43018-025-01093-z (PMC12858398; doi:10.1038/s43018-025-01093-z)

*Fig. 1b*

Left

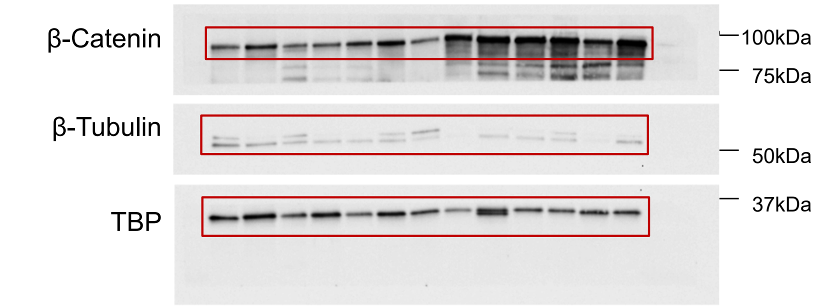


*Fig. 1b*

Middle

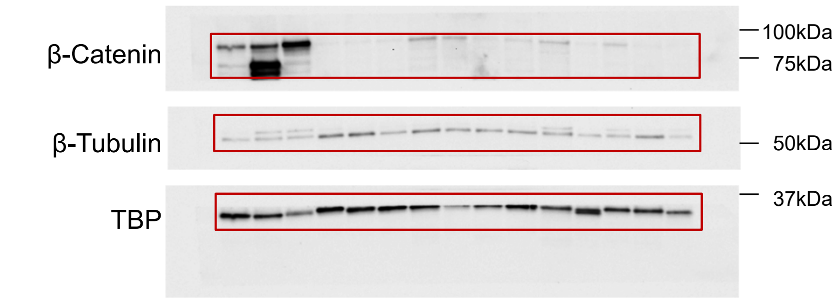


*Fig. 1b*

Right

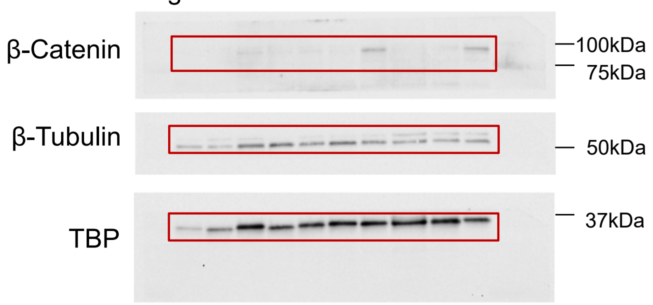


*Fig.1g*

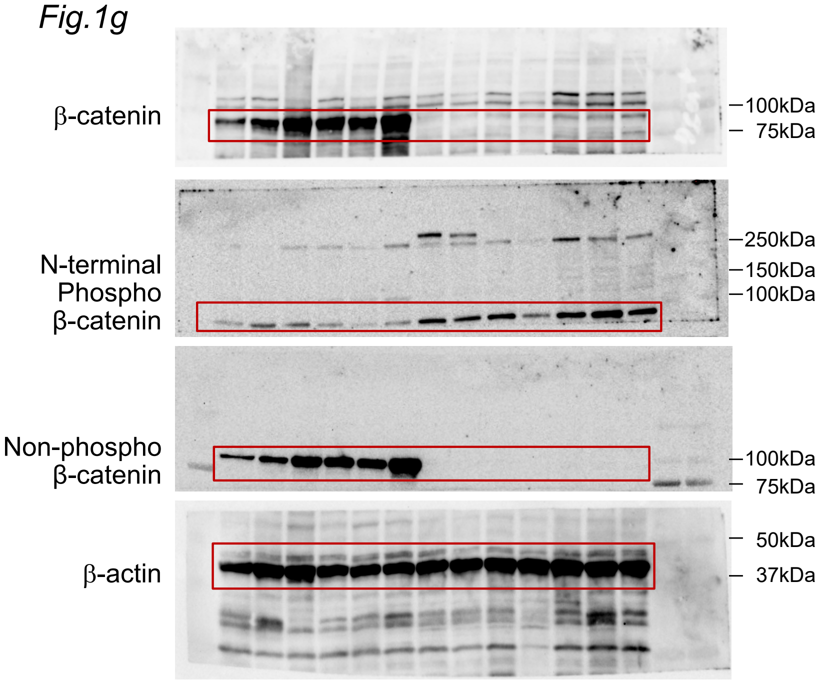

*Fig.2k*

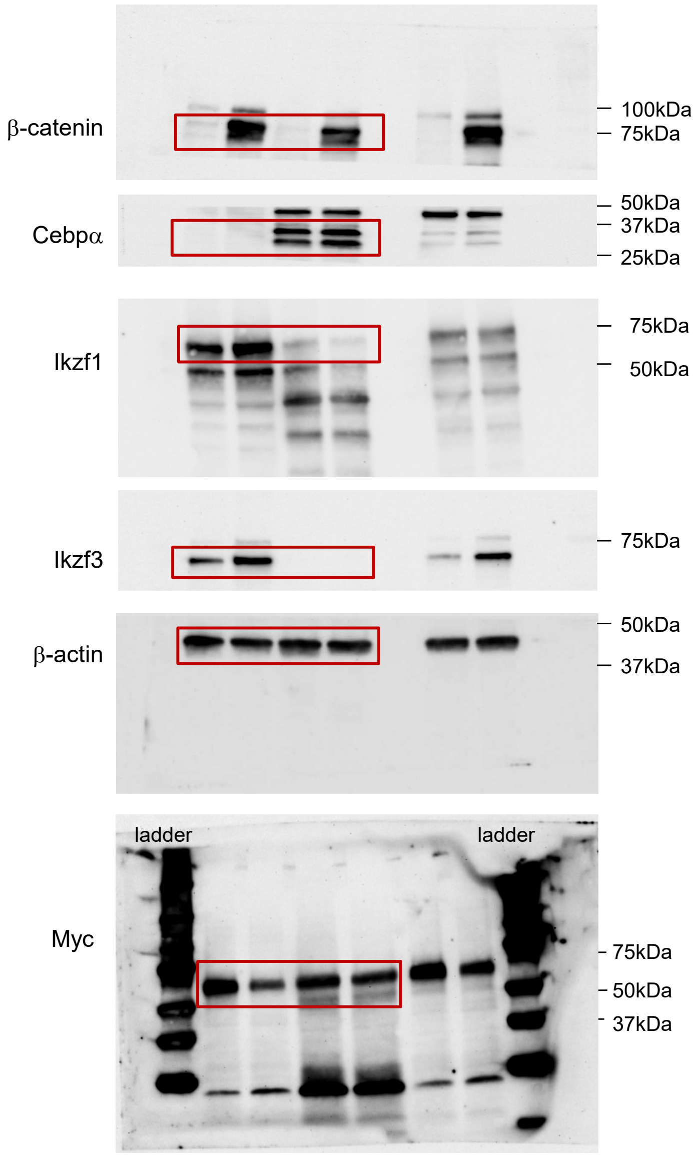
β-catenin
100kDa
75kDa
Cebpα
50kDa
37kDa
25kDa
Ikzf1
75kDa
50kDa
Ikzf3
75kDa
β-actin
50kDa
37kDa
ladder
ladder
Myc
75kDa
50kDa
37kDa

*Fig. 3c*

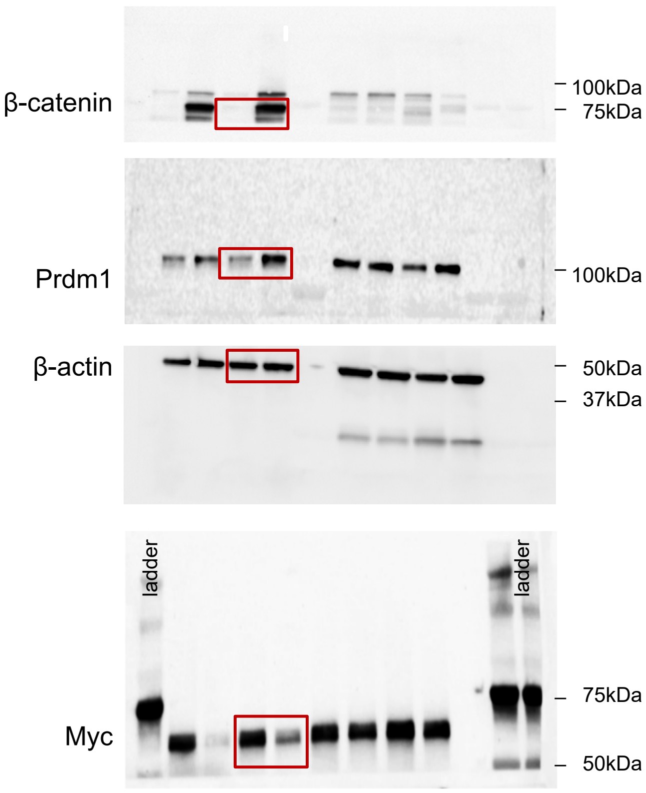


*Fig. 3e*

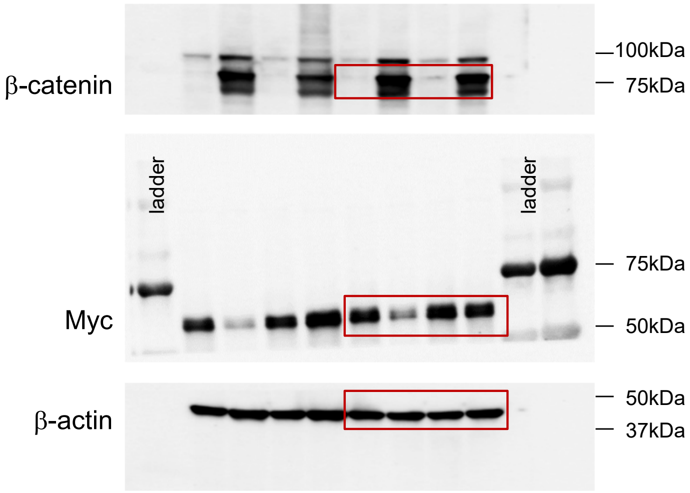


*Fig. 3h*

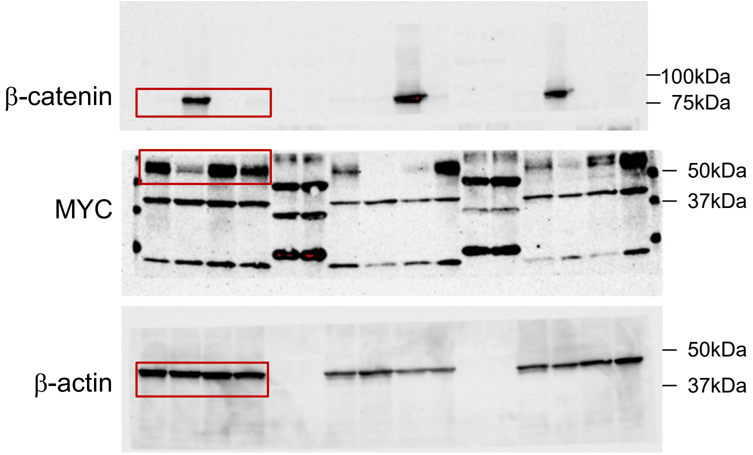


*Fig. 3i*

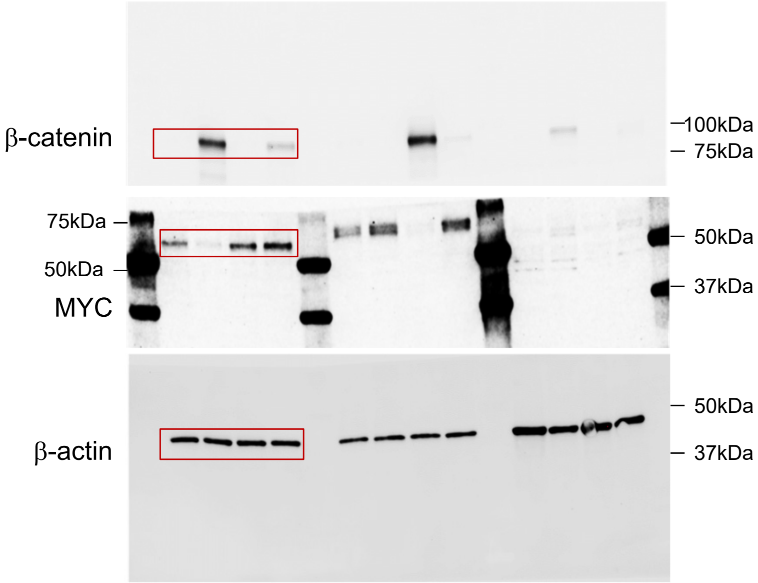

*Fig. 4b*

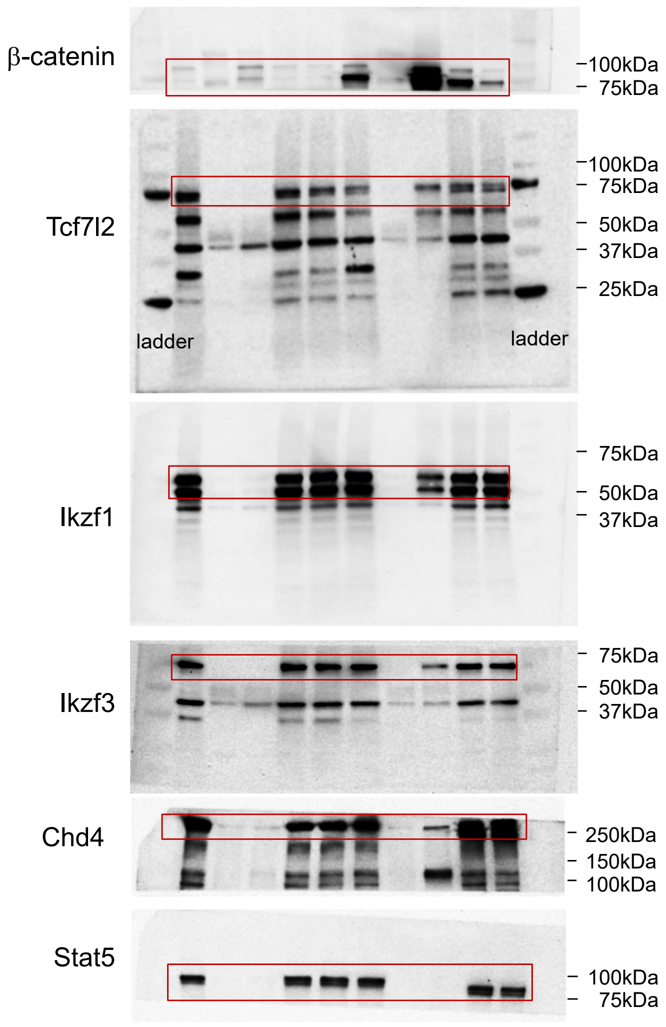


*Fig. 4d*

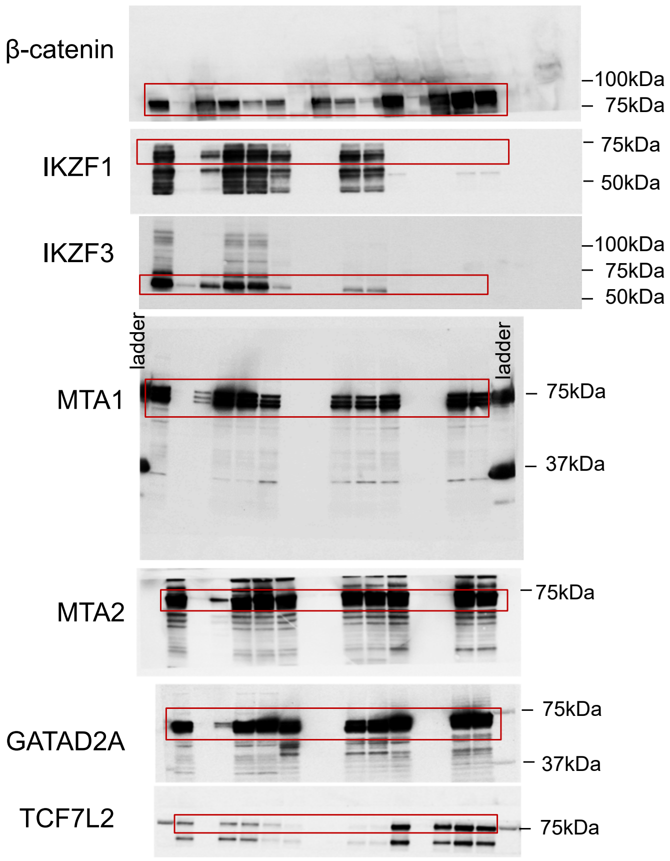


*Fig. 4h*

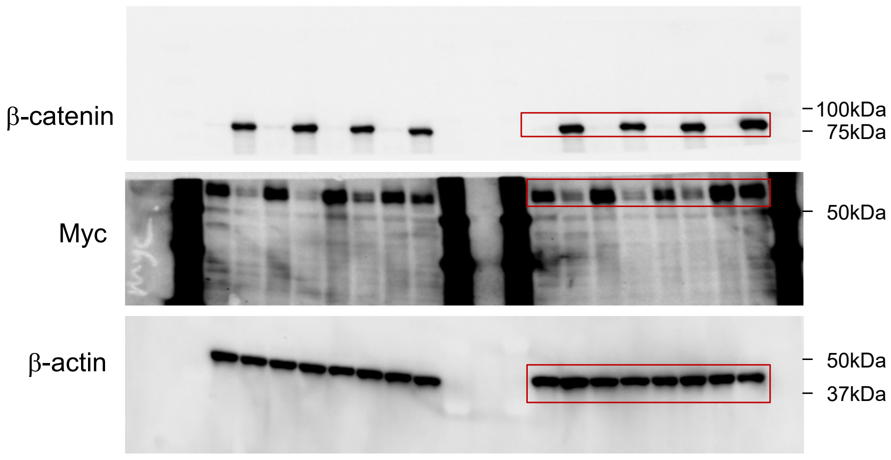


*Fig. 4h*

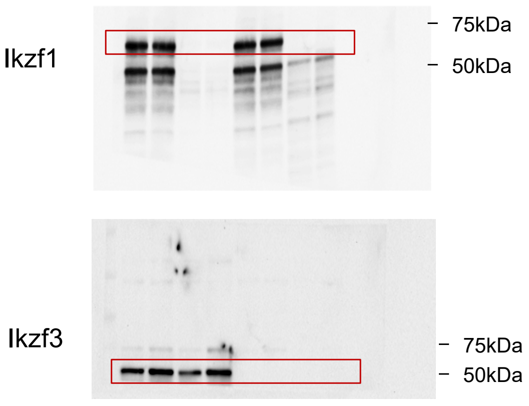

*Fig. 5g*

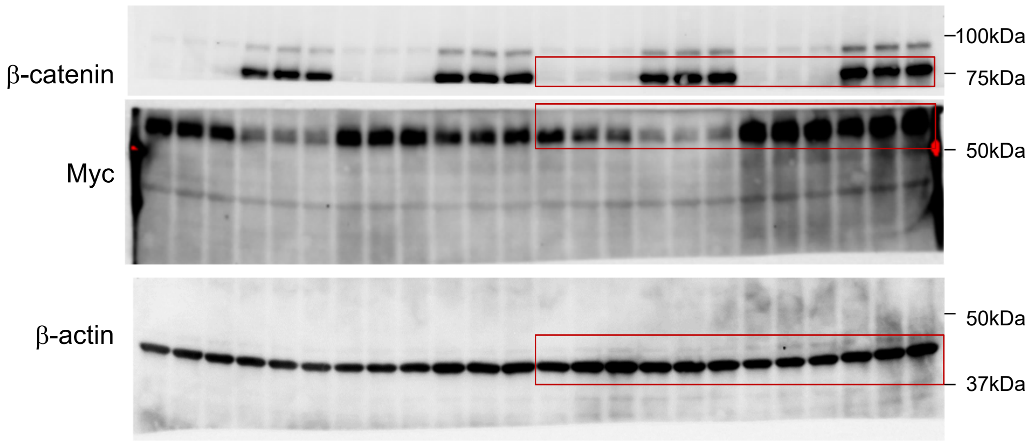

*Fig. 6d*

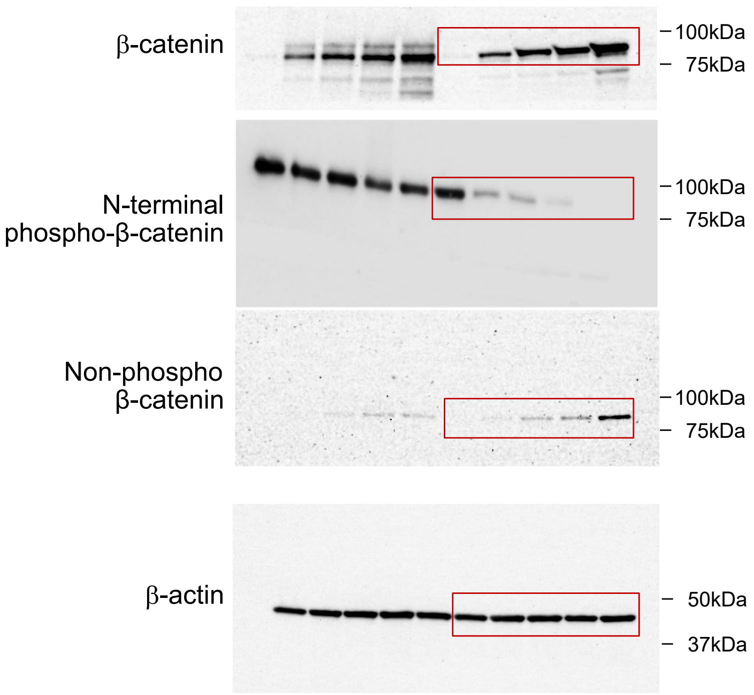

β-catenin
100kDa
75kDa
N-terminal phospho-β-catenin
100kDa
75kDa
Non-phospho β-catenin
100kDa
75kDa
β-actin
50kDa
37kDa


*Fig. 6f*

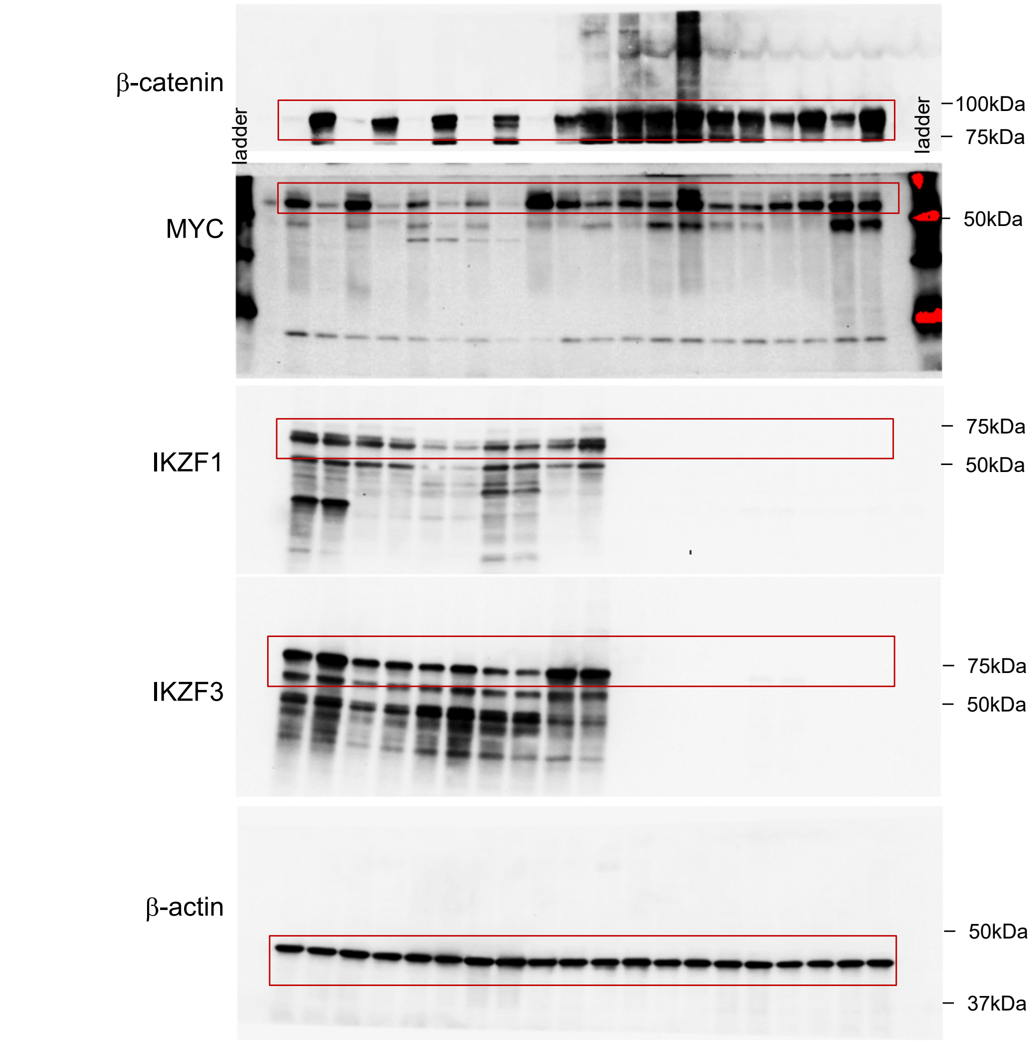

β-catenin
ladder
ladder
100kDa
75kDa
MYC
50kDa
IKZF1
75kDa
50kDa
IKZF3
75kDa
50kDa
β-actin
50kDa
37kDa

*Extended Data Fig. 2e*

Left

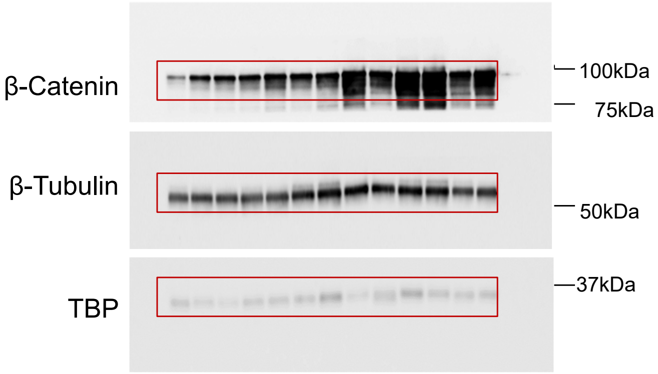


*Extended Data Fig. 2e*

Middle

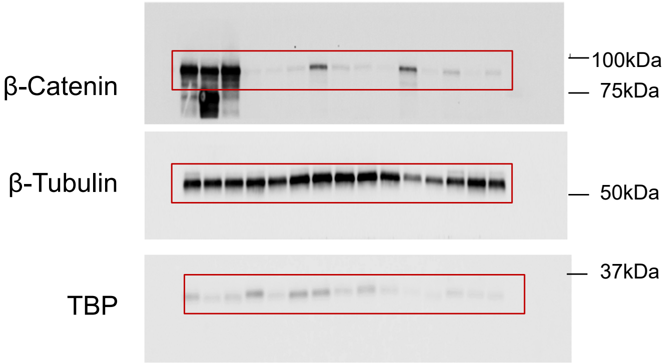


*Extended Data Fig. 2e*

Right

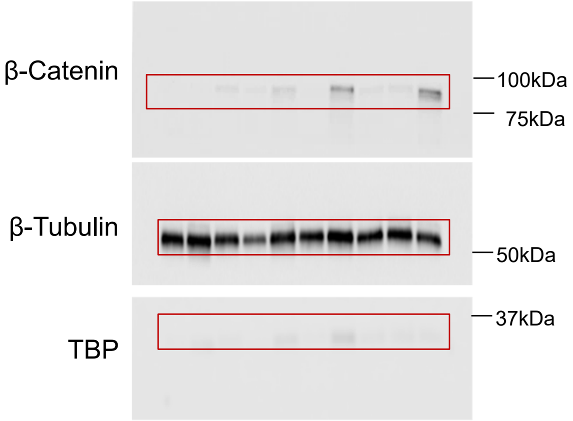

*Extended Data Fig. 4a*

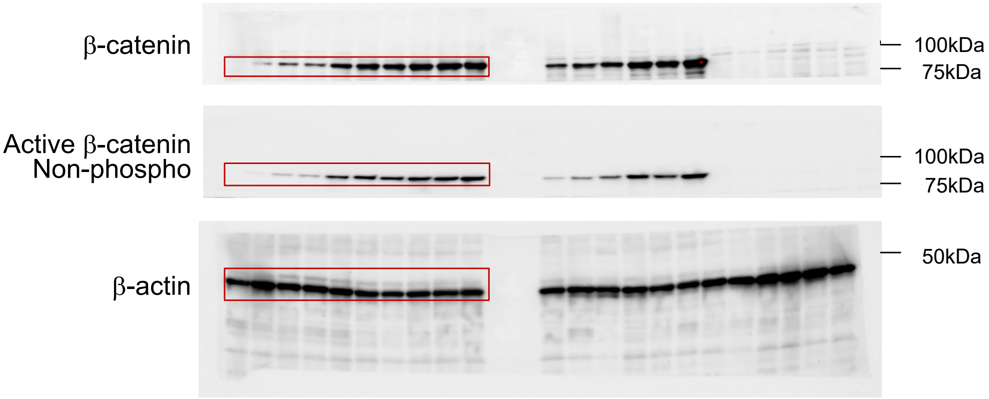


*Extended Data Fig. 4d*

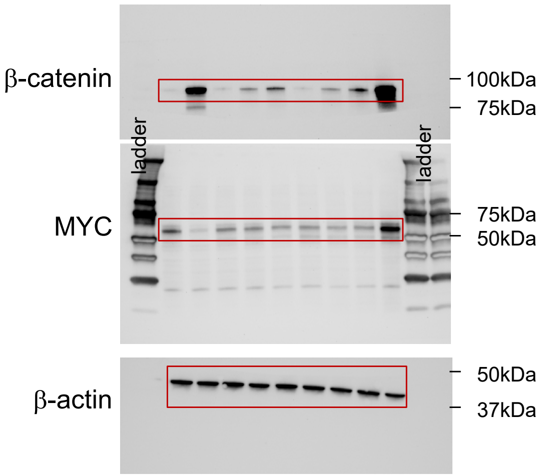

*Extended Data Fig. 5a*

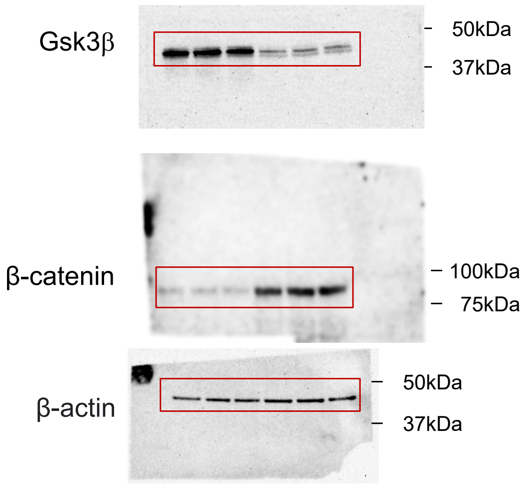


*Extended Data Fig. 5b*

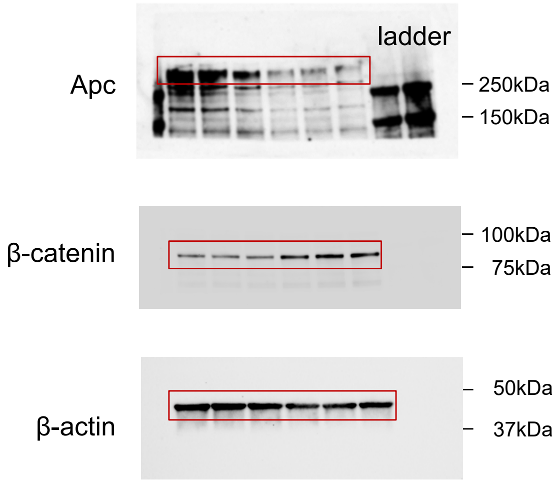


*Extended Data Fig. 5c*

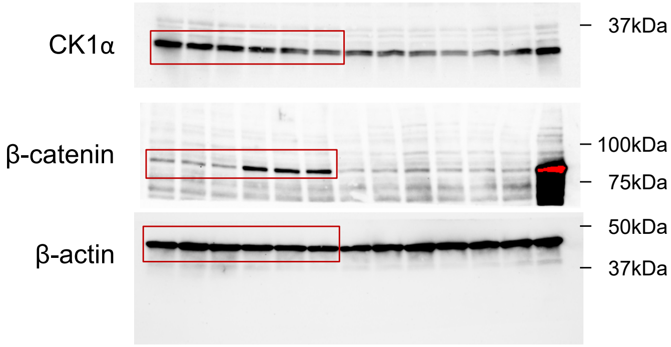


*Extended Data Fig. 5d*

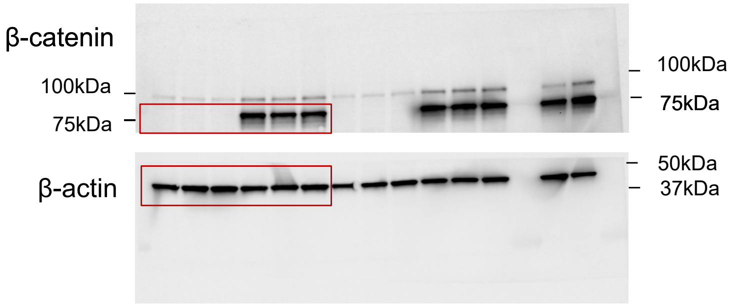

*Extended Data Fig. 6b*

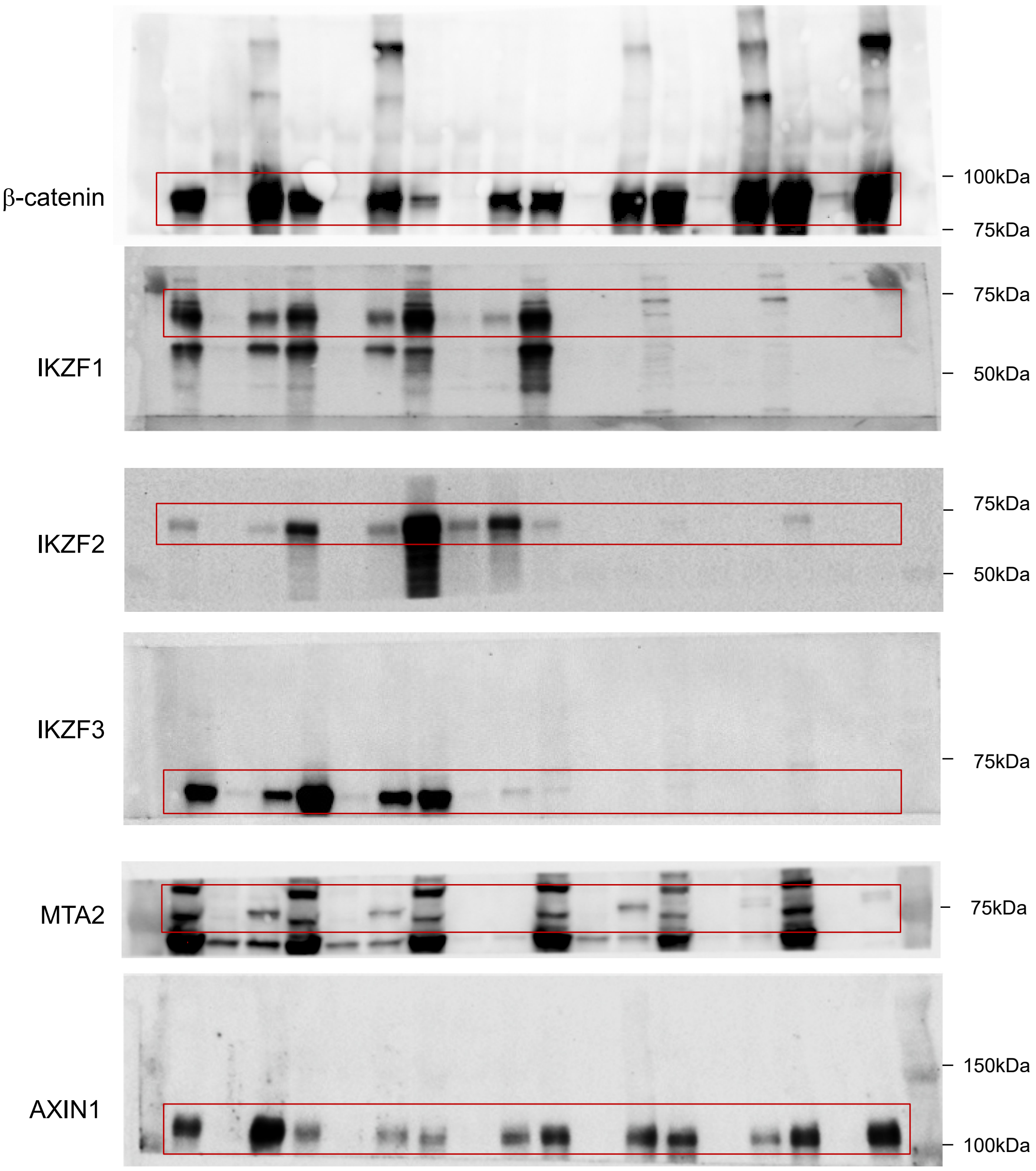

*Extended Data Fig. 8b*

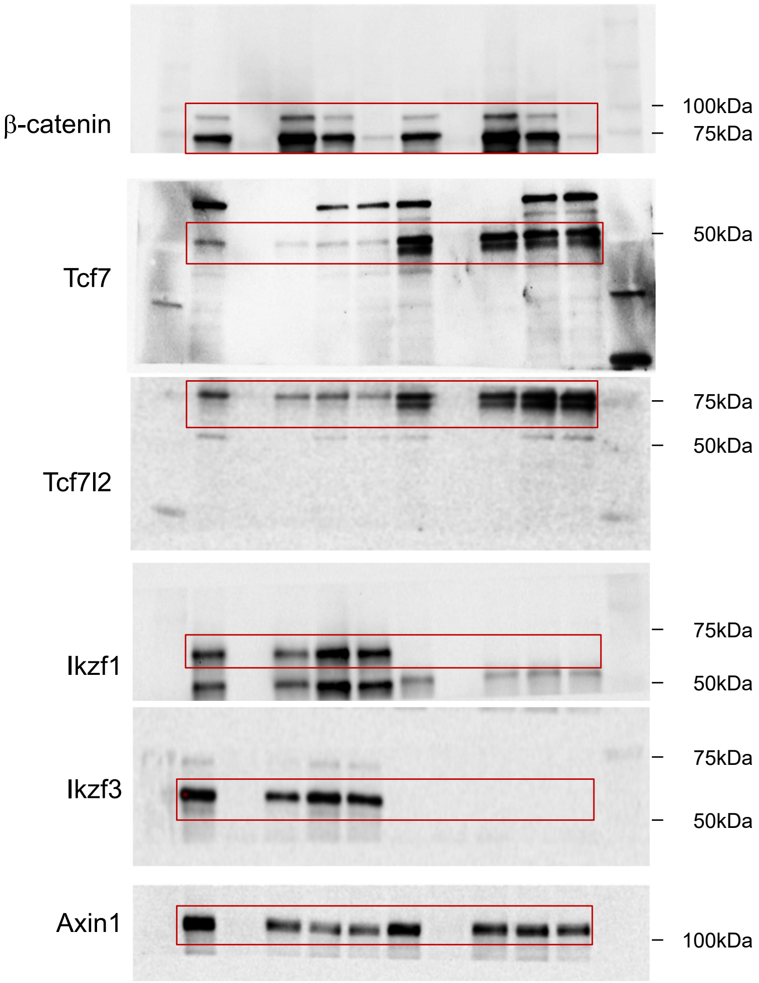

Supplement: Supplementary file 5 — Unprocessed western blots and/or gels. [file 43018_2025_1093_MOESM5_ESM.pdf]
